# Innovative and cost-effective upgrading of crude biogenic pyrolysis oil using low-cost adsorbents and petroleum ether

**DOI:** 10.1007/s11356-025-37268-5

**Published:** 2025-12-17

**Authors:** Akhil Mohan, Alan Al-Wandi, Åsa Emmer, Klas Engvall, Mats Jonsson

**Affiliations:** 1 Department of Chemical Engineering, KTH Royal Institute of Technology, 100 44 Stockholm, Sweden; 2 Department of Chemistry, KTH Royal Institute of Technology, 100 44 Stockholm, Sweden

**Keywords:** Pyrolysis, Crude biogenic oil, Separation strategies, Upgrading, Analytical techniques

## Abstract

The upgrading of crude biogenic pyrolysis oil (CO) was carried out using a simple scalable, inexpensive upgrading strategy based on the use of various adsorbents. The upgraded oil and the efficiency of the process were extensively characterized using various analytical techniques. The preliminary objective of the study was to identify the best adsorbent for refining CO. The adsorbents were characterized using scanning electron microscopy (SEM) and a Micromeritics 3Flex Instrument to determine the morphology and surface area of the adsorbents used. Crude and upgraded oil were extensively characterized to understand chemical composition, stability, and thermal properties. The importance of the study is to remove oxygenated compounds in CO using industrial waste adsorbents and solvent. The present upgrading strategy separates CO into an upper homogeneous soluble phase in petroleum ether and a lower non-homogeneous insoluble phase in petroleum ether. Oxygenates are reduced from 40.13 wt.% to 0.14 wt.% with the use of calcium hydroxide as an adsorbent and petroleum ether as a solvent. Finally, a discussion on the overview of the upgradation strategy is briefly summarized at the end of the manuscript.

**Graphical Abstract:**

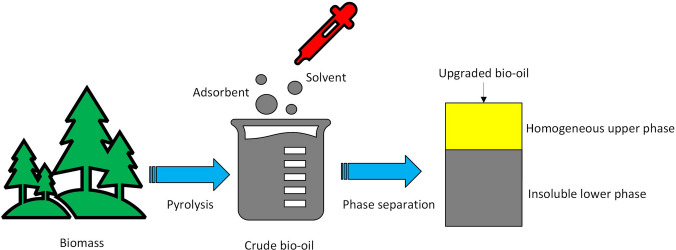

**Supplementary Information:**

The online version contains supplementary material available at 10.1007/s11356-025-37268-5.

## Introduction

The dependence on fossil reserves and the escalation in the human population growth have surged researchers to find alternative options to recover fuels and chemicals from biomass. As per the recent statistics from the International Energy Agency IEA (2023), statistics from 1900 to 2023 show a shoot-up in the carbon dioxide emission trend, reaching 37.4 Gt per year in 2023 (including the emission from energy combustion, industrial processes, and flaring). The release of CO_2_ with other anthropogenic air pollutants is responsible for causing climate change and thereby also reducing biodiversity. Thus, there is a strong global societal drive to move towards clean energy technologies to convert biomass into value-added fuels. Pyrolysis is a promising thermochemical pathway to depolymerize lignocellulosic biomass in an oxygen-free atmosphere at 400–600 °C. The byproducts from thermal pyrolysis, such as char and non-condensable gas, make the process more attractive than existing thermochemical conversion methods. The black-coloured biogenic oil with high viscosity obtained from the condensation system cannot be directly utilized in mechanical systems due to its deleterious properties such as low heating value, high amount of oxygenates, high acid content, and pungent odour (Bridgewater 2012; Drugkar et al. 2022). The storage of bio-oil for a long period in industries can cause polymerization of aldehydes that can react with acidic functionalities in bio-oil. This may cause thick hard deposits at the surface of the collection vessels. Thus, the upgrading of crude biogenic oil is necessary to address the above-mentioned challenges.

Previous literature studies regarding various upgradation technologies and separation techniques showed that cotton wool-filled granular filters for volatiles upgrading (Paenpong et al. 2013), distillation (Zuo et al. 2009; Elkasabi et al. 2023), organic solvent extraction (Drugkar et al. 2022; Diebold et al. 1997; Wei et al. 2015; Panwar and Paul 2020), esterification or neutralization (Chong et al. 2020), emulsification (Ikura et al. 1998; Chaiaramonti et al. 2003), fractional condensation through vapour phase upgrading (Westerhof et al. 2011), catalytic cracking (Ambursa et al. 2021), catalytic reforming (Hew et al. 2010; Zhang et al. 2022), steam reforming (Adeniyi et al. 2019), hydrotreating (Elliot et al. 2012; Han et al. 2019; Zacher et al. 2014), and the use of adsorbents and solvents (Naik et al. 2010; Mohan et al. 2019, 2021) are used for the upgrading of pyrolysis volatiles and condensed fractions from the pyrolysis reaction. Today, catalytic pyrolysis is widely recognized as an effective method for improving the quality of pyrolysis oil without the need for further upgrading processes. The benefits and limitations of catalytic pyrolysis and the proposed upgradation strategy are shown in Table 1 (Cai et al. 2024). The benchmarking of solvent upgrading and the present strategy is detailed in Table 2.

**Table 1:** Benefits and limitations of catalytic pyrolysis and present strategies (Cai et al. 2024)

Comparison	Catalytic pyrolysis	Present strategy
Benefits	• Reduce activation energy and improve process efficiency • Improve product distribution and increase the selectivity of products • Promote deoxygenation and aromatization	• Simple strategy to reduce oxygen content and separate value-added products • Easy to scale-up • Least energy-intensive and no need for huge capital investment • More selective to the specific products for the pharmaceutical and nutraceutical industries • Based on the principle of phase separation • No need for regeneration, if the waste byproduct can be used as an adsorbent. The solvent can be regenerated using rotary evaporation
Limitations	• Deterioration of the catalyst over time • High process cost due to catalyst use and regeneration • Coke formation • Energy-intensive with high capital expenditure • Complex chemical composition due to various reaction pathways • Need to control various parameters, and the mechanism is complex	• Need for biogenic-based solvents • Process efficiency and mass transfer of batch scale needs to be improved using various other possibilities (column-based or packed column strategies) • Yield needs to be improved with minimal loss by using other possibilities (packed bed column)

**Table 2:** Benchmarking of the present study with recent upgrading methods (Usman et al. 2023)

**Benchmarking parameters**	Shezi et al. 2025** (reference 1)**	Naidu et al. 2025** (reference 2)**	Tuppurainen et al. 2026** (reference 3)**	**Present study**
Upgrading principle	Solvent or catalyst-assisted hydrotreating	Liquid–liquid extraction	Catalytic hydrodeoxygenation with fractional distillation	Solvent and adsorbent-assisted phase separation
Bio-oil: solvent	4:1	1:1	-	1:6
Solvent/adsorbents/catalyst/reactor configuration	Diethyl ether, ethanol, methanol, and isopropyl alcohol Ru/C Batch scale	Hexane, ethyl acetate, chloroform and toluene Batch scale	Cobalt molybdenum disulfide catalyst Two-stage hydrotreating continuous pilot-scale reactor	Waste industrial adsorbents/petroleum ether Batch scale
Process conditions	68.94 bar, 500 rpm, 200 °C, 4 h	Normal P, T^*^, 3 h	400 °C, > 100 bar	Normal P, T^*^ 500 rpm, 6 h
Process efficiency (%)	Deoxygenation using various solvents: Diethyl ether: 19% Ethanol: 29% Methanol: 24% Isopropyl alcohol: 21%	High phenolics recovery in n-hexane (41.38%)	Deoxygenation: 97.31%	Deoxygenation using calcium hydroxide and petroleum ether as a solvent: 99.65%
Economic viability	High capital investment due to pressurized hydrogen	Simple batch-scale system operating at normal atmosphere	Production cost: 1754 EUR/tonne Capital expenditure: 153.5 million euros	Simple batch scale system operating at normal pressure and temperature
Environment impact	Toxic solvents	Toxic solvent	No solvent, pressurized hydrogen	Toxic solvent
Product applications	Transportation fuels	Value-added chemicals	Transportation fuels (gasoline, jet and diesel fractions)	Value-added compounds/fuel additives
Scale-up limitations	High operational investment and capital expenditure Recovery of used catalyst	Low product yield	High operational investment Energy expenditure Required pressurized hydrogen	Low mass transfer Low product yield
Recommendations and suggestions for scale-up from an industry perspective	Difficult Use of biogenic solvent	Difficult Use of biogenic solvent	Difficult Use of alternate approaches without the use of hydrogen	Easy Use of biogenic solvent A phase separator with a continuous scale adsorption system is recommended Use of a long-packed column in series is recommended to avoid mass transfer loss in a batch-scale process

^*^P, T: pressure, temperature

Among all the upgrading and separation technologies presented in the literature, the huge capital investment, thermal sensitivity, and operating expenditures (temperature, pressure, and catalyst) increase the overall cost of the upgrading system and hinder the scale-up from an industry perspective. No studies are reported in the literature on the phase separation of bio-oil using industrial waste adsorbents and a solvent for removing oxygen-containing compounds from bio-oil. Previous literature portrays selective adsorption and preferential solubility as a principle for upgrading crude plastic and tyre pyrolysis oil (Mohan et al. 2019, 2021; Paiva et al. 2023). The focus of the previous investigations was to remove sulphur-containing compounds from crude tyre pyrolysis oil.

In our previous studies on upgrading crude tyre pyrolysis oil, we used commercial silica gel as an adsorbent and petroleum ether as a solvent. The main focus of the previous investigation was to adsorb sulfur-containing compounds in the tyre pyrolysis oil through a batch-scale adsorption process for utilization as a fuel in a single-cylinder diesel engine. Compared to our previous investigation, the present study focuses on a novel phase separation approach for upgrading bio-oil by using petroleum ether as a solvent and various cheap/industrial waste adsorbents for the separation of value-added compounds. Thus, phase separation using petroleum ether as a solvent is an efficient way of upgrading bio-oil; this is the novelty of the present study. In short, we have previously used adsorption as a principle to remove sulphur-containing compounds from tyre pyrolysis oil, whereas the present study utilizes phase separation as a principle to remove oxygen-containing compounds from biogenic pyrolysis oil.

The innovative aspect of the present work is the separation of value-added compounds in biogenic pyrolysis oil using cheap adsorbents (waste byproducts from industries) and solvent (petroleum ether). Phase separation is used as a novel contribution in the proposed study to separate phenolics and aromatics. The present study is an extension of our previous investigations on refining crude tyre pyrolysis oil into diesel range fractions using column and batch scale refining strategies to improve the diversity from an application point of view (Mohan et al. 2019, 2021). The general objective of the study is to refine crude biogenic oil through the principle of separation using various low-cost adsorbents and solvents, to characterize crude and upgraded oil using various analytical techniques to determine the chemical composition, stability, and thermal properties. The main objective of the present study is to separate value-added compounds from crude biogenic oil for utilization in the pharmaceutical and nutraceutical industries. The novelty in the present study is the utilization of industrial waste products (red mud, bentonite, dolomite, used silica, and new silica) and a commercial adsorbent (calcium hydroxide) as adsorbents for the upgrading of crude biogenic oil with a focus on oxygen removal. The basis of the selection of petroleum ether as a solvent is its low cost, and because it is non-polar, easily available, and offers a higher efficiency of liquid extraction compared to polar solvents (Drugkar et al. 2022). The purpose of the study is to separate value-added compounds from crude biogenic oil and improve the thermal stability (lower wax deposition) of upgraded pyrolysis oil. A study by Mora et al. (2023) demonstrated that dialysis with column chromatography can separate value-added compounds such as acids, antioxidants (phenolics), and benzene derivatives. These compounds can be utilized in the pharmaceutical and nutraceutical industries (chemicals and food additives).

## Materials and methods

All the chemicals and petroleum ether used in this study were received and purchased commercially from VWR International. Crude biogenic pyrolysis oil was purchased from BTG (Biomass Technology Group) in the Netherlands and stored in a refrigerator at ~ 0 °C to avoid phase separation during long-term storage. Petroleum ether with a boiling range of 100–120 °C was used throughout. The red mud, dolomite, and used and new silica are industrial waste products obtained from Meva Energy Sweden, used as adsorbents in the upgrading experiments. Calcium hydroxide (CAS: 1305–62-0) was purchased from Sigma Aldrich, and bentonite (CAS: 1302–78-9) was purchased from Thermo Scientific.

### Scrutiny of pyrolysis oil from BTG

The present investigations used a crude biogenic pyrolysis oil collected from BTG as the feed. The pyrolytic oil was obtained from the thermal depolymerization of clean pine sawdust using a rotating cone-type reactor (https://www.btg-bioliquids.com/). The wood particles with an average particle size of 3 mm were pyrolyzed at a temperature of 500 °C at atmospheric pressure, gas residence time (≤ 2 s). The volatiles were condensed in a single-stage condensation system to yield 65 wt. % crude biogenic pyrolysis oil defined as CO. The optimized pyrolysis conditions were taken from the BTG process (https://www.btg-bioliquids.com/). The purchased oil was stored in a refrigerator to avoid any phase separation or creaming. CO is defined as a thick, dark-coloured, viscous liquid with a pungent smell and contains high particulates settled at the bottom of storage containers. The extensive information on the BTG process and process conditions is shown in the supplementary information (Sect. "Conclusions").

### Separation strategies for upgrading biogenic pyrolysis oil

In the previous study on the upgrading of crude tyre pyrolysis oil using silica gel, in which one of the co-authors of the present study was involved, process optimization was carried out. However, the procedure was not described in the publication. To allow for a direct comparison, we used the same optimized parameters in the present study. The preliminary focus of the present study was to test and demonstrate our previous proof-of-concept for upgrading bio-oil for oxygen removal to improve the diversity of applications using various pyrolysis oils (Widjaja et al. 2018; Cai et al. 2019; Mohan et al. 2019, 2021). In the present study, we included other adsorbents (calcium hydroxide, redmud, bentonite, dolomite, used silica, new silica).

The upgrading test rig consisted of a mixing assembly, a filtration setup with a filter, and an evaporation system. All refining experiments of crude biogenic pyrolysis oil were performed in the Division of Process Technology, Royal Institute of Technology, KTH, Stockholm, Sweden. The detailed specifications of the upgrading system are listed in Table 3. All borosilicate glassware is procured and connected manually for the purification strategy.

**Table 3:** Specification of the upgradation facility

Component	Specifications
Membrane filter	The pore size of 0.45 µm, Pall Corporation
Filtration assembly	Duran, borosilicate glass, 2L capacity
Vacuum pump	VWR VCP 80, 190 W, 50 kPa
Magnetic stirrer	IKA, 500 rpm, 25 °C
Stirrer	Crossbar PTFE
Rotary evaporator	IKA DEST
Tubing or connectors	Silicon
Feed volume (crude oil)	20 mL
Adsorbent	20 gm
Solvent	100 mL
Yield of upgraded oil	20 wt.% ± 1
Losses	38 wt. % ± 5

**Fig. 1:**
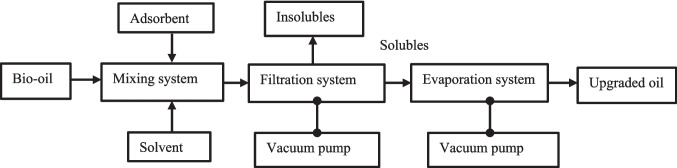
Schematic of upgradation test-rig at KTH Royal Institute of Technology

Figure 1 schematically shows the upgrading test rig for refining crude biogenic oils. The strategy used in the present investigation was adapted from the research on the upgrading of crude tyre pyrolysis oil, with a focus on extending the upgrading strategies to biogenic feedstocks and on oxygen removal (Mohan et al. 2021). In the previous investigation, studies focused on upgrading the crude tyre pyrolysis oil using a similar refining strategy. The upgrading experiments for cleaning crude biogenic pyrolysis oil (CO) samples consist of three stages, including mixing, filtration, and evaporation. 20 mL of crude biogenic pyrolysis oil was mixed with 100 mL of petroleum ether and 20 g of adsorbents in a beaker and stirred for 6 h with a stirring speed of 500 rpm at room temperature. The mixed solution was decanted into a Millipore filtration system connected to a filter and vacuum pump assembly to extract the soluble phase of crude oil with solvent. The collection fraction is termed upgraded pyrolysis oil samples.

### Characterization of adsorbents, crude, and upgraded oil samples

#### BET surface area analyzer

The surface properties of the various adsorbents were determined from nitrogen adsorption–desorption isotherms, using a Micromeritics 3Flex instrument (Version 6.03). Before the measurements, the samples (ca. 0.1 g) were degassed at 400 °C for 120 min under ultra-high vacuum (10^−6^ mbar). A known volume of gas is passed through a certain volume containing adsorbent at a constant temperature. As the adsorption takes place, the pressure falls until the equilibrium is reached. The amount of gas adsorbed at equilibrium will be the difference between the gas admitted and the amount of gas filled in the space around the adsorbent. The adsorption isotherm is usually constructed point by point by admission of successive charging of gas to the adsorbent by a dosing technique and appropriate gas laws. The specific surface area was estimated using the Brunauer-Emmet-Teller (BET) isotherm. The total pore surface area and total pore volume were calculated at a relative pressure (P/P_o_) of 1.0.

#### Scanning electron microscopy with energy dispersive x-ray spectroscopy (SEM-EDAX)

SEM imaging was performed using an Apreo 2S (Thermo Scientific) scanning electron microscope at an electron gun voltage of 1–2 kV, a current of 13–25 pA, and a working distance of 4.5 mm (bottom of the SEM column and top surface of the sample) and using a T1, T2, and T3 detector to check the contrast at various magnifications. EDAX imaging was carried out based on the principle of castaings formulae using Chemi SEM. The Quant maps are generated using T1, T2, T3, and Everhart–Thornley detectors. EDAX imaging used a working distance of 10 mm. A quant map from EDAX shows the lateral distribution of sample morphology. Before imaging, all samples were powdered and loaded on carbon tape.

#### Elemental analysis (CHNS/O)

Elemental analysis was performed in an Elementar Vario EL Cube (serial number 1930BC1003) instrument with a TCD detector. Approximately 20 mg of the sample was weighed into the tin capsule. The folded capsule was flashed with nitrogen and placed into the quartz glass pyrolysis tube using an autosampler. The oxygen-containing radicals formed in the pyrolysis tube were converted quantitatively at a carbon contact (special carbon black) into carbon monoxide (Boudouard equilibrium). Usually, water is set free during the reaction of NaOH with an acidic medium. The analysis duration (depending on sample composition and sample weight) was from 8 to 15 min. Samples were always run in duplicate to ensure repeatability.

#### UV visible spectrophotometry

UV–visible spectroscopic analysis of the crude and upgraded oil samples was carried out using a JASCO V-730 double-beam spectrophotometer (cuvette width of 1 cm) at a wavelength scanned from 200–500 nm. All samples were diluted in petroleum ether before analysis. Samples were prepared with different concentrations to explore the possible concentration dependence of the spectral shape and to make sure that the absorbance is in the linear range of the Lambert-Beer law (the maximum allowed absorbance in this work is 1). The absorbance is related to the concentration of the absorbing species through Eq. 1, where A is the absorbance, ε is the mass absorptivity, l is the path length (1 cm), and c is the sample concentration.

1 $$A=\varepsilon\mathrm{lc}$$

#### Adsorption experiments

Adsorption experiments were conducted in batch mode in a 500 mL Erlenmeyer flask containing 20 mL of crude oil, 20 g of adsorbent, and 100 mL of petroleum ether with a magnetic stirrer. The Erlenmeyer flask was covered with aluminium foil during the entire course of the experiments to avoid the interaction of light waves. The solution in the system was then drawn out without disturbing the lower solid phase. Millipore filtration (with a fritted glass filter with a membrane with a pore size of 0.45 µm) was used for the filtration and was carried out to remove any particles or sediment that settled in the top funnel portion. The process was repeated for all the adsorbents. The collected homogeneous fraction from all the samples was collected and stored for analysis.

The percentage removal implies the quantity of oxygenates removed from the crude biogenic oil. The equation used to describe the percentage removal is shown in Eq. 2.

2 $$\text{Precentage removal}= \frac{{\mathrm{C}}_{0}-\mathrm{C}}{{\mathrm{C}}_{0}}\times 100$$

where C_0_ is the initial concentration of the crude oil in g/L and C is the upgraded oil concentration in g/L.

#### GC–MS spectrometry

The liquid products were analysed by a Trace 1300 gas chromatograph with a single quadrupole mass spectrometer (Thermo Scientific) using a TGS QC-Thermo column (15 m × 250 µm × 0.25 µm). Samples were diluted in n-pentane (HPLC grade) before the GC–MS analysis. The method used was as follows: helium flow rate of 1 mL/min, injection volume of 1µL, injection port temperature of 280 °C, split ratio of 30:1, solvent delay of 60 s, initial oven temperature of 60 °C for 10 min, ramped at 3 °C/min for 5 min, then ramped at 20 °C/min for 1 min. The total run time was around 30 min. The NIST library was used for the peak identification of various compounds in the GC–MS spectrum. The current focus of investigation is to identify the main chemical compounds in crude and refined oil, and trace component analysis is out of the scope of the present investigations.

#### Qualitative 1H NMR analysis

20 mg of oil was diluted with 0.6 mL of CDCl_3_. ^1^H NMR spectra were recorded on a Bruker Advance 500 MHz instrument at 25 °C using a normal broadband probe head. Samples were prepared in a 5 mm NMR tube in CDCl_3_ as solvent. Chemical shifts are referenced to the residual solvent peak of CDCl_3_ at 7.26 ppm. The probe temperature was measured using a Pt-100 resistance thermometer and adjusted using a Bruker Eurotherm variable temperature controller. NMR data were processed with Mestre Nova software. The integrals give information about the relative number of hydrogens under the signals and relative to the first integrated signal (intensity is unity).

#### Cleanliness and compatibility by spot test

Samples were mixed with n-hexane, and an aliquot was taken from the surface of the sample for a spot test. In this method, one drop of the sample was plunged on a Whatman #2 qualitative filter paper and heated in a hot air oven (Model: Heraeus D 6450) at 100 °C for 1, 30, 60 min and 20, 24 h. The obtained spots were carefully examined to see evidence of suspended solids. The spots were then compared to the reference spot shown in Table 4.

**Table 4:** Reference spot description (Adapted from ASTM D4740-20)

Spot no.	Characterization of the spots
1	Homogeneous spot (no inner ring formation)
2	Faint or poorly defined inner ring
3	Well-defined thin inner ring, slightly darker than the background
4	Well-defined inner ring, thicker than the ring in spot no. 3 and darker than the background
5	Very dark, solid, or solid area in the centre. The central area is much darker than the background

#### Oxidation stability

**Fig. 2:**
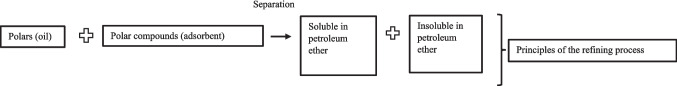
Principle of separation process

**Fig. 3:**
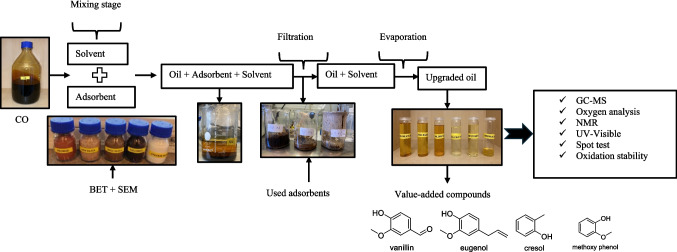
Scheme of the methodology used in the present study (sequential steps in the investigations)

The oxidation stability of the samples was carried out as per ASTM D 2274. 2 mL of the sample was placed in a beaker and heated at 110 °C in a hot air oven (Model: Heraeus D 6450) for 24 h to inspect the stability of crude and upgraded oil samples. The scheme of the principle and methodology used in the present study is shown in Figs. 2 and 3.

## Results and discussions

### Nature of adsorbents used in the separation strategy

**Fig. 4:**
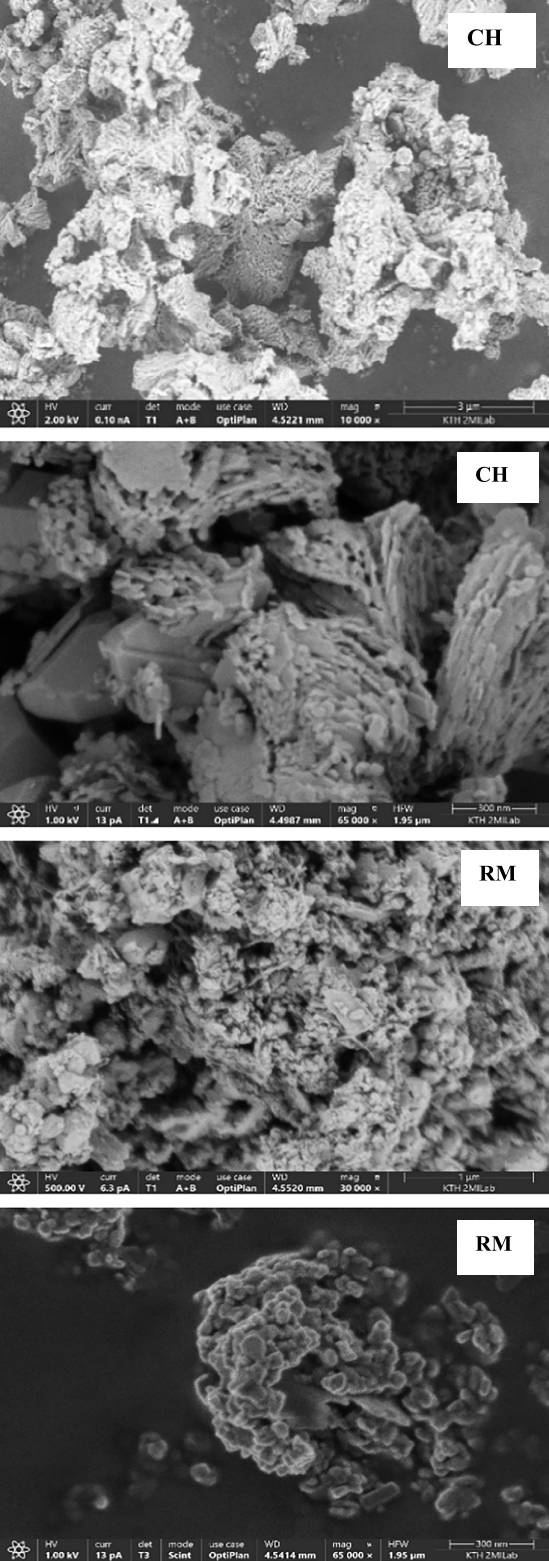

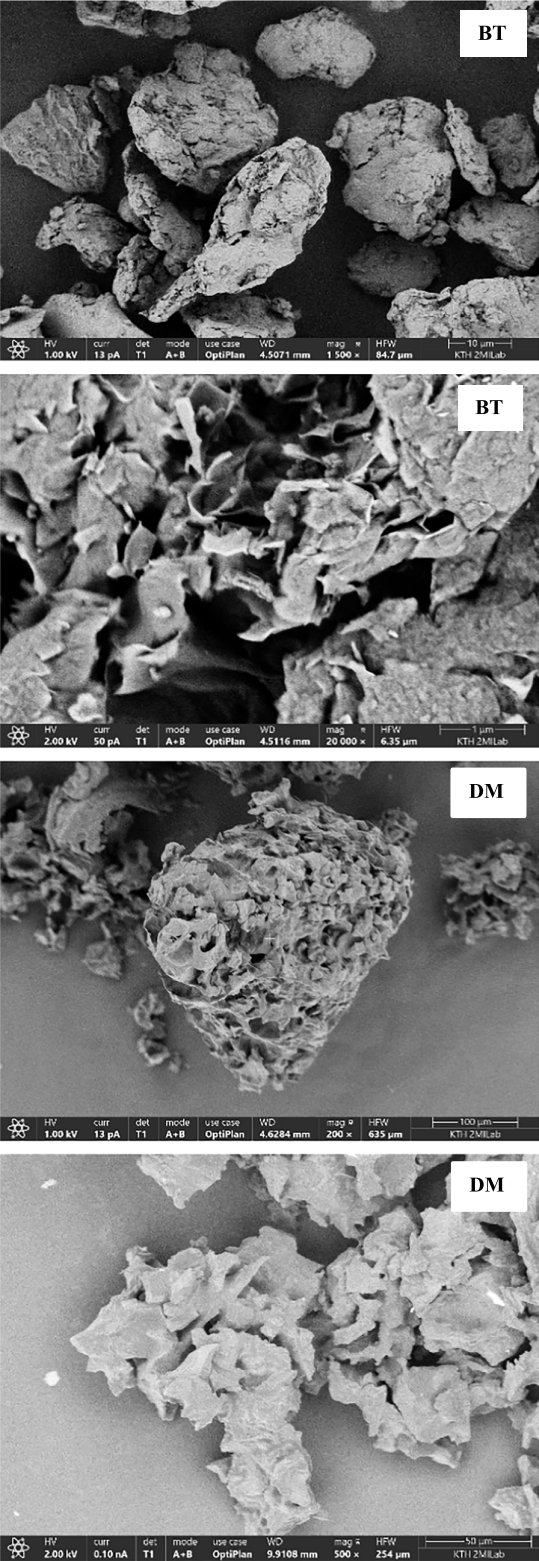

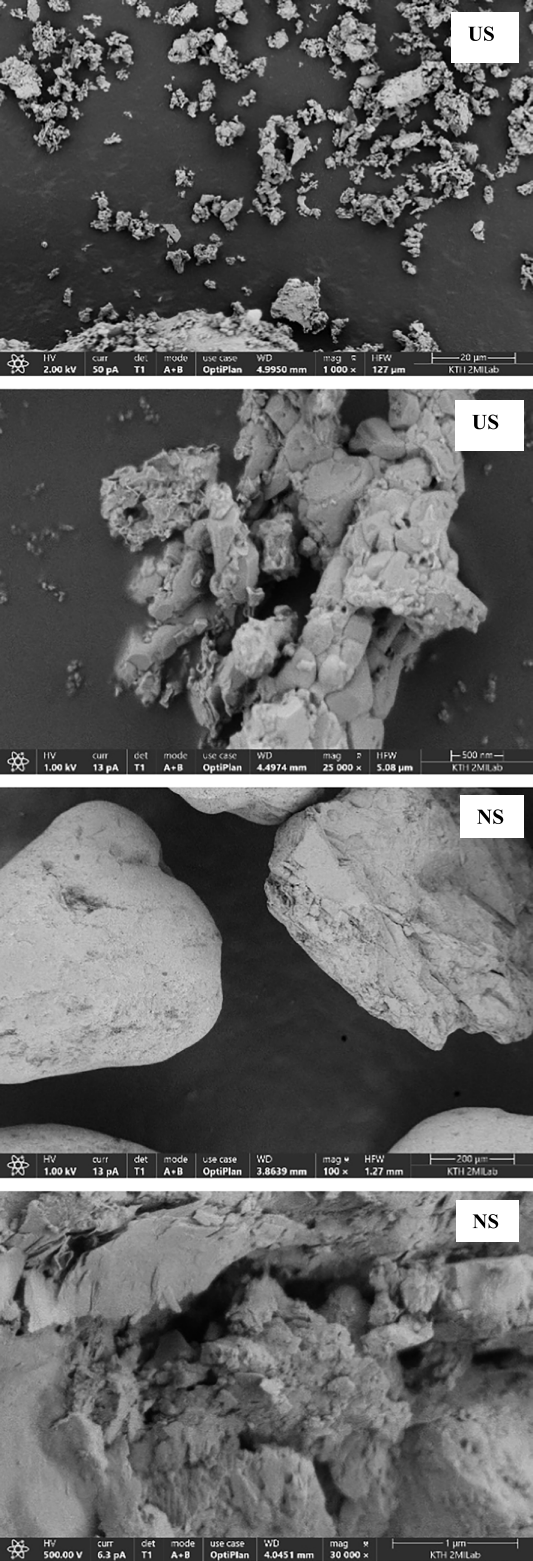
SEM micrograph of various adsorbents

The morphology of various adsorbents was taken at various magnifications to understand the porous nature of the samples. The micrograph showing the morphology of various adsorbents was examined at low and high magnifications (Fig. 4). The SEM micrograph showed that the surface porosity of the calcium hydroxide and bentonite is, in relative terms, higher compared to other adsorbents. The reduction in the acceleration voltage of the electron beam during SEM analysis could identify the micropores, mesopores, and macropores on the surface of calcium hydroxide compared to the other adsorbents. EDAX imaging using Chemi SEM can identify the lateral distribution of various organic and inorganic metallic elements in the sample. The quant map and EDAX mapping of a typical sample location are shown in Figure S1. The results showed that the sample consisted of mainly inorganic elements (Table 5). The presence of potassium elements in the silica-based samples (NS and US) may be from the pyrolysis of biomass materials using silica feedstock in BTG. The potassium content in the used silica is higher than that in new silica. Interestingly, it can be noted that the high amount of potassium is in the used silica compared to the fresh silica. The used silica is obtained from the pyrolysis of biomass using fresh silica as bed material in Meva Energy Sweden. The potassium is relatively higher in the used silica due to the leaching of potassium from the biomass during thermal pyrolysis. The confirmation of these elements was carried out by imaging the samples in various locations. The solid-like thick deposits formed in calcium hydroxide after the separation experiments could be due to the reaction of oxygenates in the solid phase that may react with the metals to form metallic oxides. The photo of the solid formation is shown in Figure S4. The BET surface area, total pore area, and total pore volume of various adsorbents are shown in Table 5. The results from the SEM micrograph, supported by surface area characterization using BET, revealed that the surface area of the calcium hydroxide was significantly higher than that of the other adsorbents due to higher porosity. The N_2_ adsorption–desorption isotherm of various adsorbents to understand the porous nature of the adsorbents is shown in Figure S2. As per the IUPAC classification of the nitrogen adsorption and desorption isotherms, the adsorbents showed similar physisorption isotherms of category IV. Type IV isotherms are characterized by macropores with pore width in a range of 2–50 nm (Thommes et al. 2015; Pulungan et al. 2023).

**Table 5:** Surface characterization of various adsorbents

Adsorbents	BET^#^ (m^2^/g)	Total area in pores (m^2^/g)	Pore width^*^ (A^o^)	Metallic elements
CH	86.45	161.07	7.85	O, Mg, Ca, Al
RM	18.41	10.00	6.21	O, Mg, Ca, Na, Al, Si, Ti, Fe
BT	43.29	36.96	6.28	O, Mg, Ca, Ti, Fe, Al, Si
DM	0.60	1.94	8.31	O, Mg, Ca, Al, P, S, K
US	0.15	0.14	6.28	O, Mg, Ca, Al, Si, K
NS	0.11	0.11	9.49	O, Mg, Ca, K, Al, Si, Fe

^#^Brunauer–Emmett–Teller surface area analysis through gas adsorption, *calculated by the Horwath

### Elemental analysis of crude and upgraded oil samples

The oxygen content in crude and upgraded oil samples was measured three times, and the average values are reported with an error of ± 0.1. The main challenge with crude biogenic oil is the high quantities of carbonyl and carboxyl groups, which cause low heating value and instability of UO-CO (Paiva et al. 2023). The preliminary investigation showed that all adsorbents can contribute to further deoxygenation of the crude biogenic oil through phase separation. It is interesting to note that the oxygen content in UO-CH is reduced by 99.65 wt. % compared to crude biogenic oil. The significant reduction of oxygen with calcium hydroxide might be due to the high specific surface area of calcium hydroxide compared to other adsorbents. However, as will be shown below, the fraction of crude oil constituents was also significantly reduced in this system. The addition of the adsorbents and solvent (petroleum ether) into crude oil causes the separation of the compounds into the soluble and insoluble fractions. This is due to the polarity differences between the adsorbents and crude biogenic oil. This causes the separation of the water-insoluble fraction and deposits at the bottom of the storage container (polar dissolves in polar and non-polar dissolves in non-polar) and induces phase separation.

**Fig. 5:**
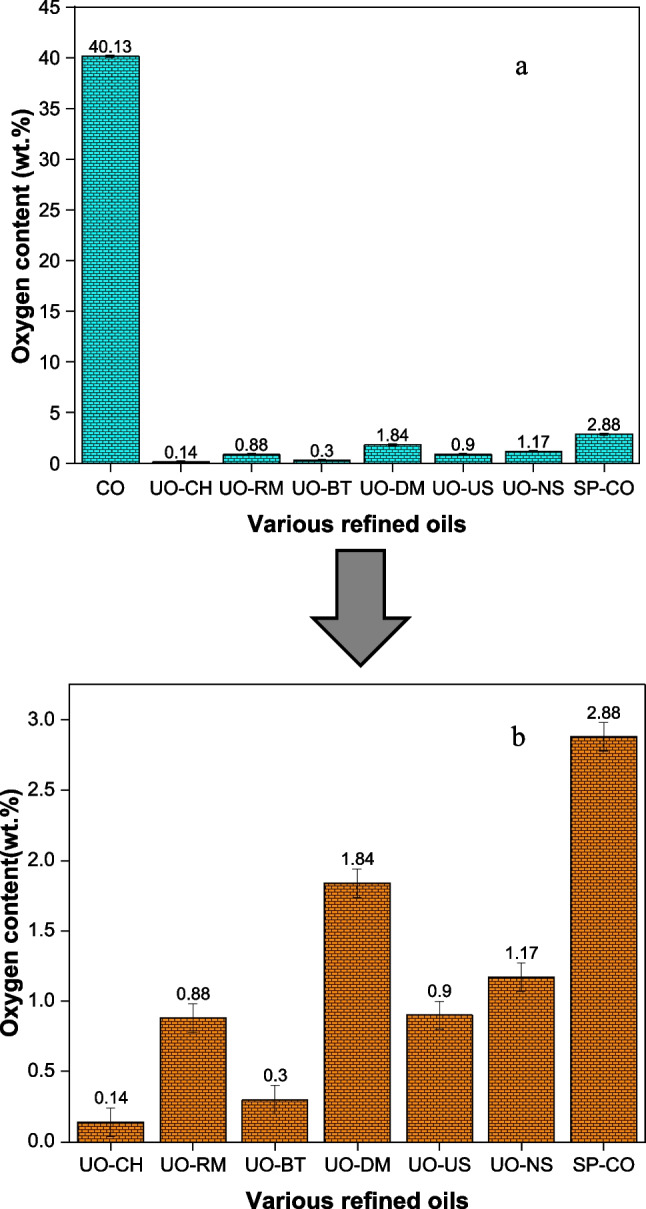
Oxygen content analysis. **a** Comparison of crude and upgraded oils. **b** Expanded view of upgraded oil samples

When mixing the crude oil with petroleum ether, we observed phase separation. This shows that there was a crude oil fraction with low solubility in petroleum ether. Most probably, this was a more polar fraction. It can be interesting to point out that adsorption on oxide surfaces also favours the removal of polar constituents. Whether adsorbents induce phase separation or not is difficult to say and needs further investigation. The adsorbents themselves constitute a separate phase, and they bring anything adsorbed to them. Figure 5 represents the oxygen content analysis of crude biogenic oil and upgraded oils using various adsorbents.

### Overview of the upgrading strategy

The obtained upgraded oil appeared to be bright yellow with a pleasant odour (homogeneous soluble upper phase) compared to the crude biogenic pyrolysis oil. The solid fraction looked like a darker solid settled at the bottom of the beaker after the refining process, and the solid fractions were stored for future investigations (bottom phase). The upgraded oil obtained from various adsorbents was defined as upgraded oil, followed by the adsorbent name, which is represented in the following format in the entire manuscript: (CO for the upgraded oil from crude biogenic oil, UO-CH for the upgraded oil from calcium hydroxide, UO-RM for the upgraded oil from red mud, UO-BT for the upgraded oil from bentonite, UO-DM for the upgraded oil from dolomite, UO-US for the upgraded oil from used silica, and SP-CO for the homogeneous upper phase). The current research concentrates mainly on upgrading oil fractions to value-added chemicals. The solid fraction was not investigated in the present study.

The study was also performed to understand the effect of solvent (petroleum ether) on the upgrading of crude biogenic oil. It is interesting to note that when 20 mL of crude oil is diluted in 100 mL of petroleum ether, the upper homogeneous petroleum ether phase has a crude oil concentration corresponding to 50% of the total system concentration. In other words, 50% of the oil constituents phase separate from the mixture. As will be shown later, the phase separation is highly selective when looking at the oxygen content. The results showed that oxygenates were reduced by 92.82 wt. % in the homogeneous petroleum ether phase compared to the crude biogenic oil. Table 6 shows the correlation of various adsorbents, surface area, and percentage oxygen removal.

**Table 6:** Correlation of various adsorbents, surface area, and percentage oxygen removal

Adsorbents	BET surface area (m^2^/g)	Oxygen removal with reference soluble phase of crude oil (%)
CH	161.07	99.68
RM	10	79.61
BT	36.96	98.04
DM	1.94	55.81
US	0.14	79.67
NS	0.11	75.50

**Fig. 6:**
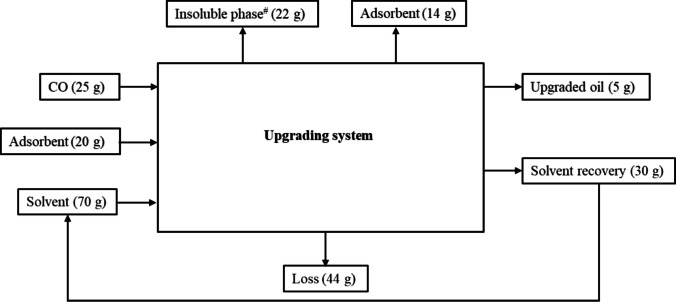
Mass balance of batch-scale upgrading system (.^#^Insoluble phase comprises adsorbed oil constituents, adsorbent, and solvent)

Figure 6 illustrates the mass balance (including input oil, recovered fractions, solvent recovery, and losses) of the upgrading system. 20 mL of crude oil is mixed in 100 mL of petroleum ether, and 20 g adsorbent results in a yield of 5 g upgraded oil (20 wt.%) with a solvent recovery of 30 g (43 wt.%). The insoluble phase of 22 g consists of a solid mixture of oil, solvent, and adsorbent. The losses are mainly caused by the adsorption of oil by the insolubles, the millipore filtration, and the rotary evaporation. It would be difficult to estimate the exact losses at each stage due to the mixture of oil, adsorbent, and solvent in the batch-scale process. Thus, we were not able to quantify losses at each stage. However, we have found a total loss of 38 wt.% ± 5. Yes, losses are consistent across all experiments with a margin of 5 wt.%.

Crude oil used in this work was not completely miscible with petroleum ether. After stirring the mixture for 6 h and allowing it to settle, phase separation occurred. The elemental analysis revealed that the oxygen content in the crude oil present in the homogeneous phase was significantly lower than in the original material. The oxygen content was reduced from 40.13% in the crude oil to 2.88% in the crude oil constituents present in the homogeneous phase. It is quite clear that the separation in terms of oxygen content was far more efficient than the general separation in terms of crude oil constituents.

As can be seen in Table 7, the overall removal of crude oil constituents in the presence of absorbents varied between 65 and 97% compared to 50% in the absence of absorbents. The systems with the highest and second-highest removal ratios correspond to the adsorbents with the largest and second-largest specific surface areas. Hence, the removal efficiency could not be attributed to any other material properties than the specific surface area. The oxygen removal attributed only to the adsorbents varied between 56 and 99.7%. Again, the adsorbents with the highest removal ratios were also the ones with the largest specific surface areas. Indeed, a high oxygen removal ratio indicates a highly upgraded product. However, since both the oxygen removal and the overall crude oil constituent removal seem to mainly depend on the adsorbent surface area to solution volume ratio, other ways of comparing the systems are needed. From a purely economic point of view, a system where primarily the oxygen content was removed from the crude oil would be preferred. In other words, the ratio between oxygen removal and crude oil constituent removal should be as high as possible. In data containing columns five and six in Table 7, this ratio has been calculated for both crude oil and the homogeneous petroleum ether phase as references. It is interesting to note that the systems displaying the highest ratios were not the ones for which the specific surface areas of the adsorbents were the largest. This could be attributed to the presence of various types of metallic oxides in the red mud, dolomite, which may enhance the selective adsorption of oxygen compared to calcium hydroxide. This implies that it might be more beneficial to use RM, DM, US, or NS in a multi-stage process rather than using CH or BT.

**Table 7:** Oil constituent and oxygen content removal rates in percentage for the systems studied in this work

Various systems	Oil removal (relative to SP-CO)	Oil removal (relative to CO)	Oxygen removal (relative to SP-CO)	Oxygen removal (relative to CO)	Selectivity oxygen/oil from CO	Selectivity oxygen/oil adsorbed
CO	–	–	–	–	–	–
SP-CO	–	50	–	96.41	1.93	–
CH	93.4	96.7	99.68	99.99	1.03	1.07
RM	33.28	66.64	79.61	99.27	1.49	2.39
BT	81.16	90.58	98.04	99.93	1.10	1.21
DM	30.83	65.42	55.81	98.41	1.50	1.81
US	34.93	67.46	79.67	99.27	1.47	2.28
NS	39.69	69.84	75.50	99.12	1.42	1.90

Upon adding the solid adsorbents to the mixture of crude oil and petroleum ether, the crude oil constituent content, as well as the oxygen content in the homogeneous petroleum ether phase, was further reduced. The removal of crude oil constituents in comparison to the composition of the homogeneous petroleum ether phase obtained after the initial phase separation is 93.40, 33.28, 81.16, 30.83, 34.93, and 39.69% for UO-CH, UO-RM, UO-BT, UO-DM, UO-US, and UO-NS, respectively. This reveals a significant difference between the adsorbents. However, considering the specific surface areas of the adsorbents, it is evident that the adsorbents with the highest specific surface areas (CH and BT) were responsible for the highest removals. The oxygen content of the crude oil constituents present in the homogeneous petroleum ether phase after exposure to the adsorbents was determined to be 0.14, 0.88, 0.30, 1.84, 0.90, and 1.17% for CH, RM, BT, DM, US, and NS, respectively. Given the fact that the oxygen content of the crude oil present in the homogeneous petroleum ether phase in the absence of adsorbent was 2.88%, this clearly shows that all adsorbents displayed selectivity for removing oxygen content compared to the general removal of crude oil constituents. Once again, the highest selectivity for removing oxygen content was observed for the adsorbents with the largest specific surface areas (CH and BT). To enable a comparison of the efficiency of the different separation methods, both with respect to overall crude oil constituent removal and the removal of oxygen content, Table 7 is generated from the experimental data. In this table, the first data column includes the overall crude oil constituent removal compared to the homogeneous petroleum ether phase in the absence of adsorbent. The second data column summarizes the overall crude oil constituent removal from the system as compared to the originally added crude oil. In other words, the first data column displays the efficiency of the adsorbents, while the second data column displays the removal efficiency of the whole system (phase separation and adsorbent). The third data column displays the removal of oxygen content compared to the homogeneous petroleum ether phase in the absence of adsorbent. The percentage oxygen removal is calculated as:

3 $$\text{Percentage oxygen removal}=100\times \left(1-\frac{{\mathrm{m}}_{\mathrm{ox}-\mathrm{sample}}}{{\mathrm{m}}_{\mathrm{ox}-\mathrm{ref}}}\right)$$

where *m*_ox-sample_ is calculated as the mass of oxygen content remaining in the homogeneous phase (i.e., by multiplying the total oil constituent mass in the homogeneous phase with the oxygen content given above). The fourth data column displays the removal of oxygen content compared to the crude oil (calculated in the same way but using crude oil as the reference).

### Chemical composition of the crude and upgraded oil samples

GC–MS was carried out to unravel the chemical composition and identification of various classes of compounds in the crude and upgraded oil fractions. GC-chromatograms obtained from crude and upgraded oil fractions are shown in the supplementary information (Figure S3). The compounds were identified using the MS library database, and the results are shown in Table 8. To verify the identification, retention indices or analysis of reference substances could be performed, but this is outside the scope of the present study. We have conducted the GC–MS analysis twice to strengthen the determination of the composition of the crude and upgraded oil fractions. The match algorithm is a complex probability factor based on the differences between the forward factors of all the candidates in the GC chromatogram. The peaks with higher match factors (≥ 700) with respect to the NIST library are reported in the table. Phenolic compounds were the most prevalent compounds in upgraded oil using various adsorbents compared to crude oil (CO). The main representative compounds, such as 2-cyclopenten-1-one, 2-methyl, 2-furan carboxaldehyde, 5-methyl, phenolic groups, cresol, 3,4-dimethoxy toluene, eugenol, vanillin, and homovanillic acid, were the dominant compounds in the crude biogenic pyrolysis oil. The aldehyde, ketone, and olefin groups in the crude oil and soluble phase were reduced using various adsorbents. Similar findings on the enrichment of phenolic compounds from the aqueous fraction of biomass-derived pyrolysis are found in the literature (Paiva et al. 2023; Abinisa et al. 2014). Pires et al. (2023) found that the high fraction of phenolic compounds from GC–MS analysis of BTG oil samples could be due to the high lignin content in the feedstock. It is also interesting to note that the presence of vanillin in the crude biogenic pyrolysis oil obtained from BTG is due to the cleavage of β-O-4 linkage in lignin (Seca et al. 1998; Zakzeski et al. 2010).

**Table 8:** Compounds identified from GC–MS analysis of crude and various upgraded oils (NIST match factors in brackets)

Samples	Acids	Aldehydes	Aromatics	Ketones
CO	Homovanillic acid (994)	2-furancarboxaldehyde,5-methyl- (880)	Phenol, 2-methyl- (879) Phenol, 2-methoxy- (943) Phenol, 2,4-dimethyl- (907) Creosol (852) 3,4-dimethoxyltoluene (803) Phenol,4-ethyl-2-methoxy (929) Eugenol (917) Phenol,2-methoxy-4-propyl- (896) Vanillin (833) Phenol,2-methoxy-4-(1-propenyl) (885)	2-cyclopenten-1-one,2-methyl (889) 1,2-cyclopentanedione, 2-hydroxy-3-methyl- (908) 2-cyclopenten-1-one, 2,3-dimethyl- (812) Ethanone,1-(4-hydroxy-3-methoxyphenyl) (804)
UO-CH			Phenol, 2-methoxy- (913) Creosol (tentative) 3,4-dimethoxyltoluene (tentative) Phenol,4-ethyl-2-methoxy (933) Eugenol (914) Phenol,2-methoxy-4-propyl- (918) Phenol,2-methoxy-4-(1-propenyl) (935)	
UO-RM			Phenol, 2-methoxy- (913) Phenol, 2,4-dimethyl- (846) Creosol (922) 3,4-dimethoxyltoluene (820) Phenol,4-ethyl-2-methoxy (933) Eugenol (914) Phenol,2-methoxy-4-propyl- (918) Phenol,2-methoxy-4-(1-propenyl) (935)	
UO-BT			Phenol, 2-methoxy- (914) Creosol (924) 3,4-dimethoxyltoluene (tentative) Phenol,4-ethyl-2-methoxy (916) Eugenol (907) Phenol,2-methoxy-4-propyl- (891) Phenol,2-methoxy-4-(1-propenyl) (922)	
UO-DM			Phenol, 2-methoxy- (913) Phenol, 2,4-dimethyl- (822) Creosol (926) 3,4-dimethoxyltoluene (818) Phenol,4-ethyl-2-methoxy (921) Eugenol (919) Phenol,2-methoxy-4-propyl- (920) Phenol,2-methoxy-4-(1-propenyl) (911)	
UO-US			Phenol, 2-methoxy- (915) Phenol, 2,4-dimethyl- (837) Creosol (929) 3,4-dimethoxyltoluene (817) Phenol,4-ethyl-2-methoxy (936) Eugenol (911) Phenol,2-methoxy-4-propyl- (923) Vanillin (844) Phenol,2-methoxy-4-(1-propenyl) (914)	
UO-NS			Phenol, 2-methoxy- (911) Phenol, 2,4-dimethyl- (845) Creosol (tentative) 3,4-dimethoxyltoluene (820) Phenol,4-ethyl-2-methoxy (936) Eugenol (898) Phenol,2-methoxy-4-propyl- (922) Phenol,2-methoxy-4-(1-propenyl) (907)	
SP-CO			Phenol, 2-methyl- (844) Phenol, 2-methoxy- (913) Phenol, 2,4-dimethyl- (844) Creosol (933) 3,4-dimethoxyltoluene (820) Phenol,4-ethyl-2-methoxy (929) Eugenol (889) Phenol,2-methoxy-4-propyl- (930) Vanillin (819) Phenol,2-methoxy-4-(1-propenyl-) (914)	1,2-cyclopentanedione,3-methyl- (814)

As seen in Fig. S3, the coelution is most severe in the first 3 min of the chromatogram (see chromatogram of petroleum ether in supplementary information), where the solvent petroleum ether is eluting together with the most volatile analytes in the sample. However, in the later parts of the chromatogram, where the upgraded biogenic oil components elute, the coelution is not as severe a problem. However, we can confirm the aliphatic distribution from the NMR spectra. As we have used petroleum ether as a solvent, this could be due to the interaction of aliphatic hydrocarbons in the petroleum ether with aliphatic constituents in the crude biogenic oil. In future studies, more comprehensive studies are required to identify such interactions. This could be accomplished by the use of two-dimensional gas chromatography and mass spectrometry, utilizing both polar and non-polar columns, separating compounds sequentially depending on polarity and volatility. The use of polar and non-polar columns spatially distributes compounds in two dimensions, which causes better separation with high resolution. The studies conducted using compositional analysis of BTG and Pyrovac oil by Pires et al. 2023 showed oxygenated compounds in the aqueous phase.

### Thermal stability of crude and upgraded oil

**Fig. 7:**
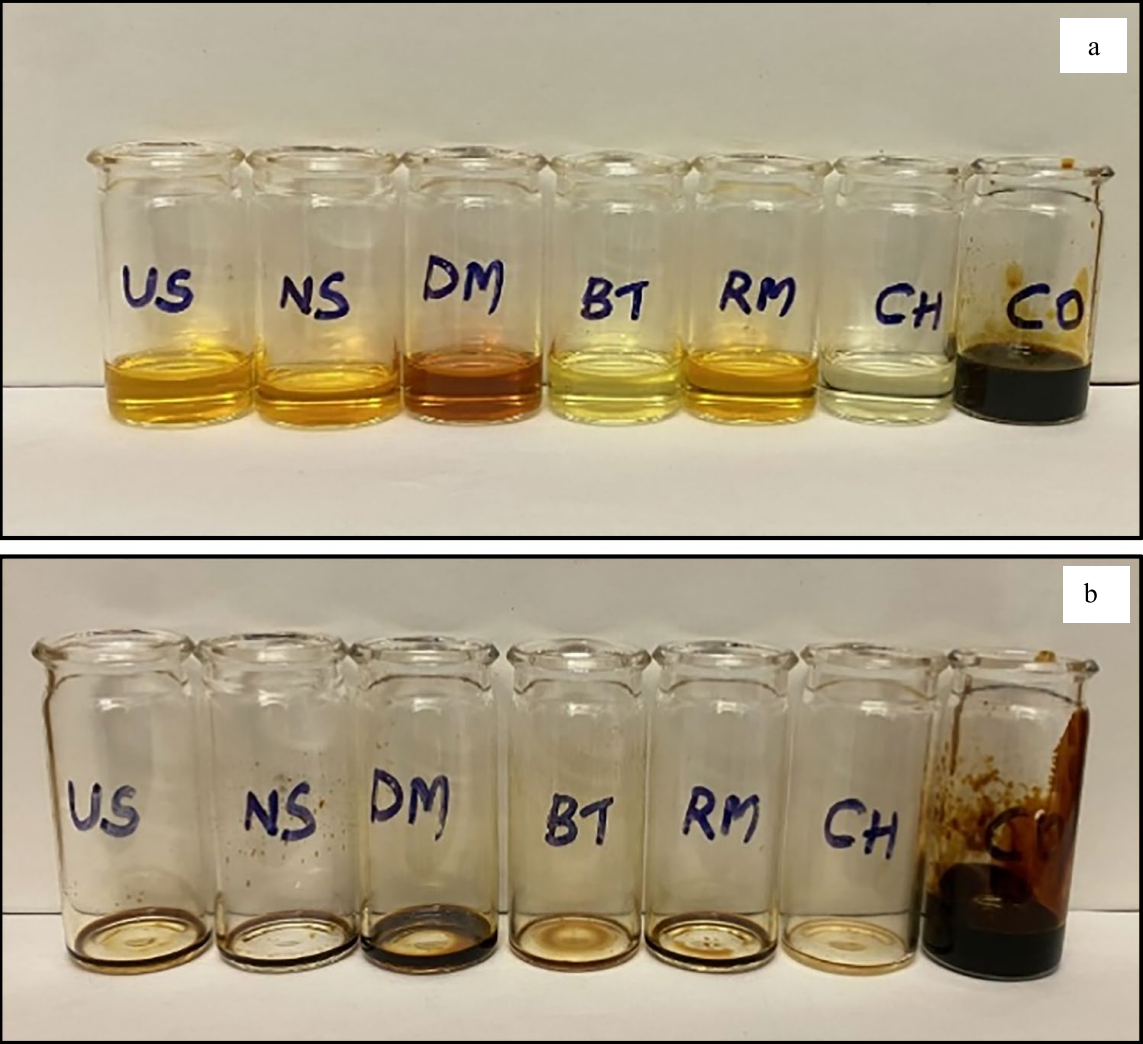
Oxidation stability test of crude and upgraded oil samples. (a. Before heating the samples in a hot air oven, b. after heating the samples in a hot air oven)

The thermal stability of the crude and upgraded oil fractions was investigated using spot and oxidation stability tests. These tests were conducted to understand the stability of oil samples during long-term storage in the industry after the pilot-scale run in the industrial units. Figures S5–11 show the spot test of crude and upgraded oil samples at various times. The spot test suggests the compatibility (absence of suspended solids) and the cleanliness of the oil samples, whereas the oxidation stability test gives an idea about the oxidation capability of the oil samples. The spot formation from the ASTM D4740 was visually examined for evidence of separation and graded for compatibility. The findings showed that the spot formation from the upgraded and crude samples presents a great divergence in terms of compatibility and separation. No evidence of deposit formation was found in the upgraded oil samples compared to the crude. However, the spot formation in the case of calcium hydroxide and bentonite showed a cleaner spot compared to others, which indicates good compatibility and separation over the other adsorbents. The studies performed by Ali et al. 2022 investigated the stability of asphaltenes in crude oil using a spot and deposit test using similar methodologies (Fakher et al. 2019; Kass et al. 2020). The oxidation stability test displayed higher stability in the case of upgraded oil samples compared to the crude oil. The waxy deposits on the surface of the glass vials could be due to the polymerization of olefins in the crude oil (Fig. 7). Spot tests supported by oxidation stability studies suggest that upgraded oil samples from the bentonite and calcium hydroxide were superior to others, which again confirms the better stability of the samples. Similar studies were conducted on the stability analysis of crude tyre pyrolysis oil (Mohan et al. 2021).

With reference to Table 4, the spot formation in CO can be denoted by 5, UO-CH and UO-BT showed no spot formation (1,); UO-RM, UO-US, and UO-NS showed a faint or poorly defined ring (2,); UO-DM showed a well-defined inner ring slightly darker than the background (3). Homogeneous spots in the case of UO-CH and UO-BM showed no gummy deposit formation compared to others (which means higher thermal stability).

### UV–visible spectrophotometric studies of crude and upgraded oils

UV–visible spectrophotometry was used to explore the impact of the different adsorbents on process efficiency by calculating the mass concentration of crude and upgraded oil samples. UV–vis spectrophotometric studies were used to find out the thermal stability, chemical composition, and percentage removal of oxygen-containing compounds. The major difference between UV–visible spectrophotometric studies and other analytical instrumentation is the quantitative estimation of process efficiency. UV spectrophotometric studies are widely used in the understanding of the mass concentration and percentage removal of oxygenated compounds (Cai Lu et al. 2019). A study on UV fluorescence of biogenic pyrolysis oil by Trubetzkaya et al. (2023) showed that aromatic compounds excite at the wavelength of 280 nm due to the presence of aromatic hydrocarbons (Trubetskaya et al. 2023). The crude and upgraded oil was diluted with various dilution ratios to record the absorption spectra of various samples. The upgraded oils from calcium hydroxide, red mud, bentonite, dolomite, used silica, and new silica were diluted with petroleum ether in various ratios given by 1:540, 1:1920, 1:720, 1:2400, 1:1920, and 1:1920, respectively, to record the absorption spectra using UV visible spectrometry and were compared with a diluted mixture of crude oil (1:3000). To be able to use UV–vis spectroscopy to determine concentrations, the absorbance should not significantly exceed 1. As the oil concentration varies by orders of magnitude in the different solutions (depending on which adsorbent was used and if an adsorbent was used), dilution ratios must differ drastically. Beer-Lambert law relates the absorption of light by the solution to the properties of the solution (molar absorptivity, path length, and concentration of absorbing species). The absorbance of the solution can be measured using the variation of absorbance with respect to wavelength. The dilutions were repeated continuously using petroleum ether as a solvent until the maximum absorbance from the UV measurements reached unity as per Beer-Lambert’s law. The removal ratios of oxygen-containing constituents from the upgrading system using calcium hydroxide, red mud, bentonite, dolomite, used silica, and new silica are given by 93.4, 33.3, 81.2, 30.8, 34.9, and 39.7%, respectively. We could reach reasonable absorbances (< 1) through a series of dilutions. The absorbance spectra from the UV spectrometric studies showed that the absorbance occurred in the wavelength range of 250–300 nm. Absorbance in this specific wavelength range is often attributed to aromatic compounds and etherified hydroxyl groups (Ana et al. 1998; Bartolomei et al. 2020). The crude oil constituent concentration in the homogeneous petroleum ether phase is determined from the absorbance at the wavelength 258.8 nm (λ_max_).

The percentage removal of crude oil constituents after adding adsorbents compared to the homogeneous petroleum ether phase obtained in the absence of adsorbents was determined using UV spectrophotometry. The measurements showed that the upgraded oil from the calcium hydroxide and bentonite displayed the highest removal rates. The impact of sample filtration was also explored. The difference in measured concentration between filtered and unfiltered samples was lower than 3% and can be considered insignificant.

### Effect of separation strategy on the quality of oil fractions and nature of adsorbents

The separation of the chemical constituents in the crude oil during the upgrading process could be explained by the polarity of the mixtures and phase separation. The polar compounds in the crude biogenic oil interact with polar compounds in the adsorbents. Crude biogenic pyrolysis oil consists of high oxygenated fractions (ca. 40 wt.%) bound with other classes of compounds such as polyaromatic hydrocarbons, aliphatics, and olefins. Oxygen content analysis showed a significant reduction in the oxygenates in the upgraded oil with the use of various adsorbents compared to the homogeneous soluble upper homogeneous phase (2.88 wt.%). Calcium hydroxide was found to be the best adsorbent (99.65% oxygen removal) compared to other adsorbents. The high separation efficiency of the calcium hydroxide could be explained by the reaction of acidic functionalities in the crude biogenic oil with calcium oxide (base) to form carboxylate and water.

**Fig. 8:**
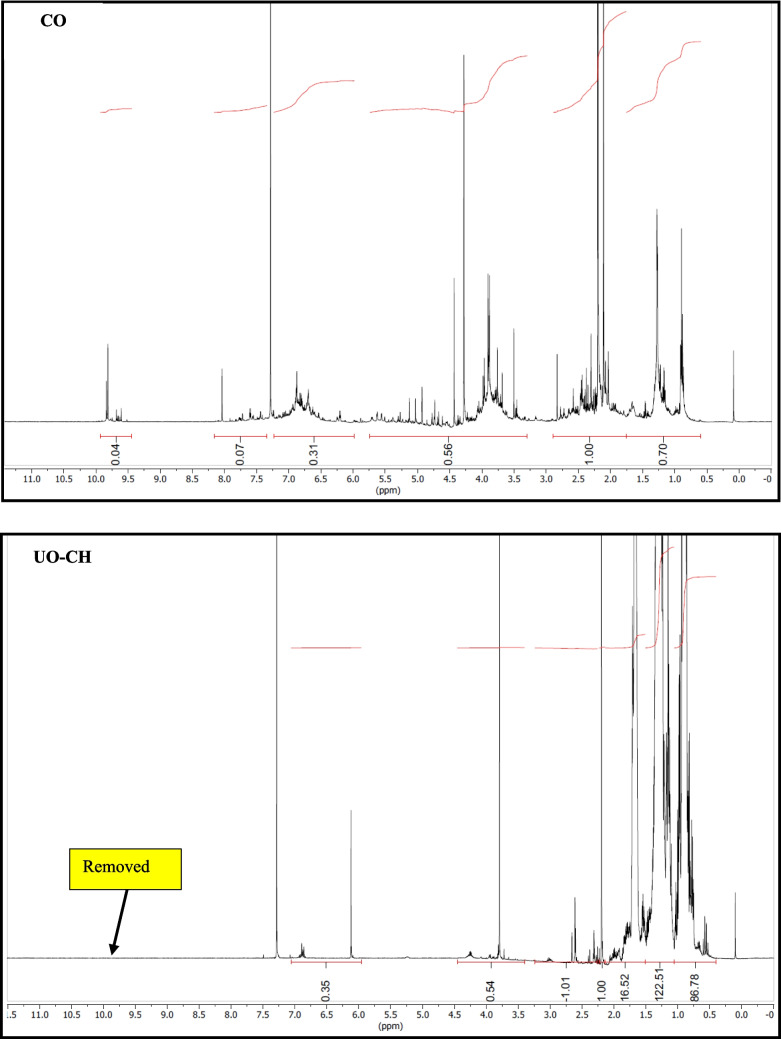

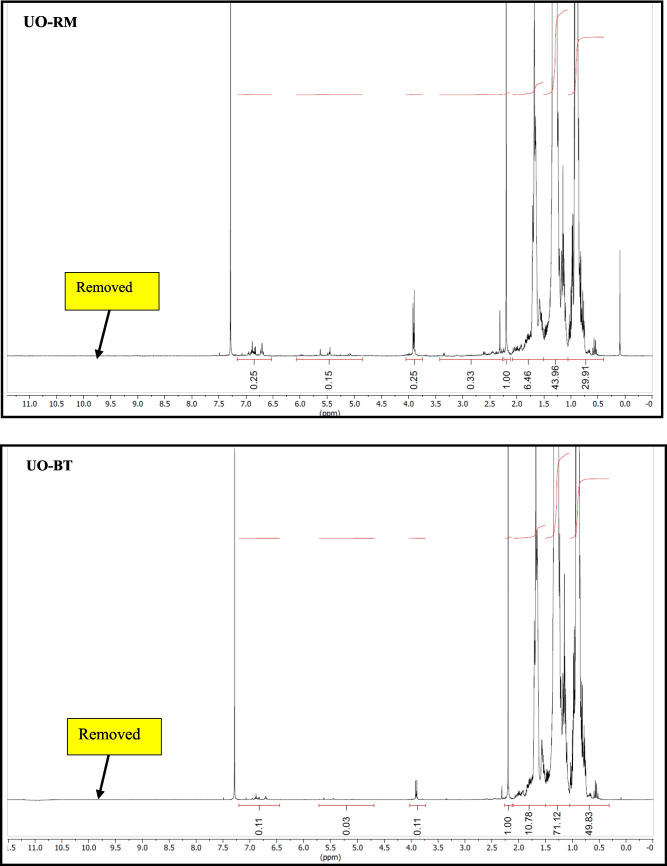

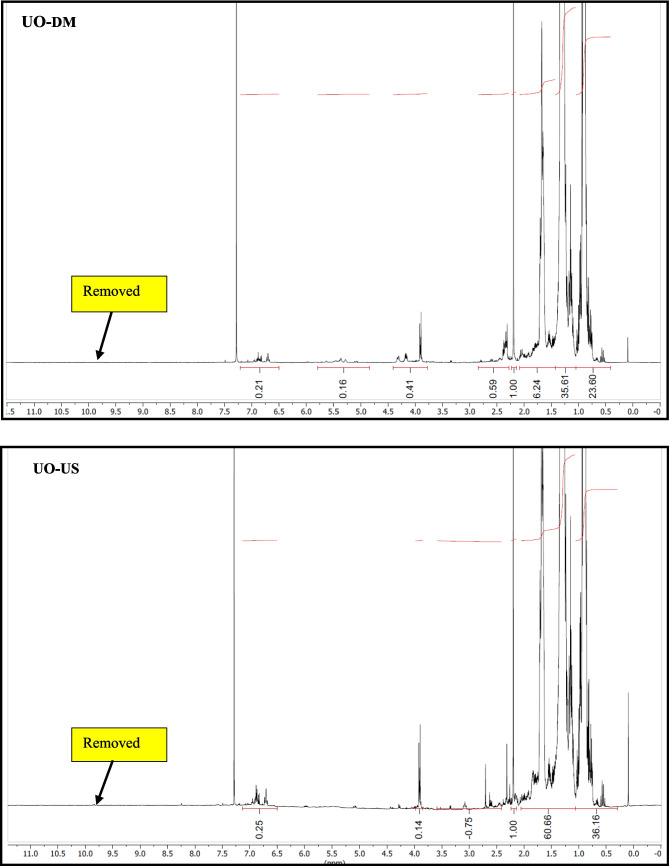

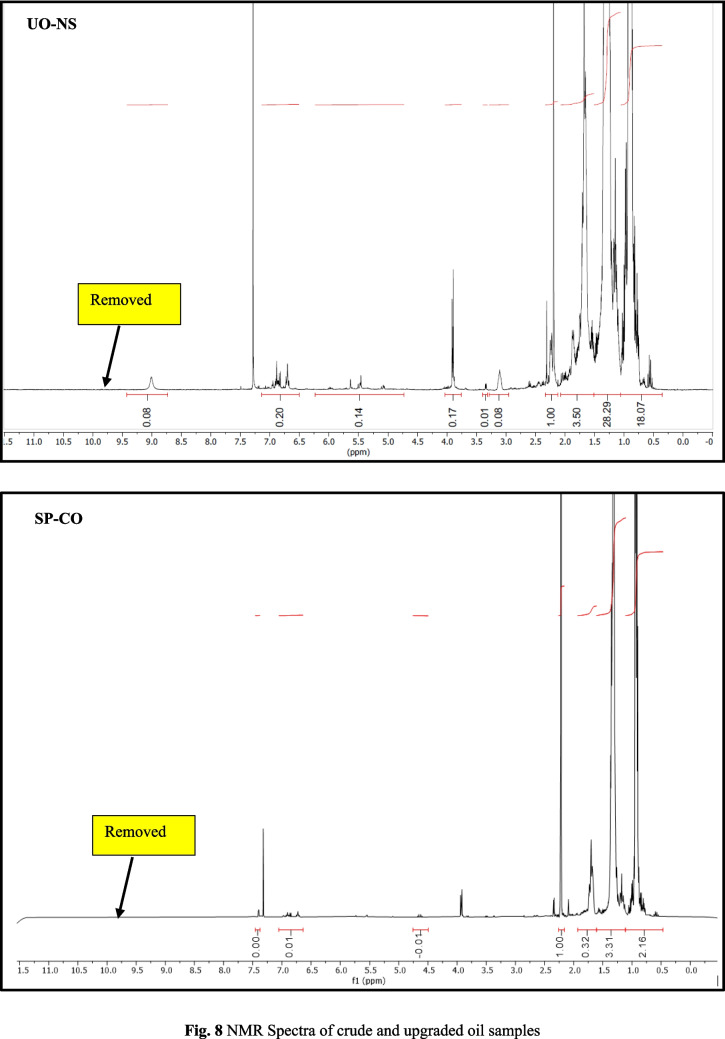
NMR Spectra of crude and upgraded oil samples

NMR supported with GC–MS showed a significant reduction in the aldehydes, ketones, carboxylic acid, and olefins compared to the crude biogenic oil (Fig. 8). This can be corroborated by the reduction in oxygen content in elemental analysis. It is interesting to note that the aliphatics distribution was visible in the NMR spectra, while a few aliphatic compounds were detected with GC–MS. This could be explained by the overlapping of peaks due to the coelution of compounds with similar boiling points. A spot test in combination with the oxidation stability test showed that the upgraded oil was superior in terms of thermal stability in comparison with crude and soluble phases in petroleum ether solvent. The gummy deposits stuck on the walls of the storage beaker containing crude biogenic oil after the oxidation stability test may be due to the polymerization of oxygen-containing constituents. It may be due to the oxygen-containing functionalities (aldehydes, ketones, and carboxylic acids). It is interesting to note that no such deposits could be visualized in the case of upgraded oil fractions.

In a similar study aimed at upgrading polypropylene pyrolysis oil using rice husk-derived silica and petroleum ether, with a specific focus on sulphur removal. results showed a tremendous reduction in sulphur by 89.5% due to the adsorption by the rice husk-derived silica (Kailas et al. 2025). Mora and co-authors (2023) demonstrated a novel dialysis (concentration gradient) and column chromatographic technique to separate value-added phenolic compounds and acid-rich fractions from the wood-based biogenic pyrolysis oil. The study used dialysis of crude biogenic pyrolysis oil with water, methanol, and acetone. The dialyzed liquid was then passed through a chromatographic column filled with amberlite™ XAD7 resin. NaOH was used as a diluent to elute phenolic compounds from the aqueous dialyzed liquid. Naidu et al. separated phenolic compounds from pyrolysis oil from groundnut shells using hexane, ethyl acetate, toluene, and chloroform. They showed that hexane was better in terms of separation of various chemical groups (aromatics, ketones, and phenols) due to a low polarity index and affinity mechanism of polar and non-polar compounds in the solvent (Naidu et al. 2025). Similarly, our studies used petroleum ether as a solvent (low polarity), which is the reason for the separation of phenolics and aromatics.

**Fig. 9:**
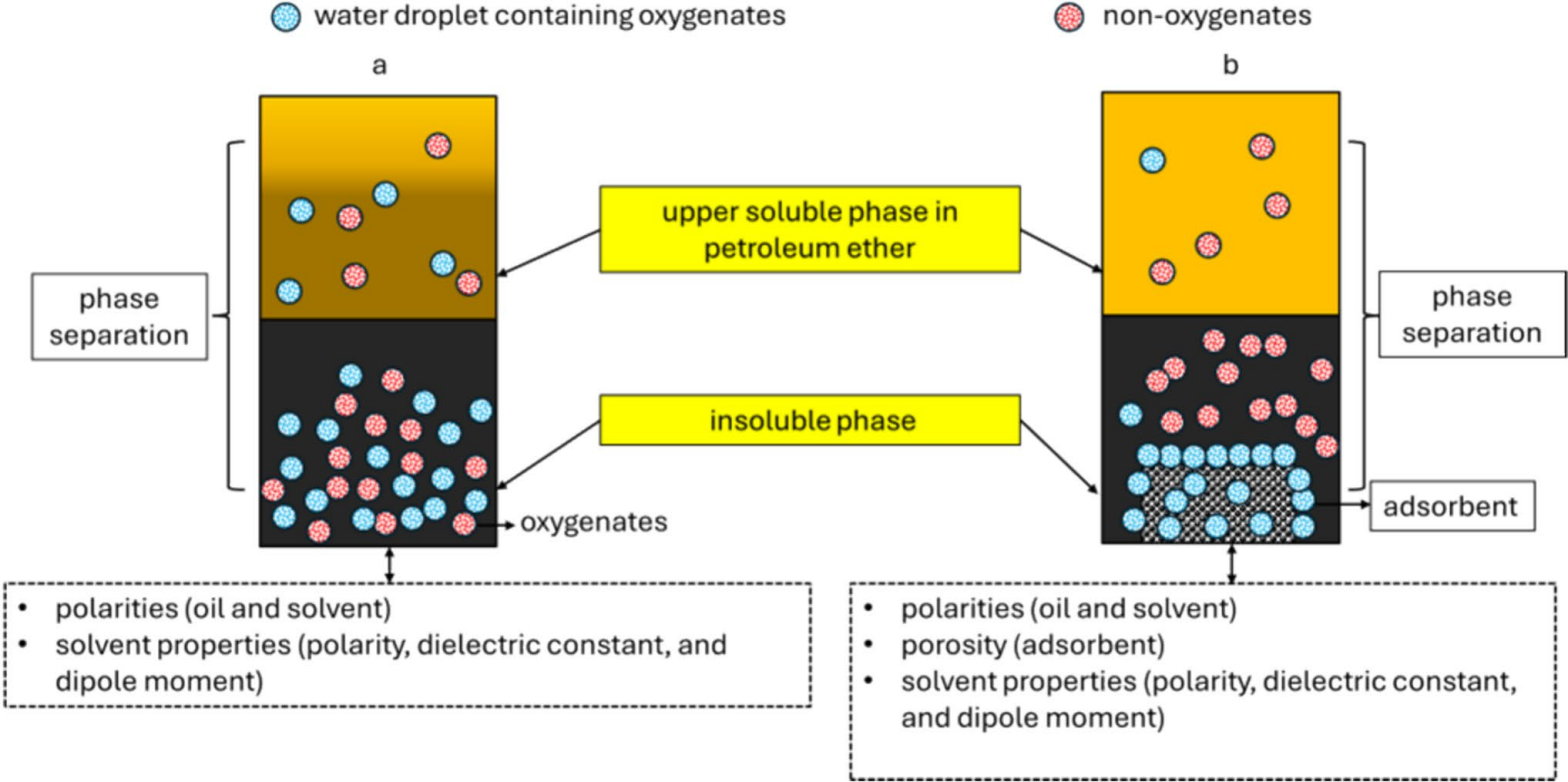
Conceptual schematic of phase separation mechanisms **a** with solvent and **b** with solvent and adsorbents

Compared to previously published literature, the present study used waste adsorbents (acidic, basic, or neutral) and a non-polar diluent (petroleum ether) for upgrading CO. As illustrated in Fig. 9, the addition of solvent results in the separation of the compounds based on the solvent properties (density, polarities, dielectric constant, and dipole moment), solvatochromic parameters such as α, β, and Π^*^, solute and adsorbent properties (Naidu et al. 2025). Due to the lower polarity index (0.1) of petroleum ether compared to other solvents in the literature (ethyl acetate, toluene, and chloroform), and the density (0.73 g/L at 20 °C) of petroleum ether, the compounds in CO are separated into soluble compounds in the upper phase and an insoluble lower phase. The non-polar constituents in the CO interact with the non-polar constituents in the solvent, causing a phase separation of compounds. The addition of adsorbent improves the separation due to a combined effect of the porosity of the adsorbent and the solvent properties (Chen et al. 2011; Mohan et al. 2019, 2021; Mora et al. 2023; Naidu et al. 2025). Calcium hydroxide was found to be the superior adsorbent compared to the others. This could be due to the larger surface area of calcium hydroxide compared to the other adsorbents (Table 5).

## Mechanistic insights of phase separation

When mixing the bio-oil with petroleum ether, the system undergoes phase separation (Fig. 9a). This renders a homogeneous petroleum ether phase where 50% of the oil constituents and 96.41% of the oxygen-containing compounds stay in the bio-oil. The bio-oil compounds dissolved in petroleum ether mainly consist of non-oxygen-containing constituents. The phase separation can partly be attributed to the water content of the bio-oil. However, there is no clear phase separation between an aqueous phase and a bio-oil phase, but rather a precipitation. As the oxygenates, in general, are more polar than other oil constituents, they display a significantly higher affinity for the water in emulsified droplets than for the petroleum ether phase and are thereby preferentially removed by extraction from the petroleum ether phase. When adding an adsorbent to the system under stirring, the adsorbent will be in contact with the bio-oil containing emulsified water droplets as well as the petroleum ether phase. The system, consisting of a solvent phase and a bio-oil with emulsified water droplets, is rather complex.

When assessing the impact of the adsorbents (Fig. 9b), it is important to keep in mind that only 3.59% of the oxygen content remains in the petroleum ether phase after the phase separation. Table 6 presents the removal efficiency for different adsorbents at the same adsorbent surface area to solution volume ratio. In general, the adsorbents are solid oxides or hydroxides with a high affinity for water and polar solutes. As can be seen, the adsorbents remove 55.81–99.68% of the remaining oxygen content. At the same time, significant fractions of all oil-constituents are removed by the adsorbents. Preferential adsorption of oxygenates is mainly observed for RM, DM, US, and NS. However, it should be noted that the selectivity for oxygenates is significantly lower than in the initial phase separation. In general, the adsorbents greatly reduce the overall amount of oil in the petroleum ether phase. CH and BT display low selectivity for oxygenates and appear to generally adsorb (or absorb) all oil-constituents. CH is particularly effective in removing all oil-constituent. The overall oil removal efficiency (from the homogeneous petroleum ether phase) of the adsorbents varies between 30.83 and 93.4%. Mechanistic interpretation is complicated by the fact that the adsorbents also adsorb oil-constituents in the bio-oil phase, which is separated from the petroleum ether phase. The bio-oil phase already has a high content of oxygenates, and it is quite possible that the adsorbents become saturated. It is therefore not meaningful to discuss the adsorption mechanism in detail.

## Scale-up challenges of batch scale upgrading system

The main challenges for the scale-up of the current batch-scale system are low product yield and limited mass transfer, with high mass losses during upgrading processes. Due to the multiple upgrading stages, there are losses due to filtration, evaporation, and adsorption in adsorbents. To scale up the batch-scale upgrading system, the following steps are proposed.

## Recommendations for scale-up from a batch scale system to a continuous scale system

The recommendation for scale-up from a batch scale system to a continuous scale system is detailed below.

1. Use of green solvents (dimethyl ether) and waste adsorbents
2. Separating the soluble and insoluble phases using a phase separator
3. The soluble phase is then passed through multiple columns arranged in series, filled with suitable adsorbent in a packed bed configuration (continuous upgrading system)
4. The solvent would be recovered using a rotary evaporator
5. The used adsorbent can be regenerated using ethanol as a solvent
6. Techno-economic analysis of a continuous upgrading system

## Scalability and safety concerns of the used adsorbents and solvent

In our present upgrading strategy, we have used petroleum ether as a solvent due to its nonpolar nature to test the proof-of-concept of phase separation for removing oxygen-containing compounds. The used petroleum ether in the batch scale strategy can be easily recovered through a rotary evaporator (43 wt.% ± 0.3). Table 9 shows a comparison of the safety and hazard statements of petroleum ether and diethyl ether (Joshi et al. 2019). Nevertheless, for the industry scale-up, it is important to use biogenic solvents from fruit waste (cashew apple) to rectify the above challenges.

**Table 9:** Safety and environmental implications of solvents

Chemicals	Safety statements	Hazard statements
Petroleum ether	Safety googles with side protection Use of chemical-resistant gloves and clothing Respiratory protection mask with filters to avoid low-boiling organic compounds	Flammable liquid and vapour Fatal if swallowed and enters the airways May cause skin irritation (high vapour concentrations) May cause drowsiness or dizziness (high vapour concentrations) Toxic to aquatic life with long-lasting effects
Diethyl ether	Safety googles with side protection Use of chemical-resistant gloves and clothing Respiratory protection mask with filters to avoid low-boiling organic compounds	Flammable liquid and vapour Harmful if swallowed May cause drowsiness or dizziness (high vapour concentrations)

The used adsorbents could be easily regenerated through washing with ethanol in a Millipore filtration apparatus. The use of ethanol is recommended due to its polarity, sustainability, low cost, and availability. The polar oxygenates in the adsorbents can be desorbed using a polar-based solvent, which is a key point in regeneration. The regenerated adsorbents would be heated in a hot air oven to remove the residual solvent for the further upgrading process. Furthermore, the regenerated adsorbents in various stages can be screened to understand the composition, morphology, and purity.

## Safety notes

Petroleum ether: Petroleum ether should be handled using safety goggles, suitable clothing, a respiratory mask with a filter, and chemical-resistant gloves.

Disposal of spent adsorbents and solvents: The spent adsorbents and solvents are stored in a sealed solid collection bag/bottle and handed to the chemicals collection authorities at KTH Royal Institute of Technology for disposal at suitable recycling stations.

## Conclusions

The elemental analysis supported by nuclear magnetic resonance spectrometry (^1^H NMR) and gas chromatography-mass spectrometry (GC–MS) showed a significant reduction in the oxygenates (95%) and yielded cleaner aliphatic-rich fractions with separation of value-added compounds. UV spectrophotometric studies supported by elemental analysis, NMR, and GC–MS showed the percentage removal of oxygen-containing compounds from UO-CH, UO-RM, UO-BT, UO-DM, UO-US, and UO-NS to be 93.4, 33.3, 81.2, 30.8, 34.9, and 39.7%, respectively. This can be confirmed with low wax deposition in the upgraded oil in the spot test and oxidation stability test. It can be summarized from the selectivity analysis that the adsorbents (red mud, dolomite, used silica, and new silica) showed better removal of oxygen than calcium hydroxide. The impact of the present investigation is the separation of value-added compounds such as phenols, vanillin, toluene, and guaiacols. The future scope of the present investigations will be the regeneration and recovery of used adsorbents, improving the efficiency of the upgradation system (lowering the loss due to mass transfer), and the use of biogenic solvents can be explored.

## Supplementary Information

Below is the link to the electronic supplementary material.

Supplementary file1 (DOCX 14.0 KB )

## Data Availability

All the data generated or analyzed during the study are included in the article.

## Acronym

BTG: Biomass Technology Group Netherlands
CH: Calcium hydroxide
RM: Red mud
BT: Bentonite
DM: Dolomite
US: Used silica
NS: New silica
CO: Crude biogenic oil from BTG company
SP-CO: The soluble phase obtained from the upgrading crude biogenic oil
UO-CH: Crude biogenic oil was upgraded using calcium hydroxide as an adsorbent.
UO-RM: Crude biogenic oil was upgraded using red mud as an adsorbent.
UO-BT: Crude biogenic oil was upgraded using bentonite as an adsorbent.
UO-DM: Crude biogenic oil was upgraded using dolomite as an adsorbent.
UO-US: Crude biogenic oil upgraded using silica as an adsorbent.
UO-NS: Crude biogenic oil was upgraded using new silica as an adsorbent.
IUPAC: International Union of Pure and Applied Chemistry
GC-MS: Gas Chromatography-Mass Spectrometry
^1^H NMR: Proton Nuclear Magnetic Resonance Spectrometry
EDAX: Energy Dispersive X-ray analysis
SEM: Scanning Electron Microscopy
ASTM: American Society of Testing and Materials
RT: Retention time
BET: Brunauer-Emmett-Teller analysis
STP: Standard Temperature and Pressure
NIST: National Institute of Standards and Technology
TCD: Thermal Conductivity Detector
Gt: Gigatons
pA: Picoamphere
n/a: Not applicable
CAS: Chemical Abstracts Service
