# Supplementary material for: Innovative and cost-effective upgrading of crude biogenic pyrolysis oil using low-cost adsorbents and petroleum ether
Source: Environ Sci Pollut Res Int. 2025 Dec 17;32(56):30723–47. doi: 10.1007/s11356-025-37268-5 (PMC12804247; doi:10.1007/s11356-025-37268-5)
Supplement: Supplementary file 1 — Supplementary file1 (DOCX 14.0 KB ) [file 11356_2025_37268_MOESM1_ESM.docx]

**Supplementary Information**

**Innovative and cost-effective upgrading of crude biogenic pyrolysis oil using low-cost adsorbents and petroleum ether**

Akhil Mohan^*^(<https://orcid.org/0009-0002-7454-6797>), Department of Chemical Engineering, KTH Royal Institute of Technology, Stockholm, Sweden – 100 44

Alan Al-Wandi (Not Available), Department of Chemical Engineering, KTH Royal Institute of Technology, Stockholm, Sweden – 100 44

Åsa Emmer (<https://orcid.org/0000-0002-3444-9987>), Department of Chemistry, KTH Royal Institute of Technology, Stockholm, Sweden – 100 44

Klas Engvall (<https://orcid.org/0000-0002-6326-4084>), Department of Chemical Engineering, KTH Royal Institute of Technology, Stockholm, Sweden – 100 44

Mats Jonsson (<https://orcid.org/0000-0003-0663-0751>), Department of Chemistry, KTH Royal Institute of Technology, Stockholm, Sweden – 100 44

^*^Corresponding author: [akhilmo@kth.se](mailto:akhilmo@kth.se), +46 728340247

**LIST OF FIGURES**

1. **Surface characterization of calcium hydroxide**


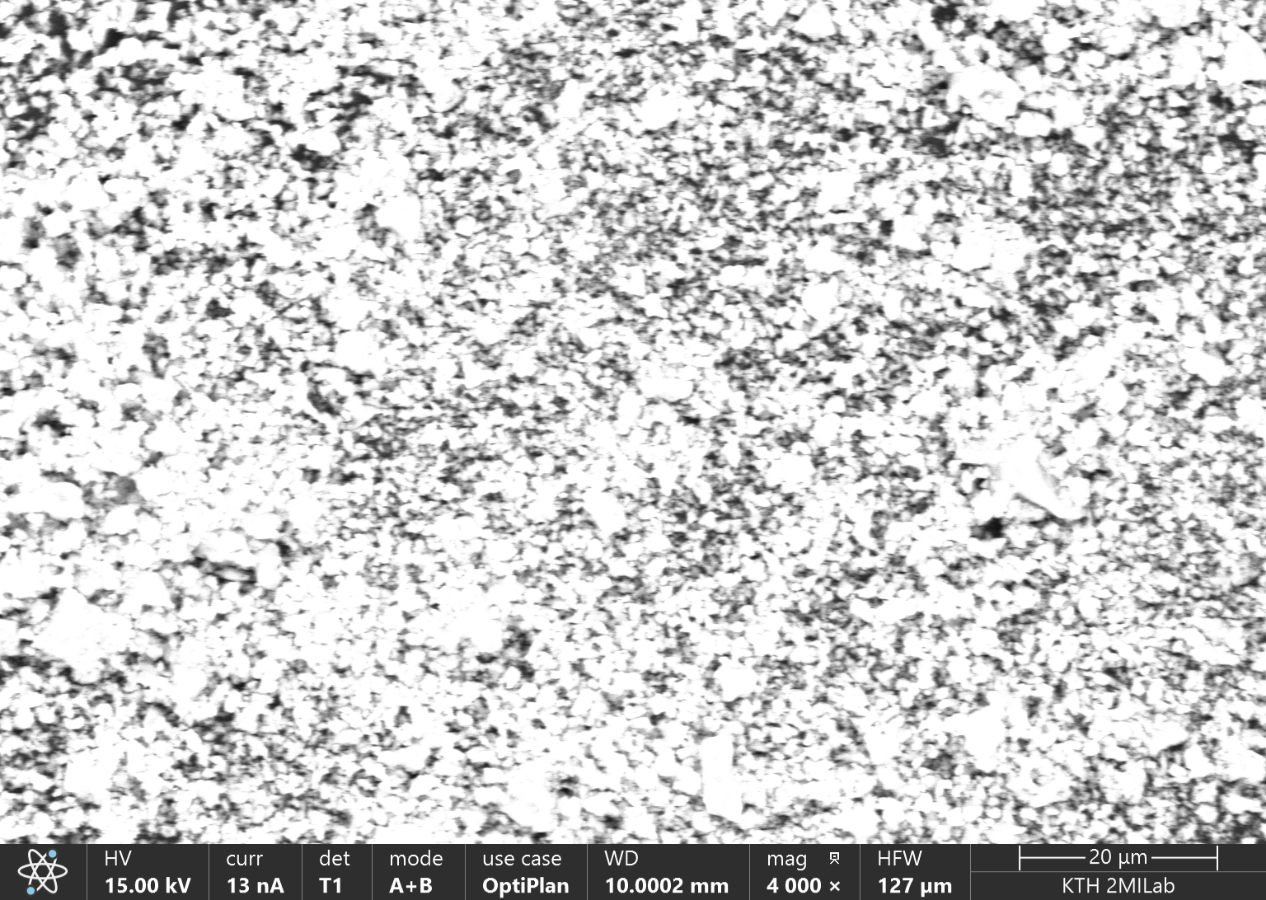


Total Number of Counts: 1 770 041
Average Count Rate: 130 593 cps
Acceleration Voltage: 15 Kv
Total Acquisition Time: 14 seconds

| **Element** | **Atomic %** | **Atomic % Error** | **Weight %** | **Weight % Error** | **Net Counts** |
| --- | --- | --- | --- | --- | --- |
| C | 17.8 | 0.1 | 10.6 | 0.0 | 192 743 |
| O | 61.7 | 0.3 | 48.9 | 0.3 | 208 627 |
| Mg | 0.2 | 0.0 | 0.3 | 0.0 | 5 430 |
| Al | 0.1 | 0.0 | 0.1 | 0.0 | 2 650 |
| Ca | 20.2 | 0.1 | 40.2 | 0.2 | 652 618 |


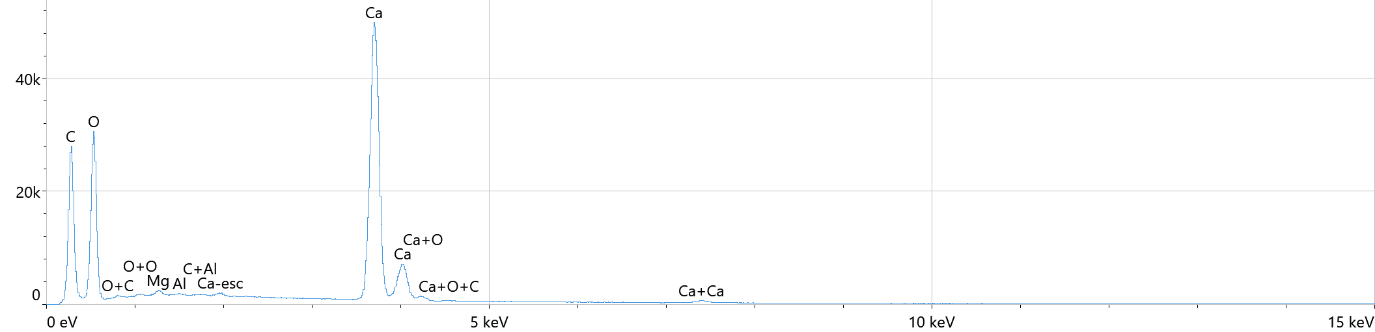


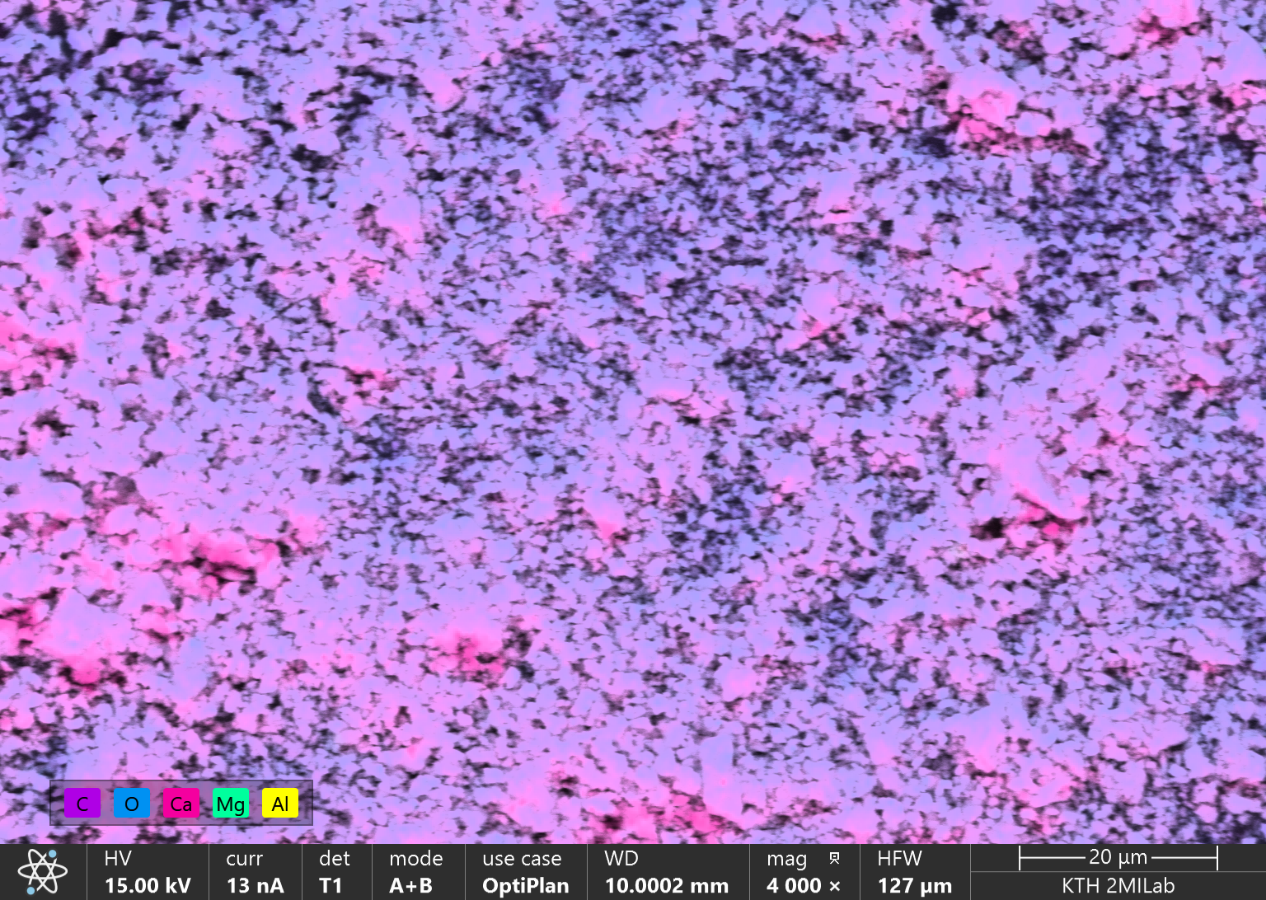


Maps Resolution: 768 x 512

| 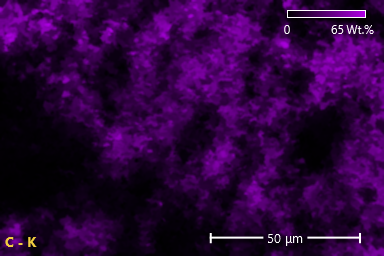 | 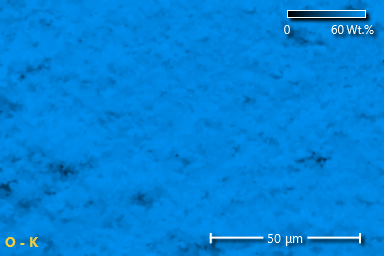 |
| --- | --- |
| 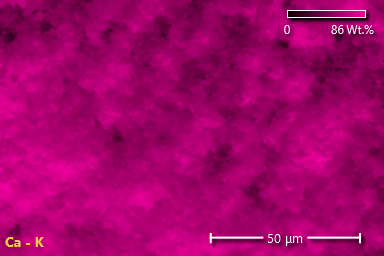 | 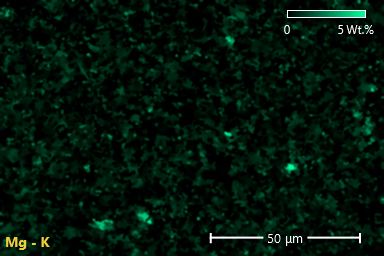 |
| 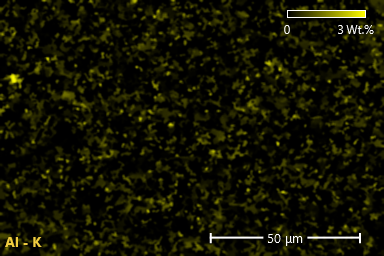 |  |

1. **Surface characterization of red mud**


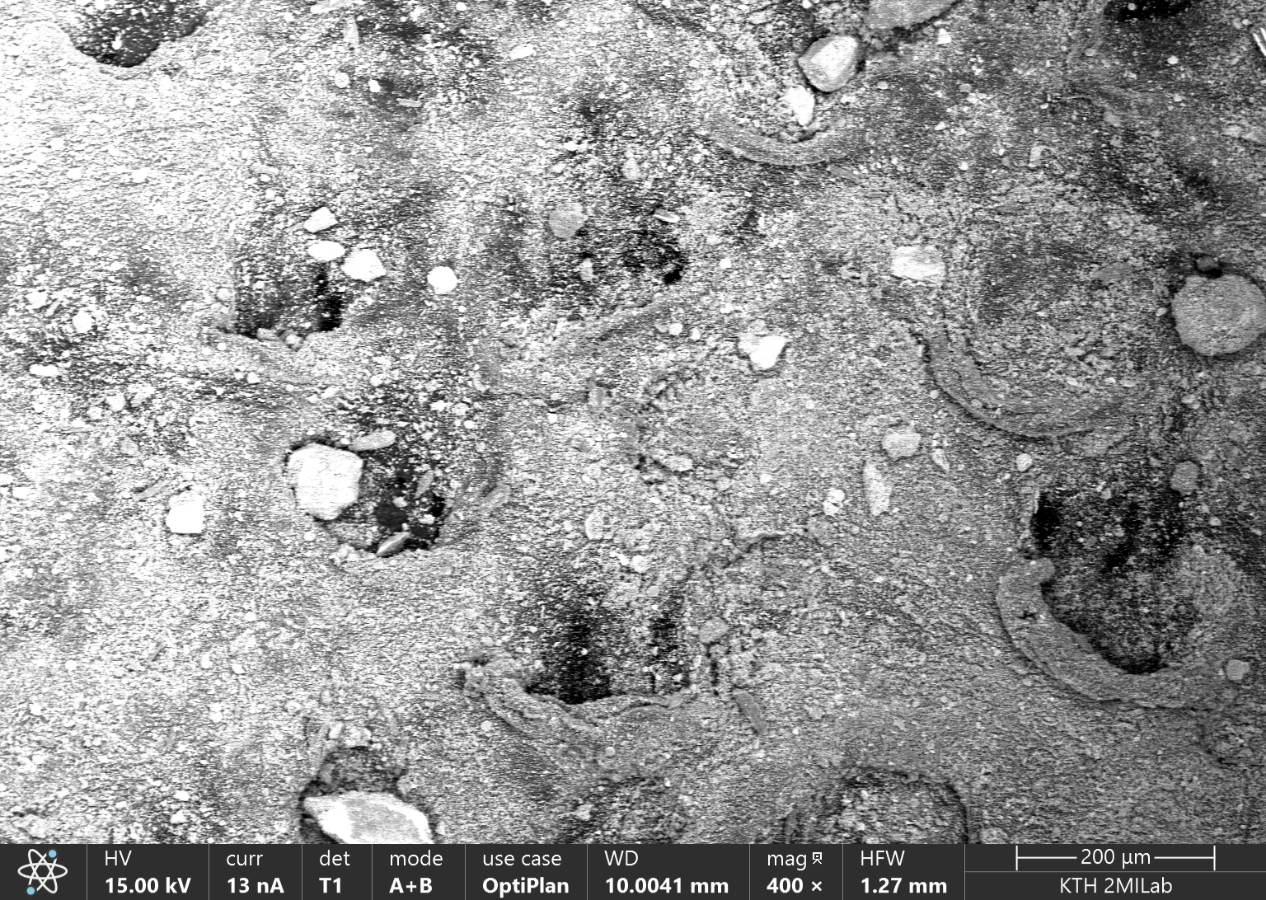


Total Number of Counts: 2 296 536
Average Count Rate: 66 724 cps
Acceleration Voltage: 15 kV
Total Acquisition Time: 34 seconds

| **Element** | **Atomic %** | **Atomic % Error** | **Weight %** | **Weight % Error** | **Net Counts** |
| --- | --- | --- | --- | --- | --- |
| O | 54.1 | 0.3 | 42.1 | 0.2 | 535 193 |
| Mg | 0.1 | 0.0 | 0.2 | 0.0 | 4 194 |
| Ca | 0.9 | 0.0 | 1.8 | 0.0 | 38 625 |
| C | 19.2 | 0.1 | 11.2 | 0.1 | 117 166 |
| Ti | 1.0 | 0.0 | 2.4 | 0.0 | 34 337 |
| Fe | 7.9 | 0.0 | 21.4 | 0.1 | 147 104 |
| Na | 6.8 | 0.0 | 7.6 | 0.0 | 140 283 |
| Al | 5.8 | 0.0 | 7.7 | 0.0 | 229 304 |
| Si | 4.2 | 0.0 | 5.7 | 0.0 | 172 798 |


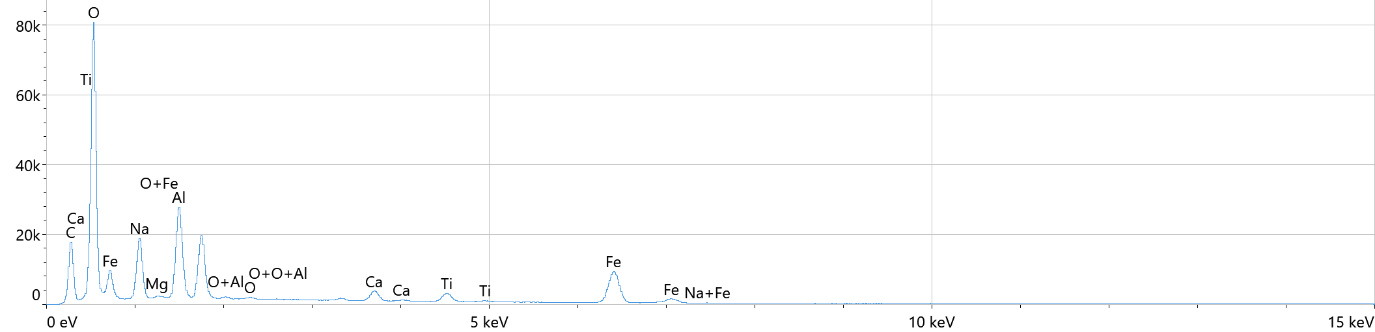


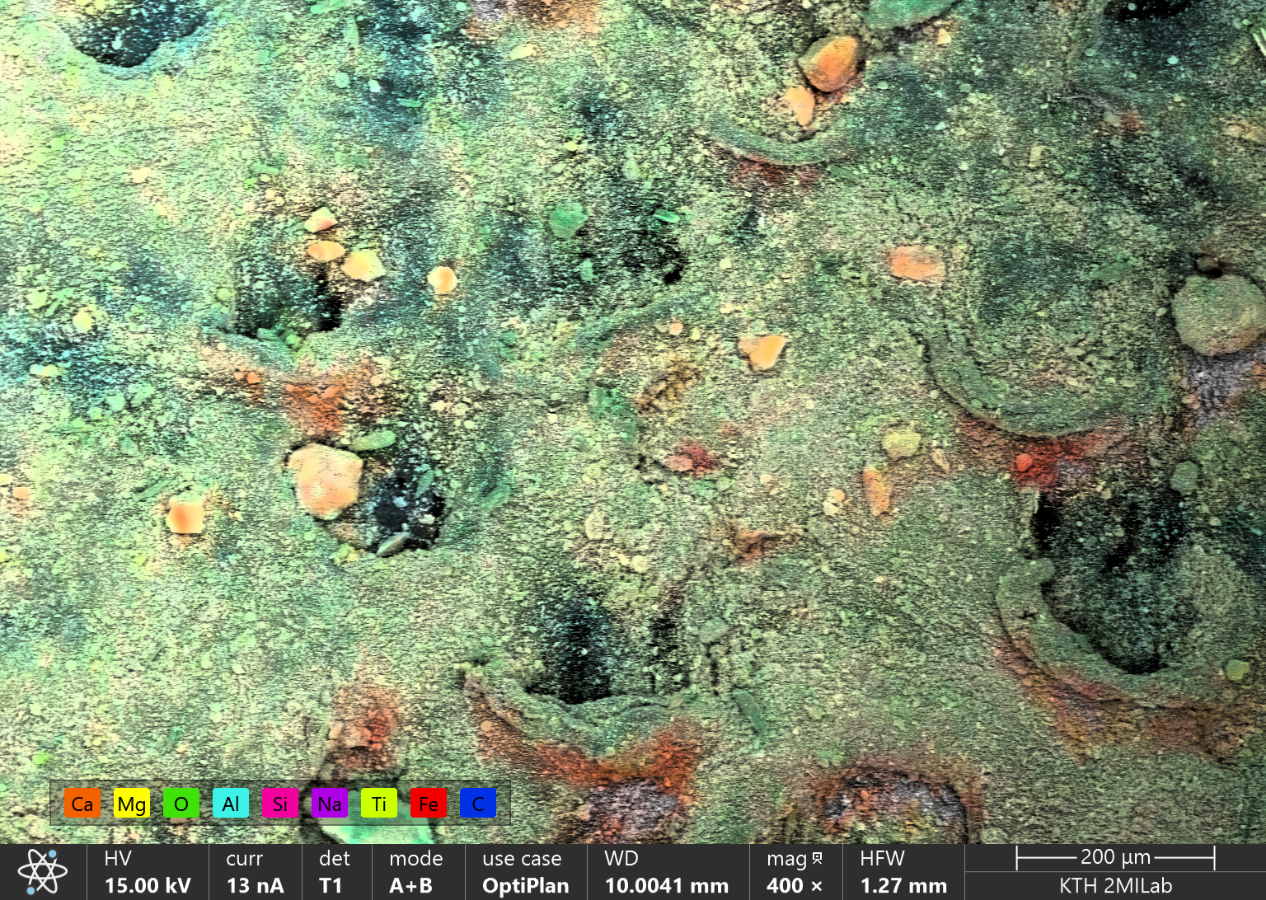


Maps Resolution: 768 x 512

| 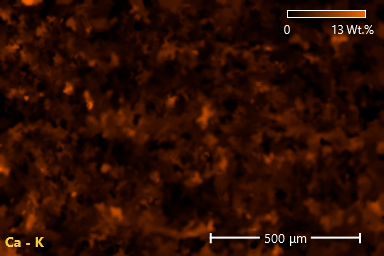 | 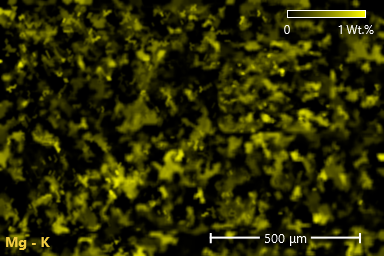 |
| --- | --- |
| 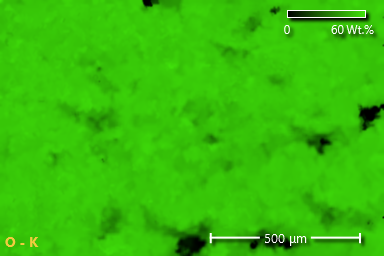 | 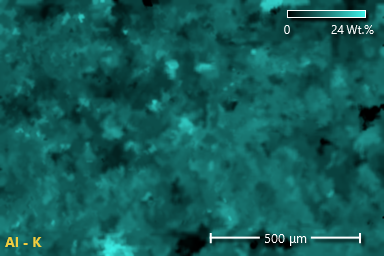 |
| 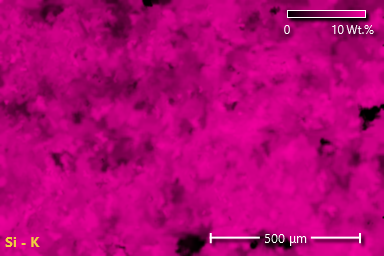 | 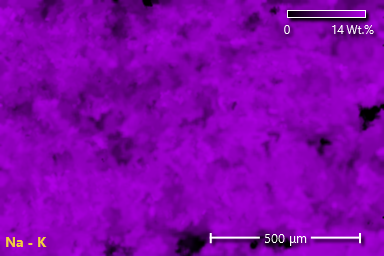 |
| 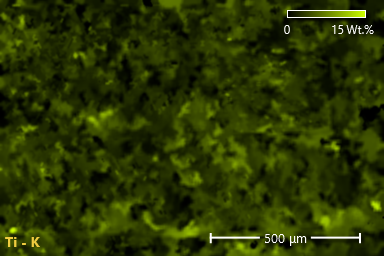 | 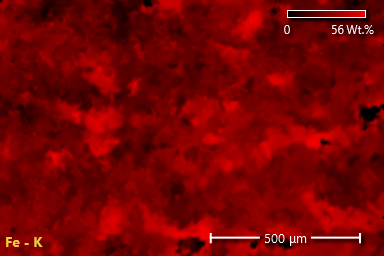 |
| 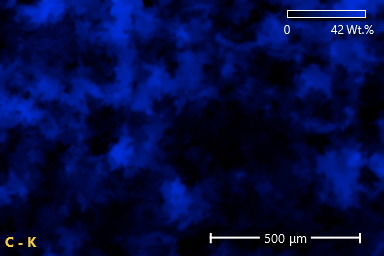 |  |

**3. Surface characterization of bentonite**


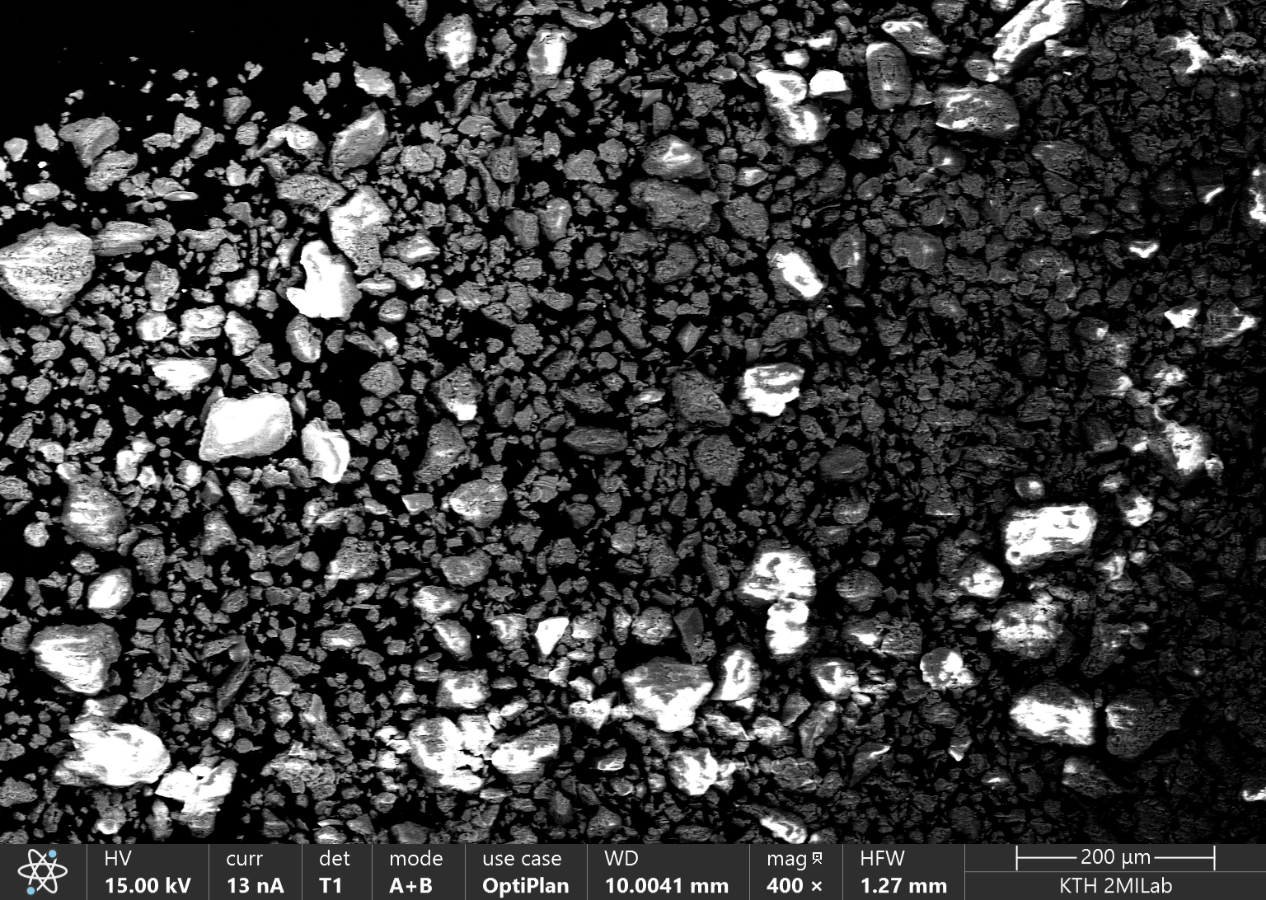


Total Number of Counts: 6 905 317
Average Count Rate: 64 702 cps
Acceleration Voltage: 15 kV
Total Acquisition Time: 103 seconds

| **Element** | **Atomic %** | **Atomic % Error** | **Weight %** | **Weight % Error** | **Net Counts** |
| --- | --- | --- | --- | --- | --- |
| O | 69.0 | 0.3 | 54.6 | 0.3 | 1 431 773 |
| Mg | 0.7 | 0.0 | 0.8 | 0.0 | 46 166 |
| Ca | 0.6 | 0.0 | 1.2 | 0.0 | 46 447 |
| Ti | 0.1 | 0.0 | 0.3 | 0.0 | 8 364 |
| Fe | 1.7 | 0.0 | 4.8 | 0.1 | 60 193 |
| Al | 7.7 | 0.0 | 10.3 | 0.0 | 693 813 |
| Si | 20.1 | 0.1 | 28.0 | 0.1 | 1 746 450 |


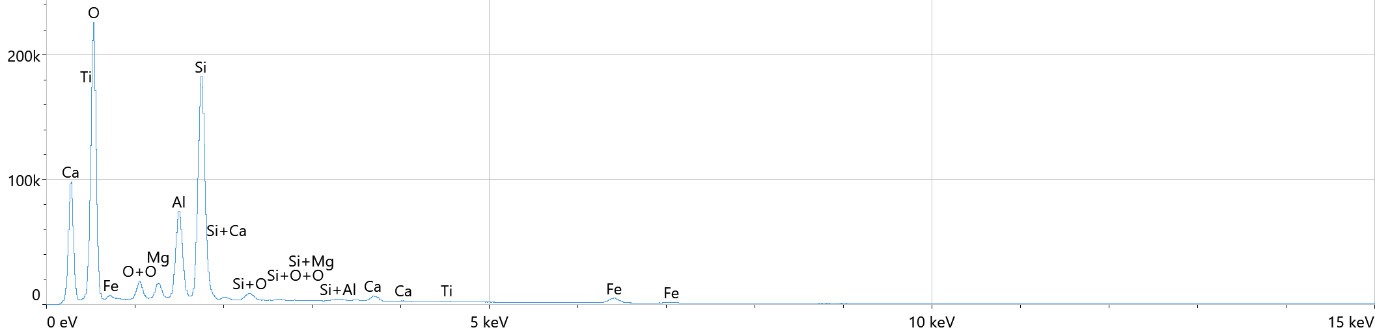


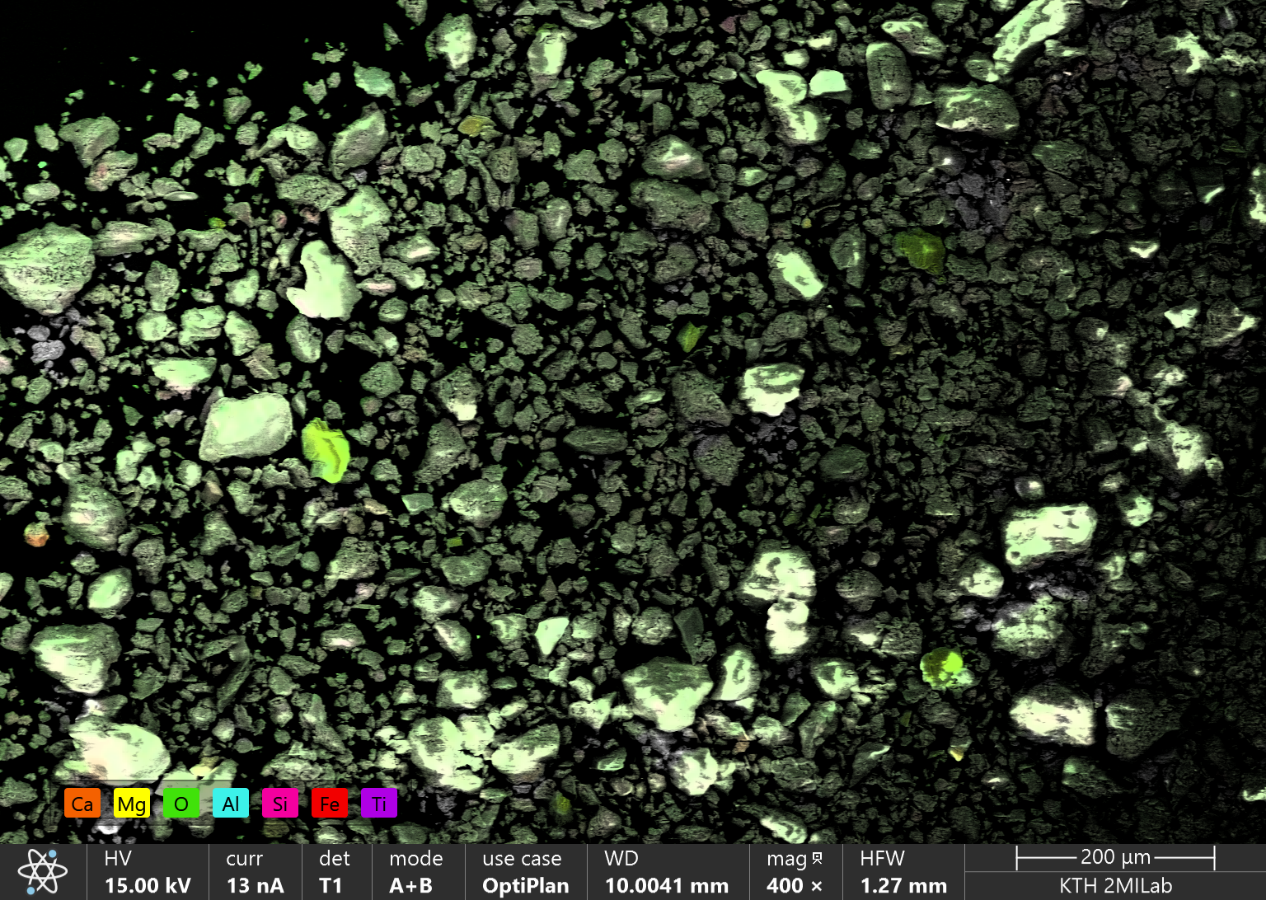


Maps Resolution: 768 x 512

| 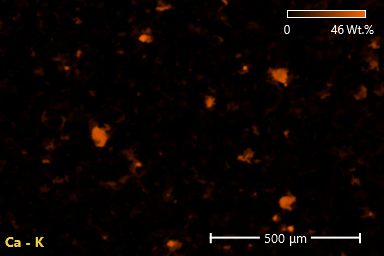 | 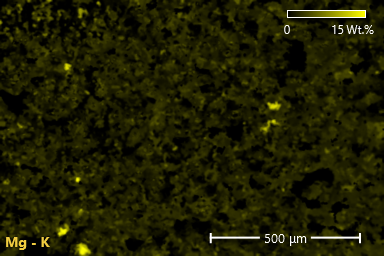 |
| --- | --- |
| 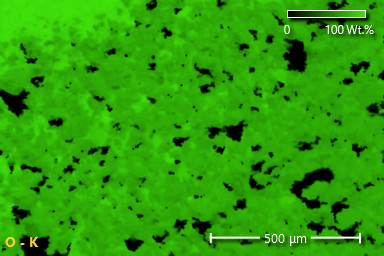 | 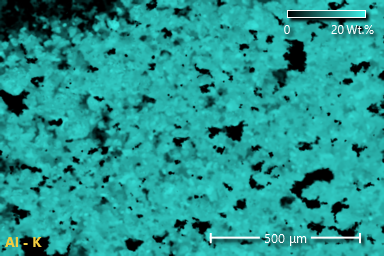 |
| 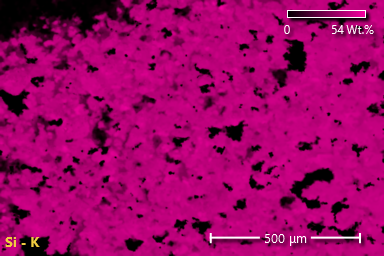 | 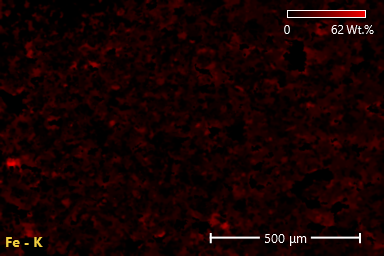 |
| 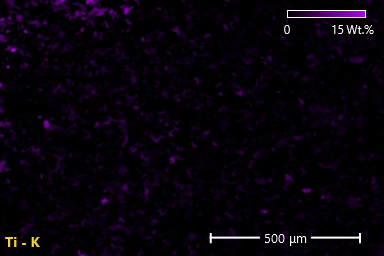 |  |

**4. Surface characterization of dolomite**


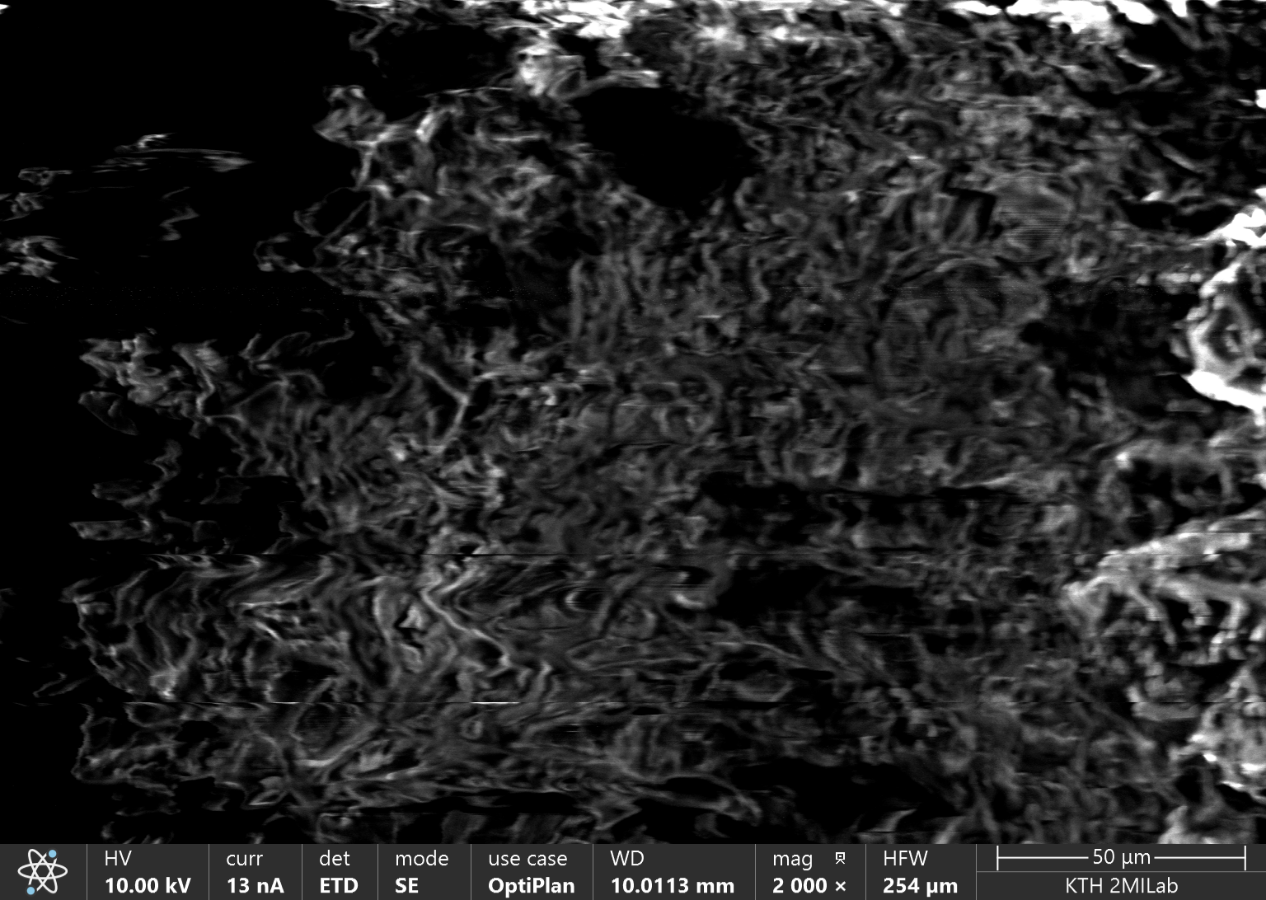


Total Number of Counts: 792 688
Average Count Rate: 48 651 cps
Acceleration Voltage: 10 kV
Total Acquisition Time: 15 seconds

| **Element** | **Atomic %** | **Atomic % Error** | **Weight %** | **Weight % Error** | **Net Counts** |
| --- | --- | --- | --- | --- | --- |
| O | 97.4 | 0.6 | 94.9 | 0.6 | 93 218 |
| Mg | 0.5 | 0.1 | 0.8 | 0.1 | 776 |
| Ca | 0.2 | 0.1 | 0.4 | 0.2 | 161 |
| K | 0.9 | 0.1 | 2.2 | 0.2 | 1 021 |
| Al | 0.5 | 0.1 | 0.9 | 0.2 | 882 |
| S | 0.4 | 0.1 | 0.8 | 0.1 | 701 |


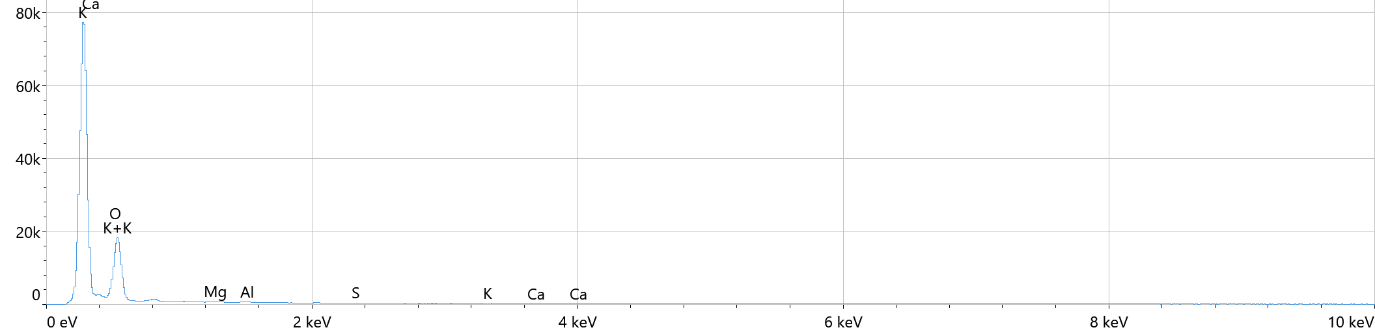


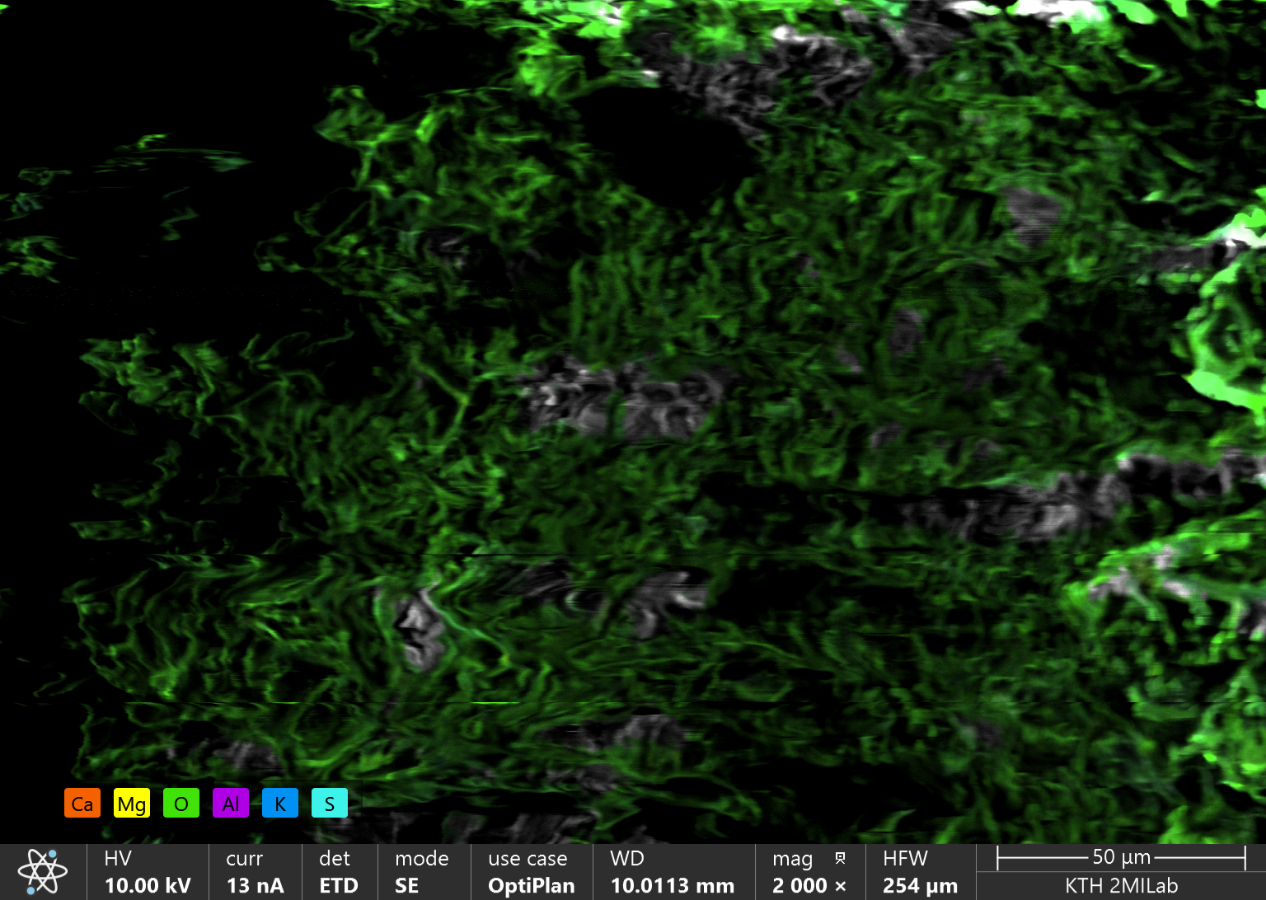


Maps Resolution: 768 x 512

| 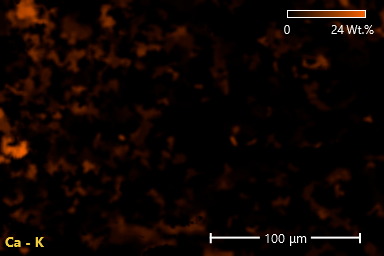 | 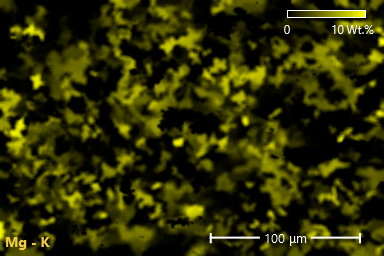 |
| --- | --- |
| 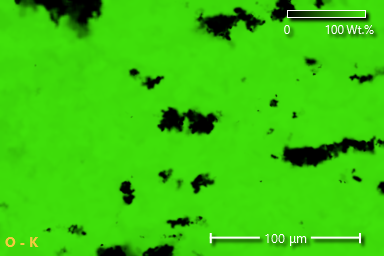 | 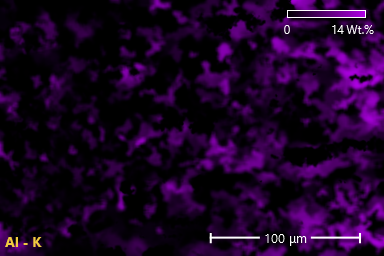 |
| 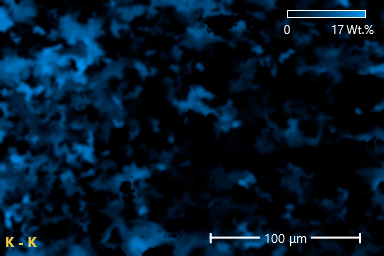 | 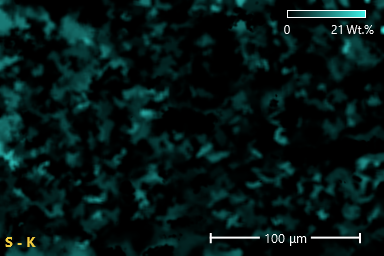 |

**5. Surface characterization of used silica**


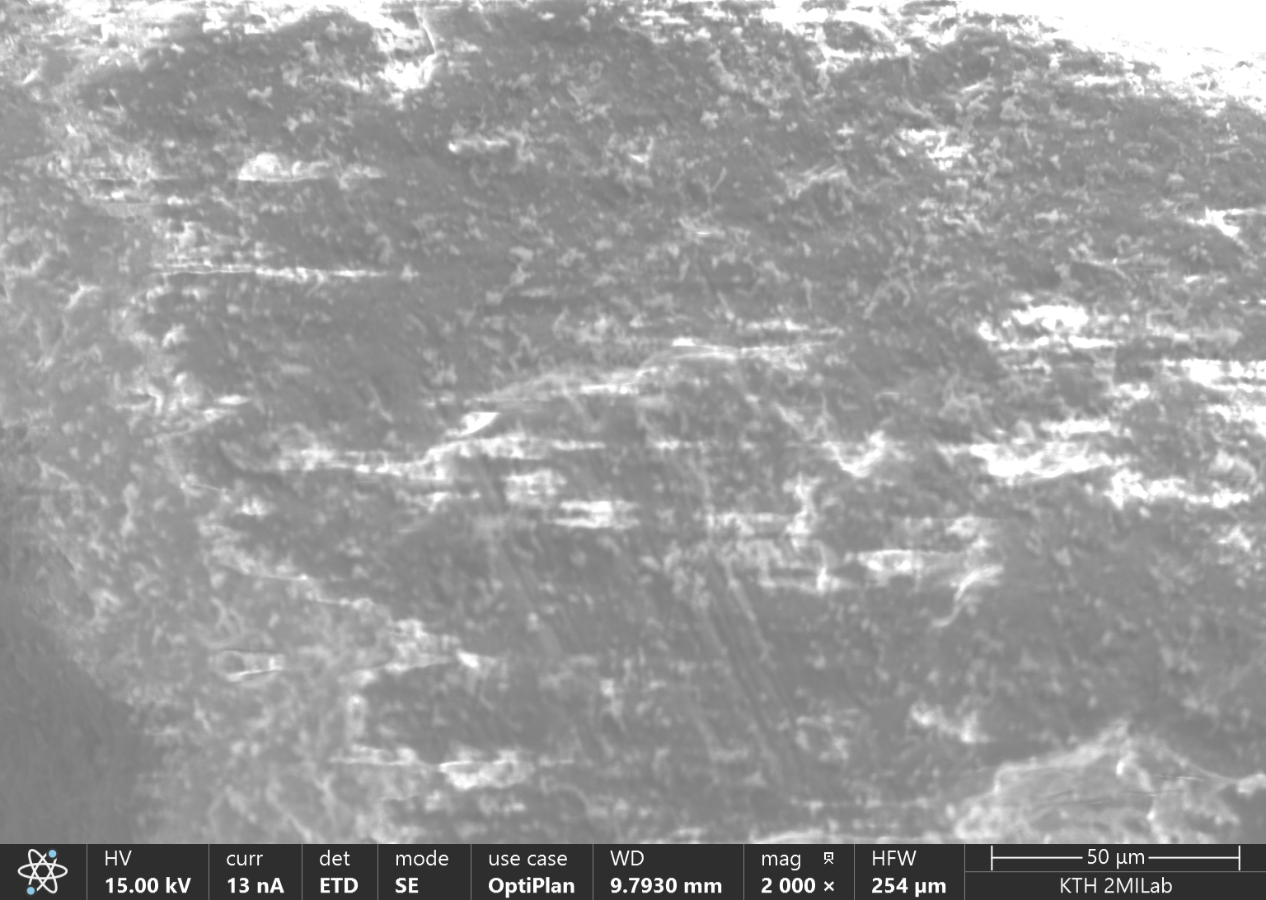


Total Number of Counts: 14 307 056
Average Count Rate: 184 969 cps
Acceleration Voltage: 15 kV
Total Acquisition Time: 78 seconds

| **Element** | **Atomic %** | **Atomic % Error** | **Weight %** | **Weight % Error** | **Net Counts** |
| --- | --- | --- | --- | --- | --- |
| O | 69.1 | 0.3 | 52.9 | 0.2 | 2 787 410 |
| Mg | 0.5 | 0.0 | 0.6 | 0.0 | 90 418 |
| Ca | 1.3 | 0.0 | 2.4 | 0.0 | 237 325 |
| Al | 6.1 | 0.0 | 7.9 | 0.0 | 1 379 247 |
| Si | 17.7 | 0.1 | 23.8 | 0.1 | 3 953 483 |
| K | 5.0 | 0.0 | 9.3 | 0.0 | 1 039 090 |
| Pu | 0.3 | 0.0 | 3.1 | 0.2 | 113 875 |


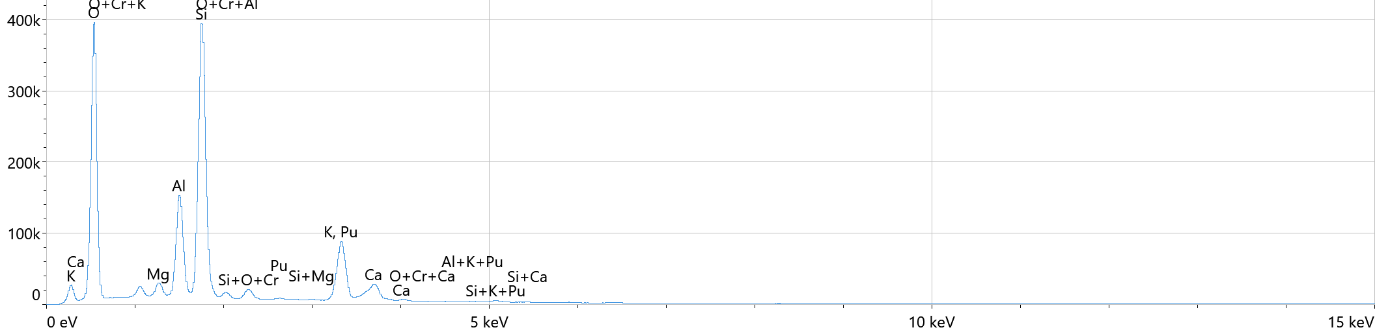


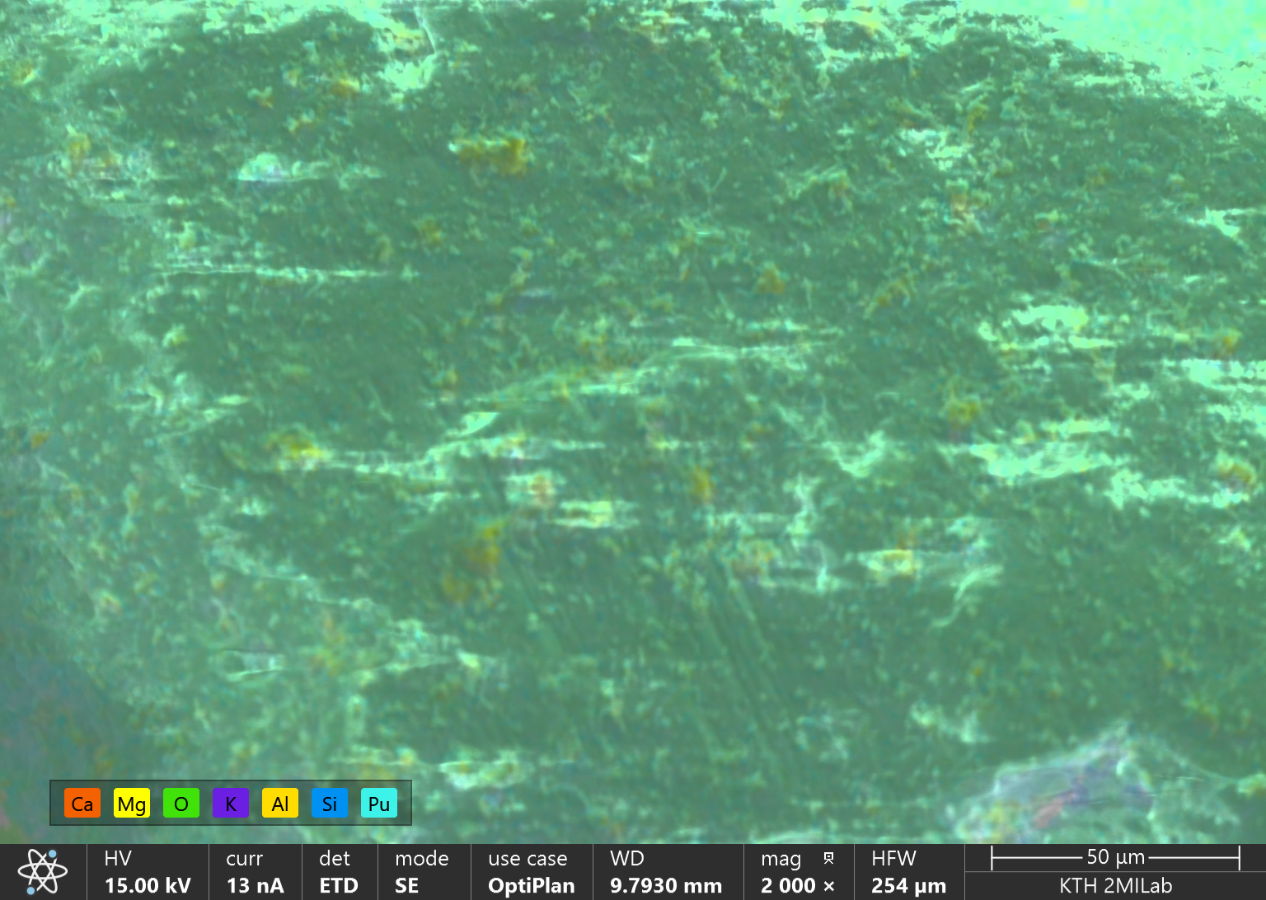


Maps Resolution: 768 x 512

| 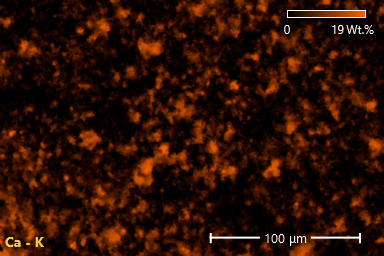 | 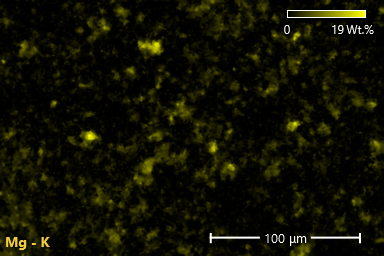 |
| --- | --- |
| 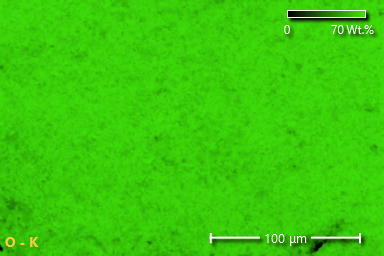 | 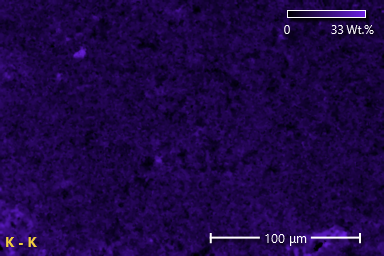 |
| 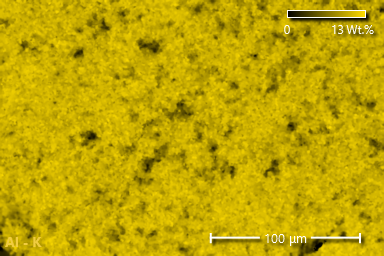 | 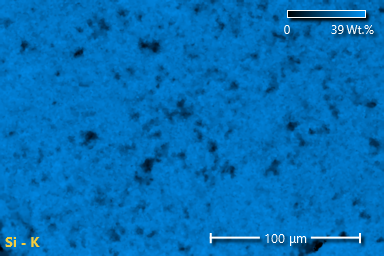 |
| 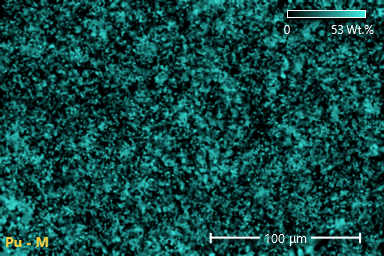 |  |

**6. Surface characterization of new silica**


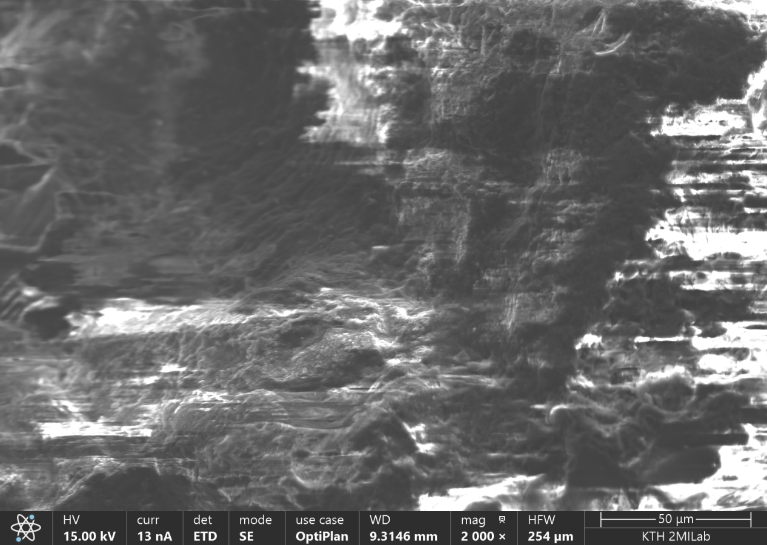


Total Number of Counts: 31 732 164
Average Count Rate: 192 547 cps
Acceleration Voltage: 15 kV
Total Acquisition Time: 160 seconds

| **Element** | **Atomic %** | **Atomic % Error** | **Weight %** | **Weight % Error** | **Net Counts** |
| --- | --- | --- | --- | --- | --- |
| O | 70.2 | 0.3 | 56.4 | 0.3 | 7 767 421 |
| Mg | 0.5 | 0.0 | 0.6 | 0.0 | 180 824 |
| Ca | 0.0 | --- | 0.0 | --- | 0 |
| K | 1.2 | 0.0 | 2.3 | 0.0 | 511 757 |
| Al | 5.0 | 0.0 | 6.7 | 0.0 | 2 428 265 |
| Si | 22.1 | 0.1 | 31.2 | 0.1 | 10 725 387 |
| Fe | 1.0 | 0.0 | 2.8 | 0.0 | 181 012 |


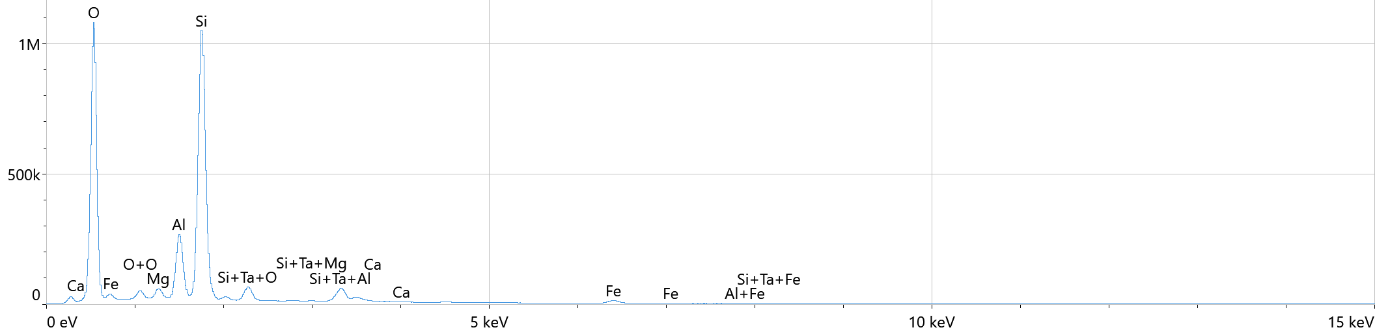


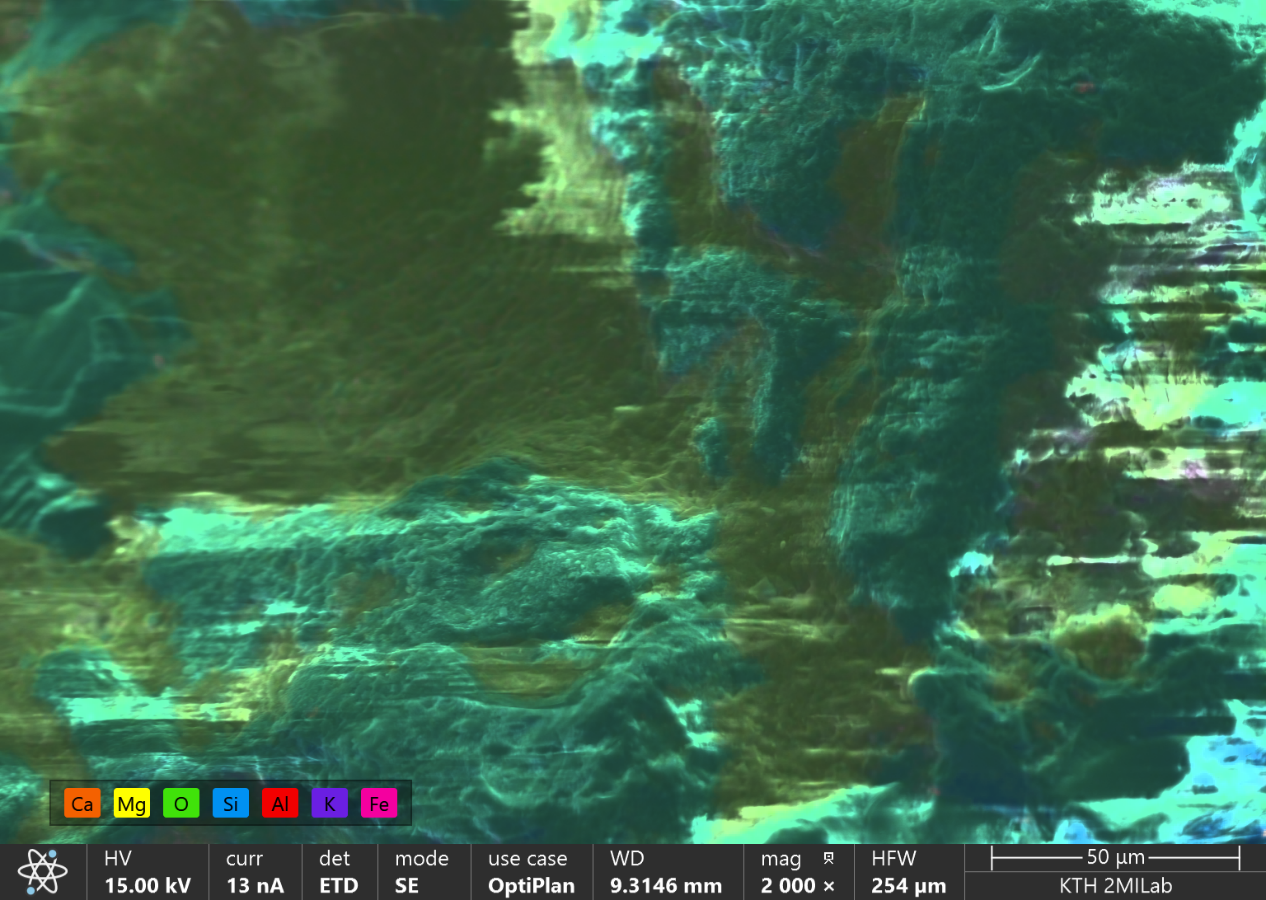


Maps Resolution: 768 x 512

| 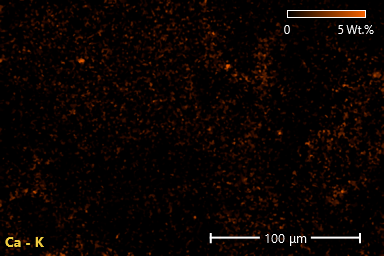 | 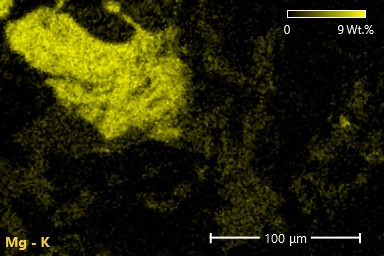 |
| --- | --- |
| 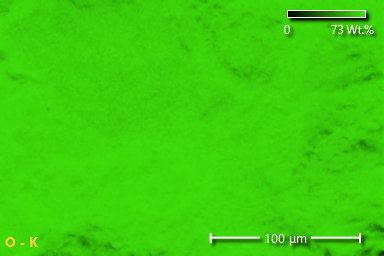 | 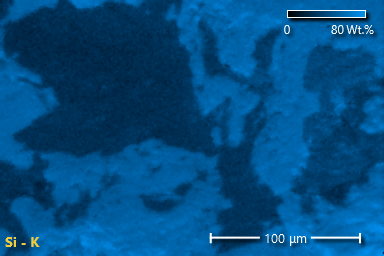 |
| 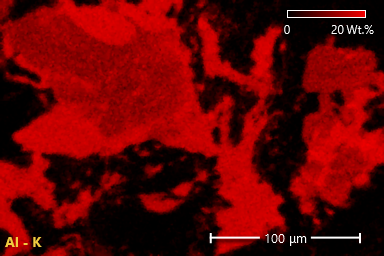 | 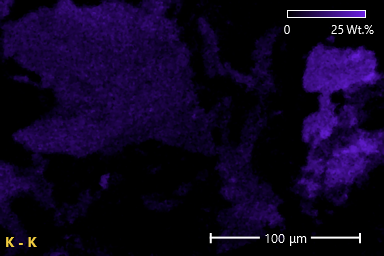 |
| 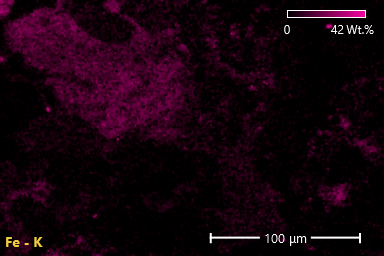 |  |

**Fig. S1** Quant mapping and elemental analysis of various adsorbents

**7. Nitrogen adsorption-desorption isothermal plots (BET)**

**NS**

**Fig. S2** Nitrogen adsorption-desorption isothermal plot of various adsorbents

**8. Gas Chromatography Mass Spectrometry**


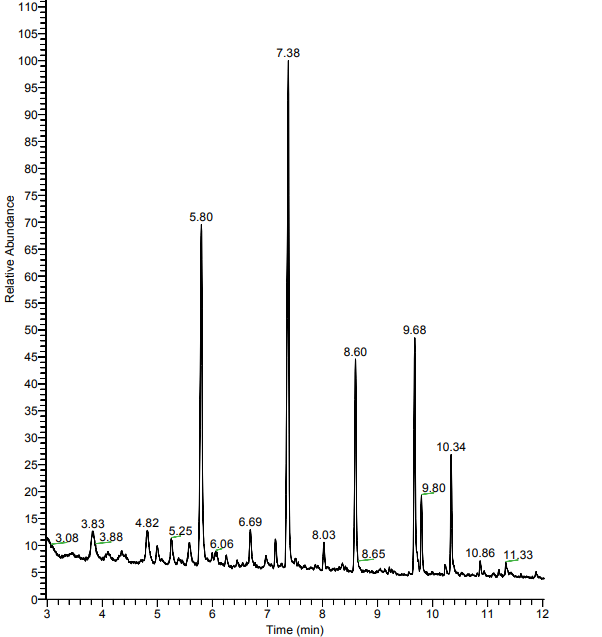

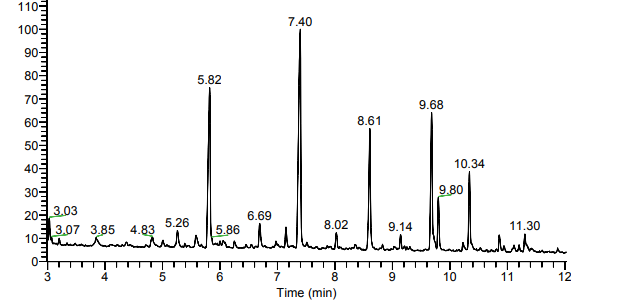


**CH**

**CO**

**BT**

**RM**


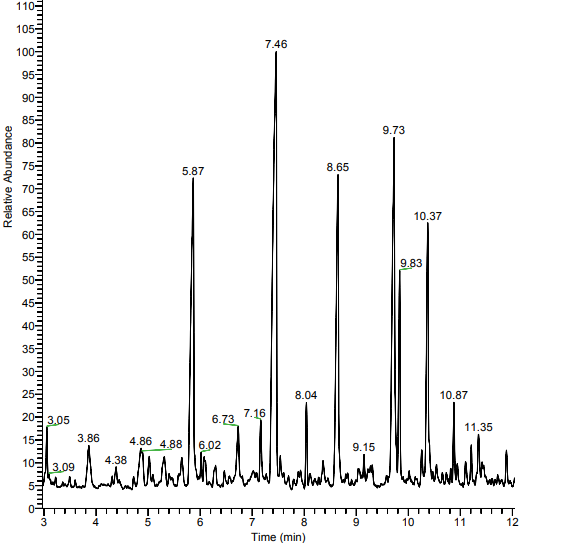

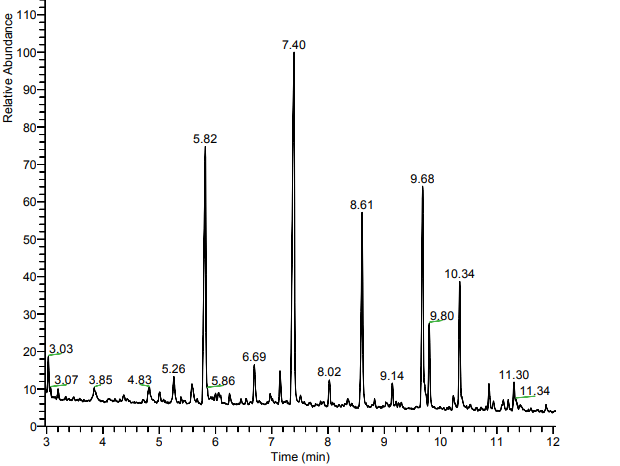


**DM**

**US**


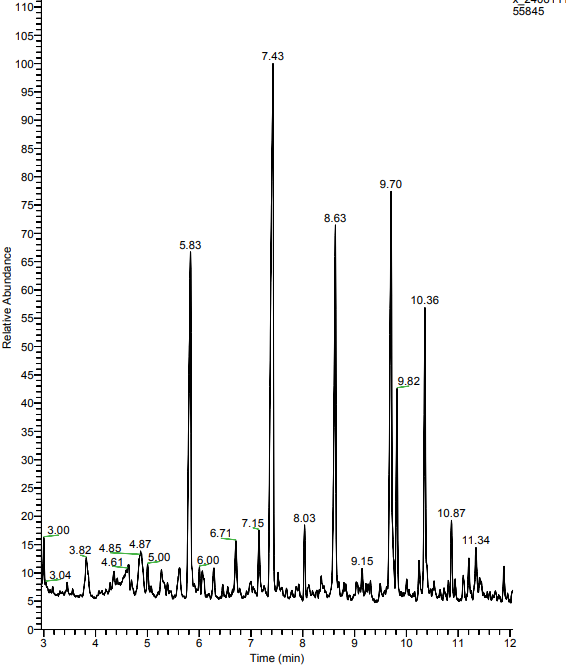

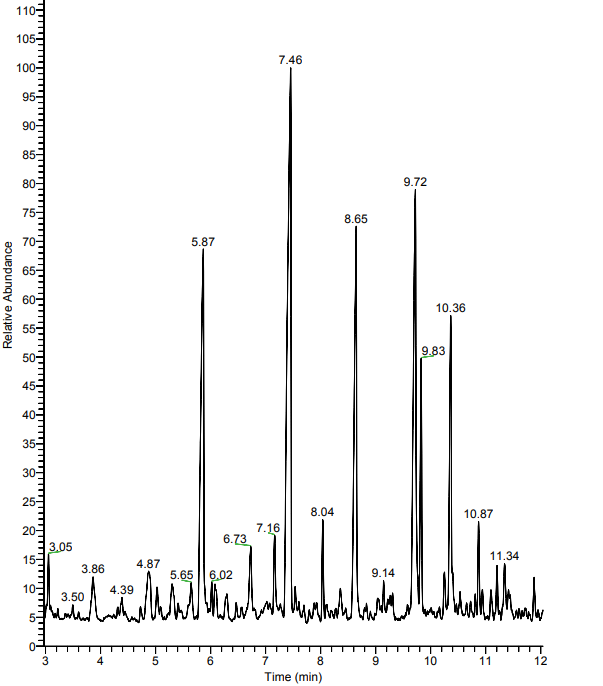


**PE**

**NS**


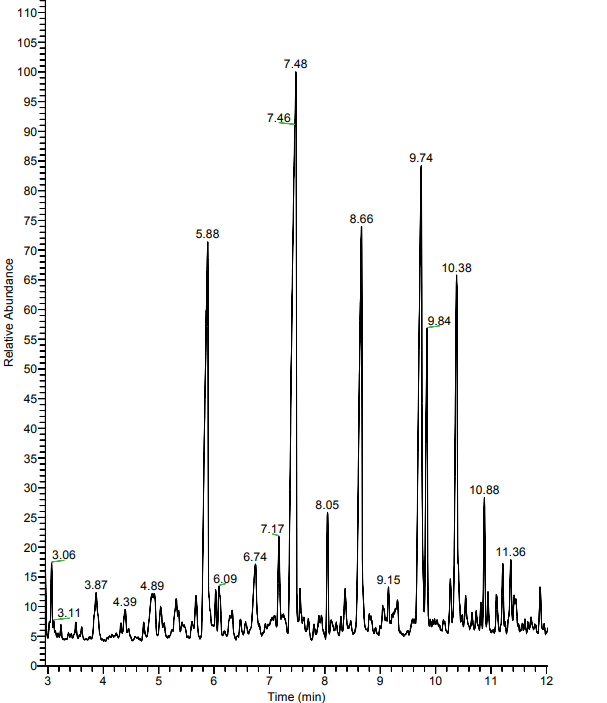

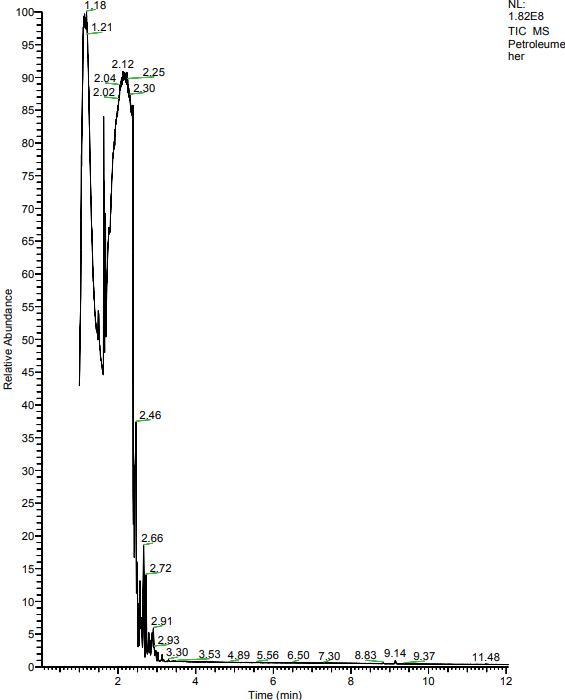


(a)

**
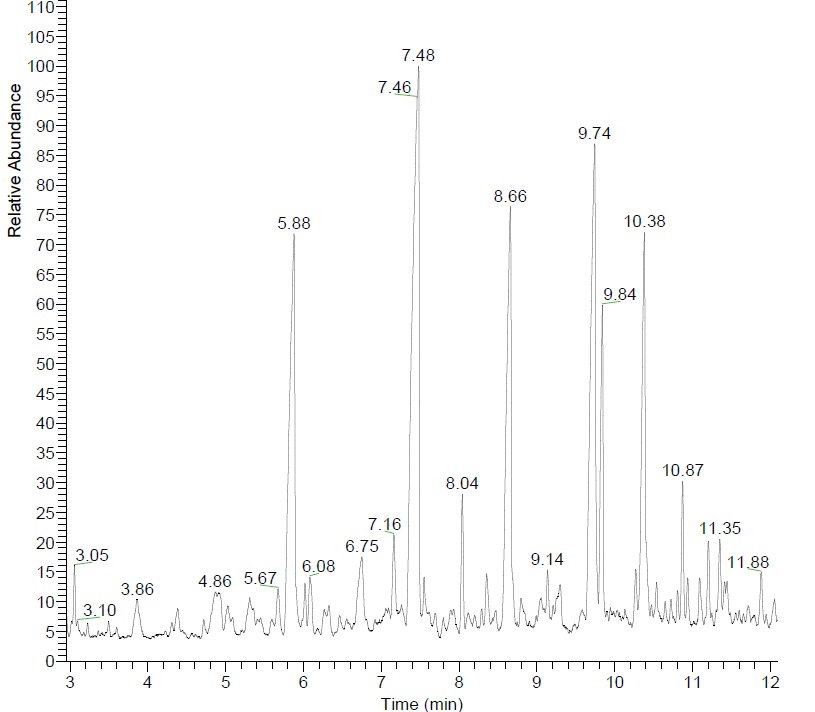
**

**SP-CO**

**Fig. S3** GC-MS Spectra of crude and upgraded oils (PE – Petroleum ether)

**9. Biomass Technology Group**

Pyrolysis of pine sawdust at a temperature of 400-600 ^o^C in an oxygen-free atmosphere yields 67.3, 14.7, and 18 wt.% for oil, gas, and char, respectively [Yildiz et al. 2011; Van de Beld and Muggen 2015]. Nevertheless, the authors have not reported the extensive composition of the byproducts. The specified pyrolysis conditions are used in pyrolysis is due to higher liquid yield (65 wt.%). BTG uses a rotating cone reactor without any inert gas for pyrolysis reaction (characteristic feature compared to other pyrolysis technologies), which improves heat and mass transfer through the effective mixing of biomass with sand.

**9. Nature of calcium hydroxide after the upgrading process**


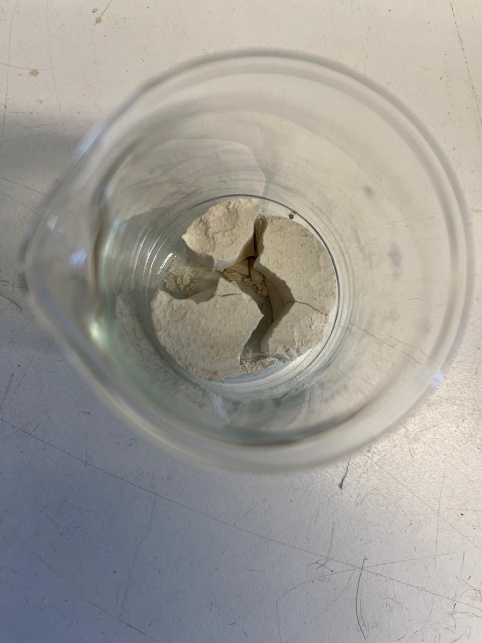


**Fig. S4** Solid structure of calcium hydroxide

**Spot No. 5**


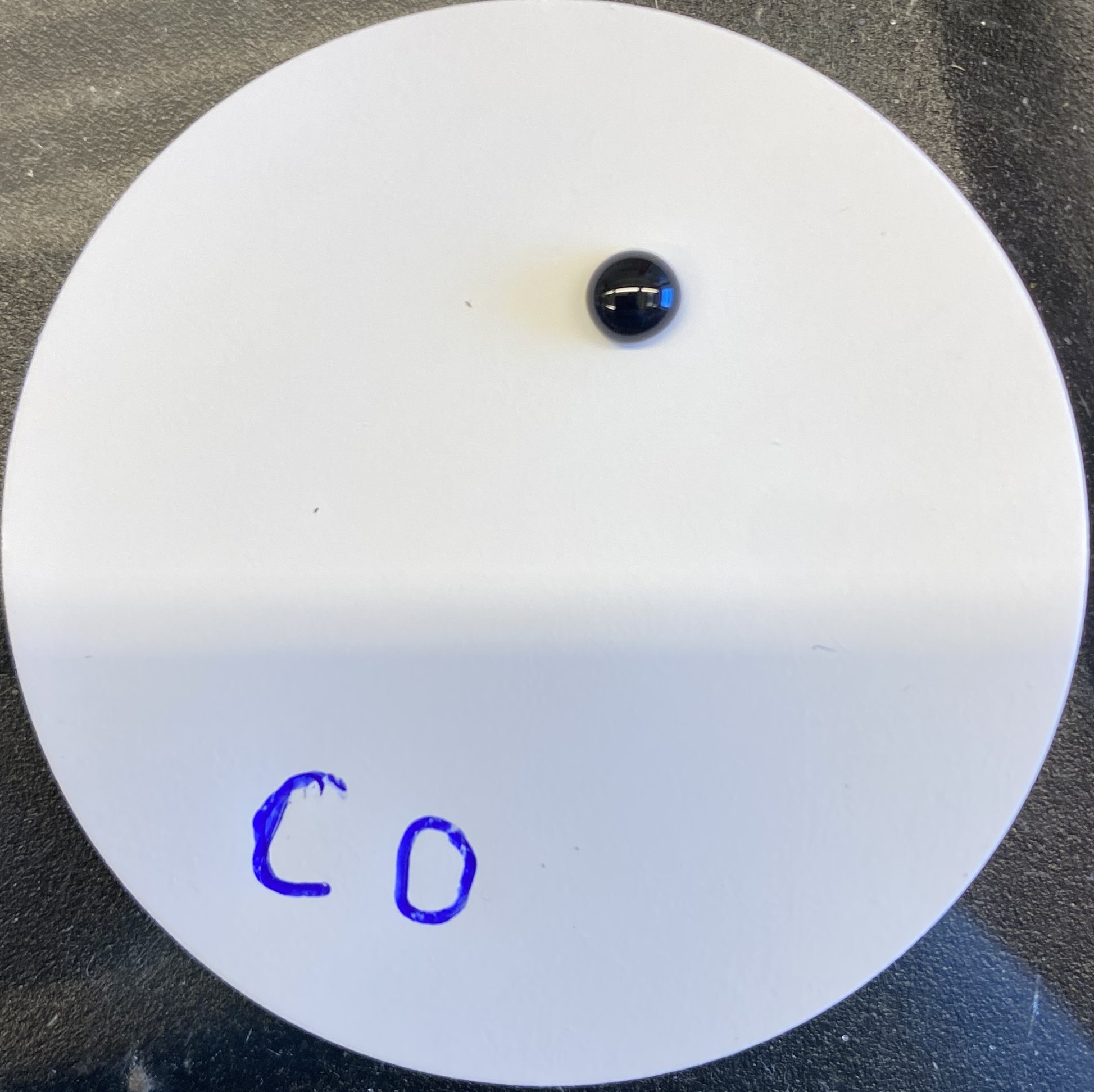

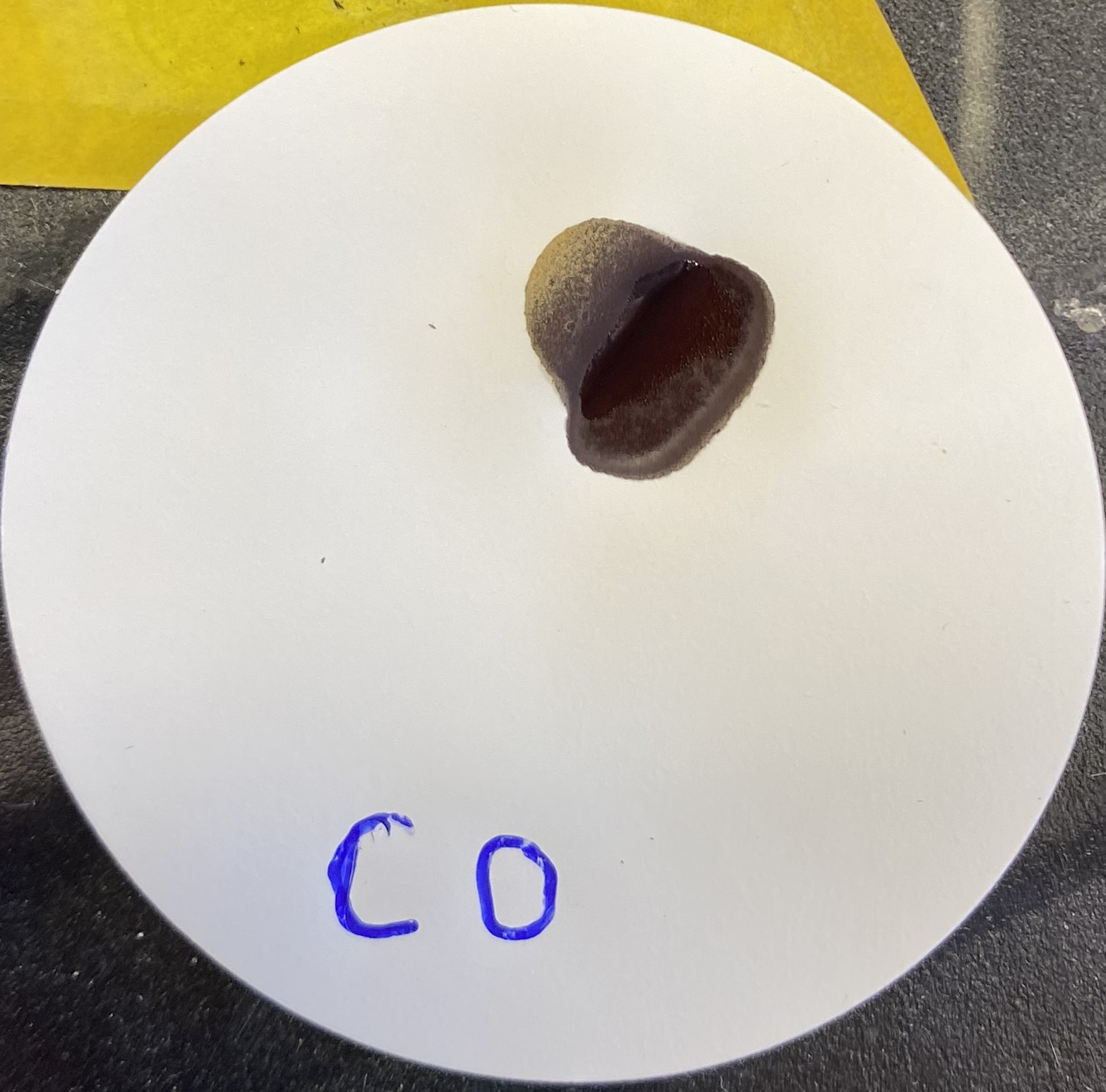

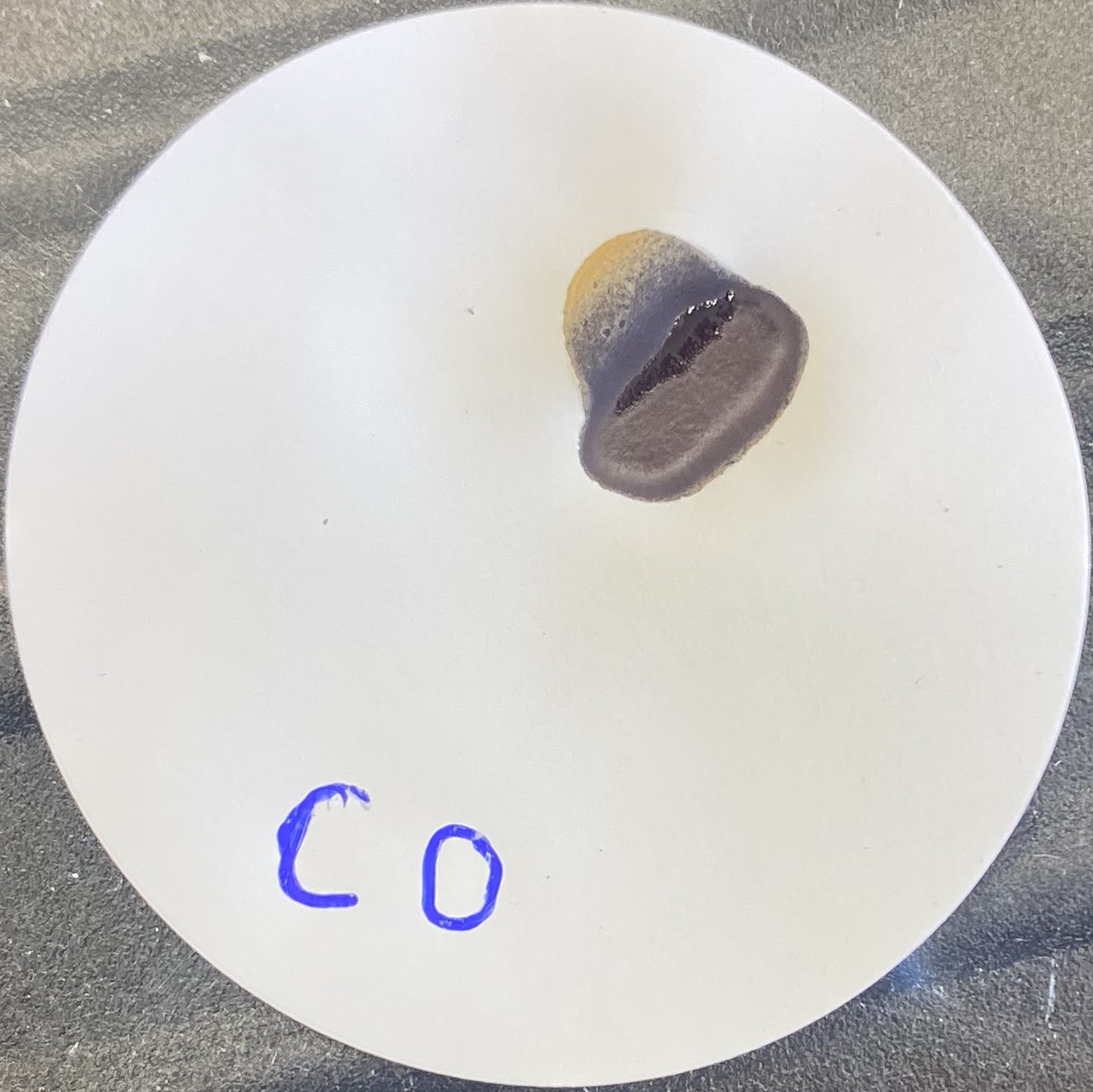

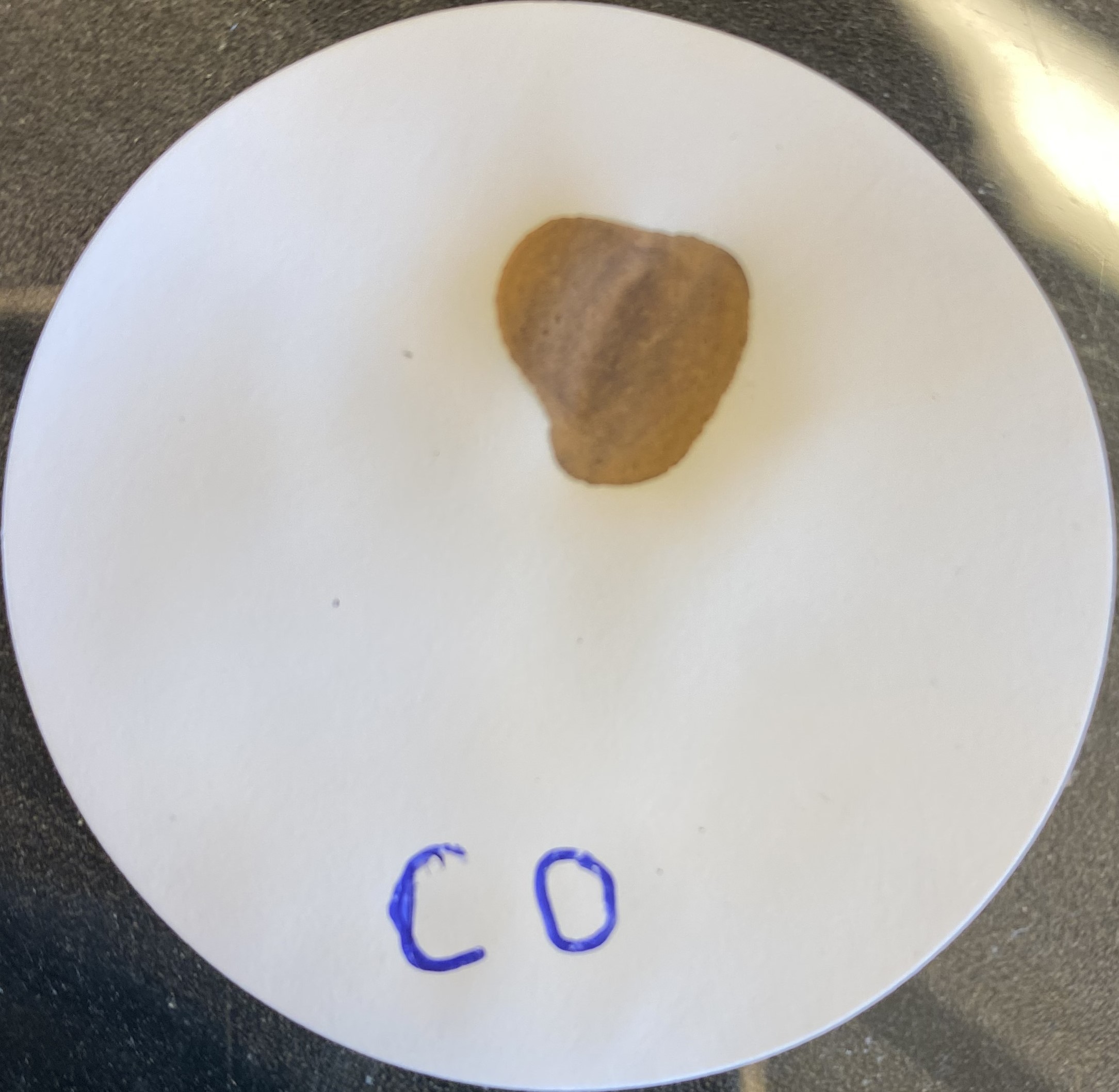

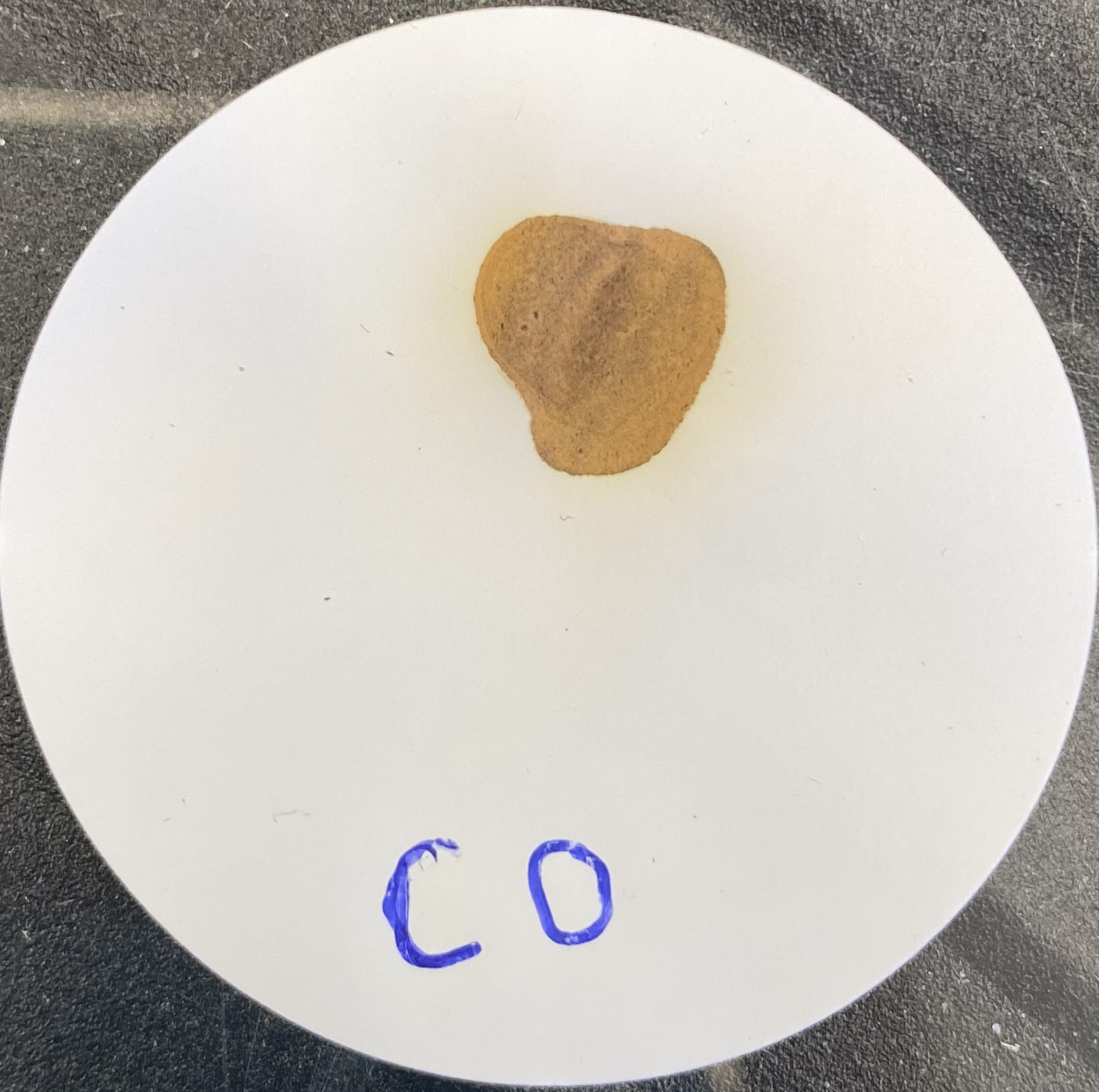


**Fig. S5** ASTM D4740 spot test of CO (a) 1 min, (b) 30 min, (c) 60 min, (d) 20 h, (e) 24h (left to right)

**Spot No. 1**


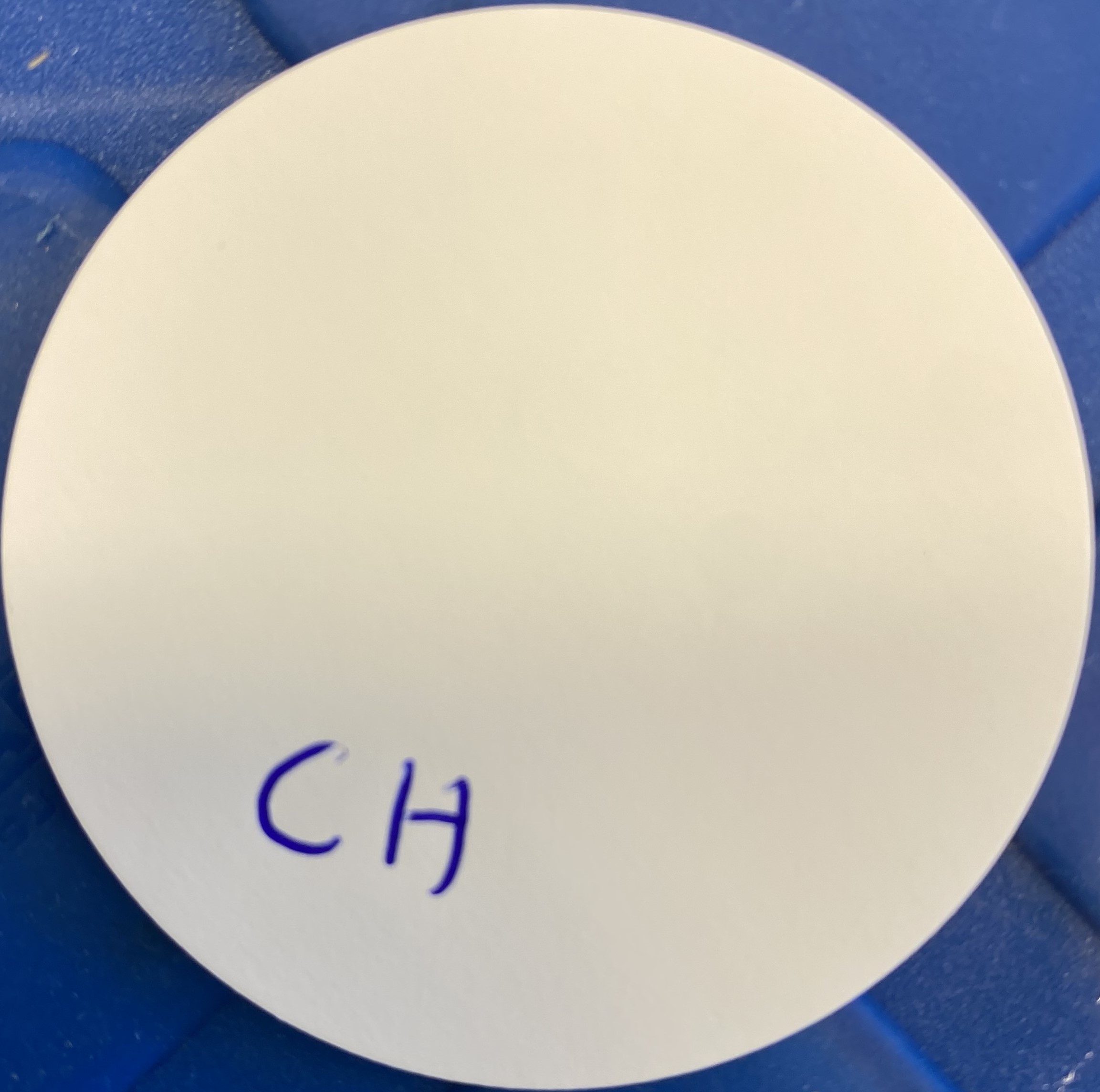

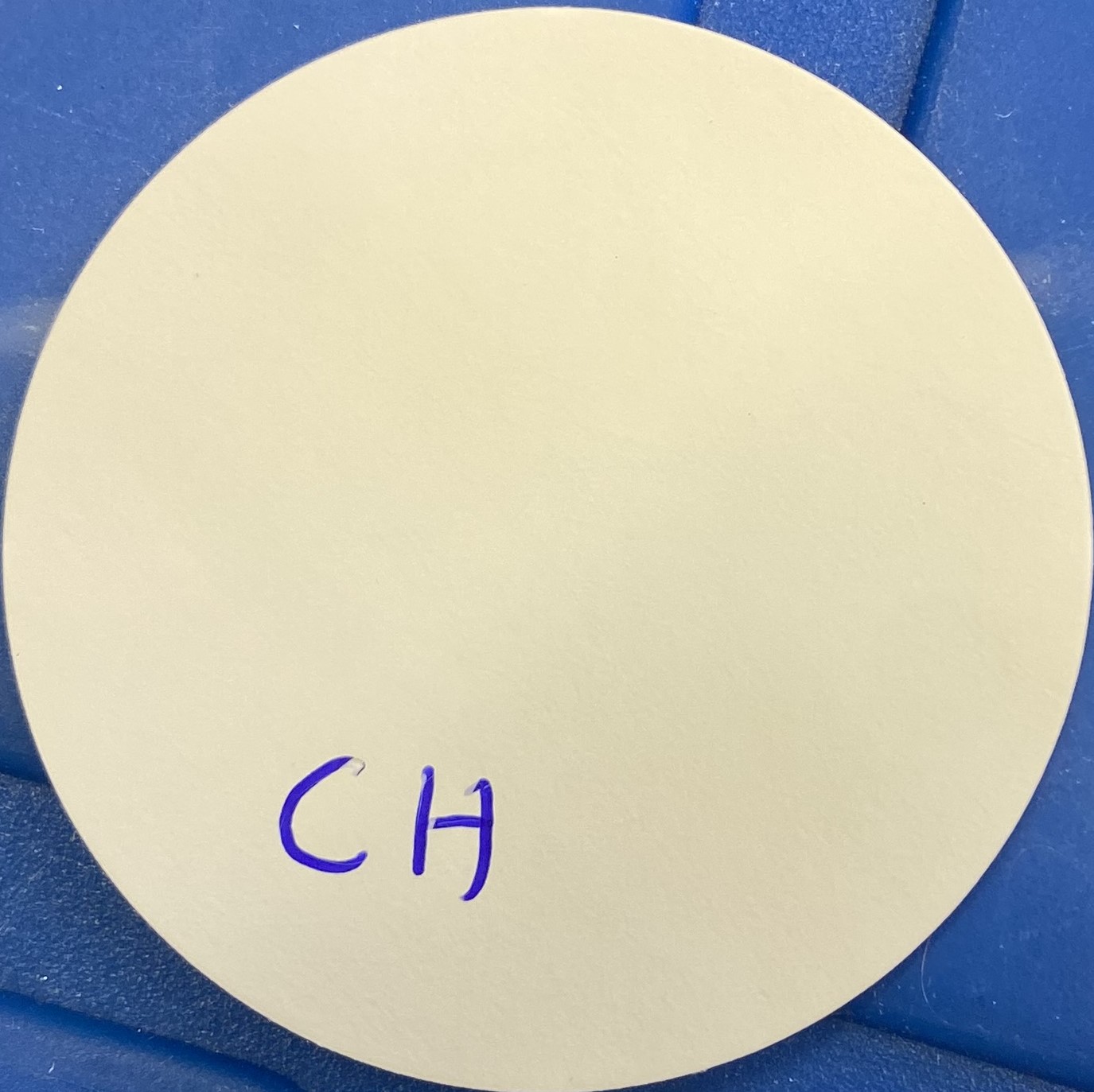

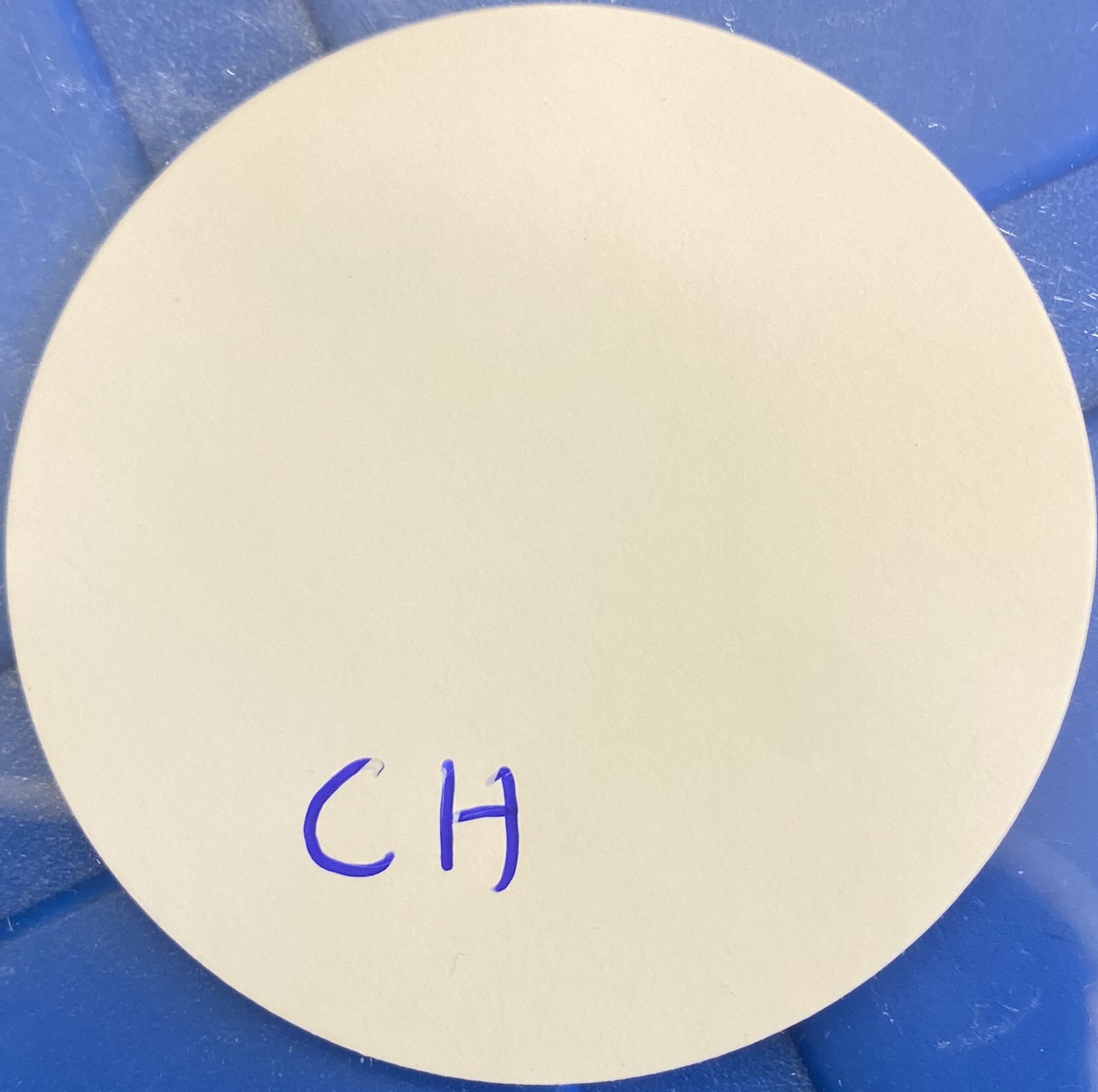

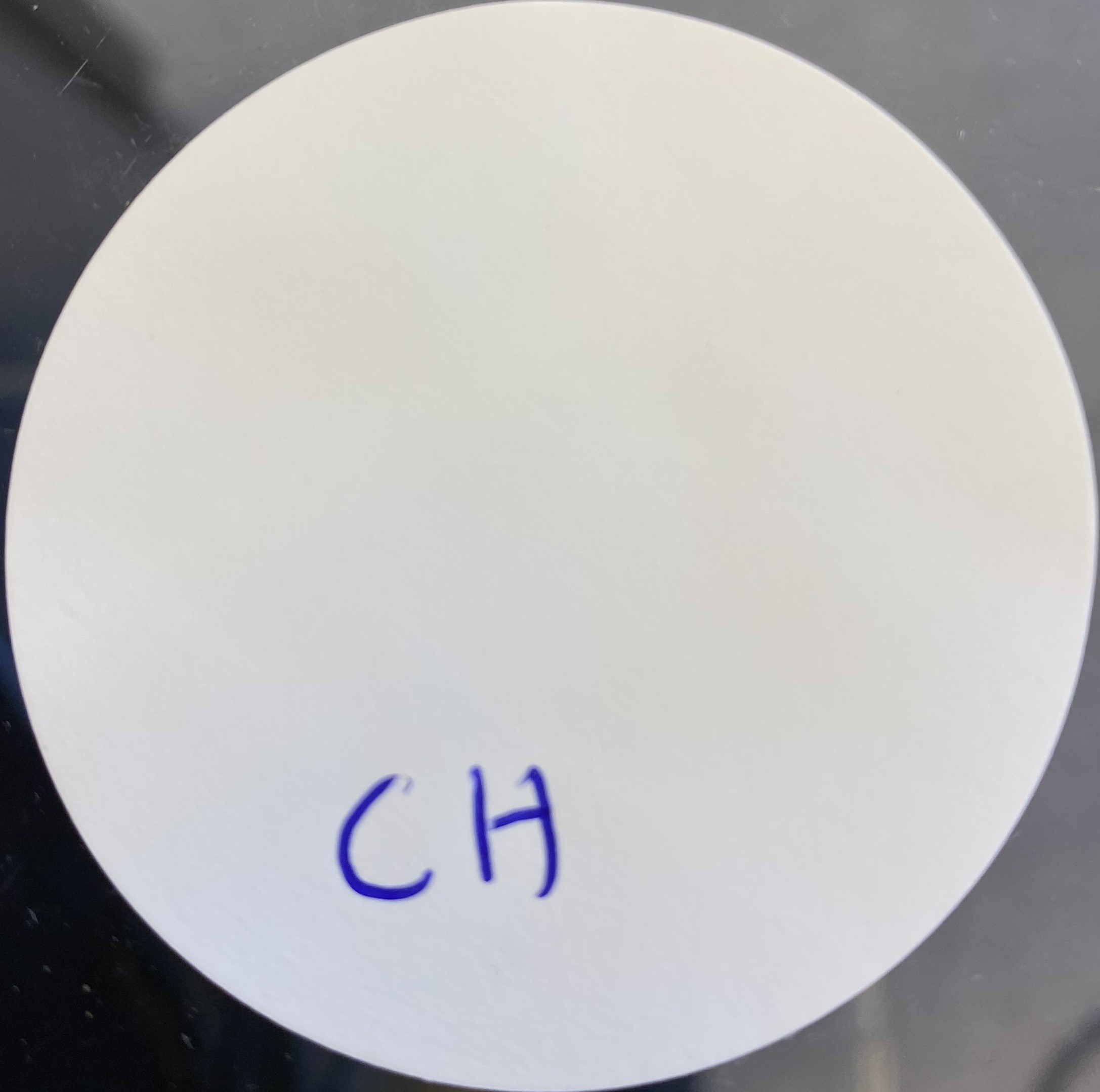

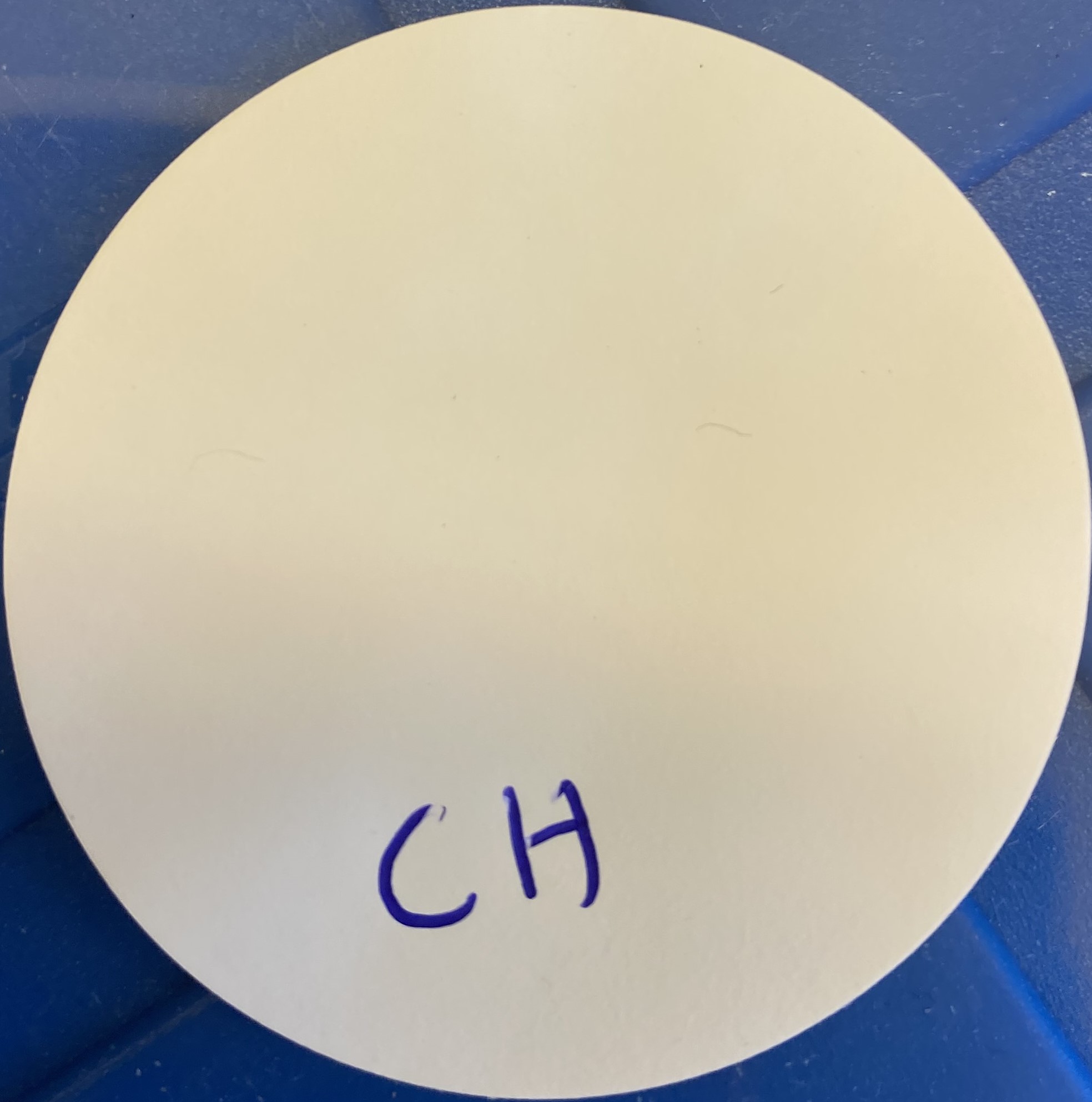


**Fig. S6** ASTM D4740 spot test of CH (a) 1 min, (b) 30 min, (c) 60 min, (d) 20 h, (e) 24h (left to right)

**Spot No. 2**


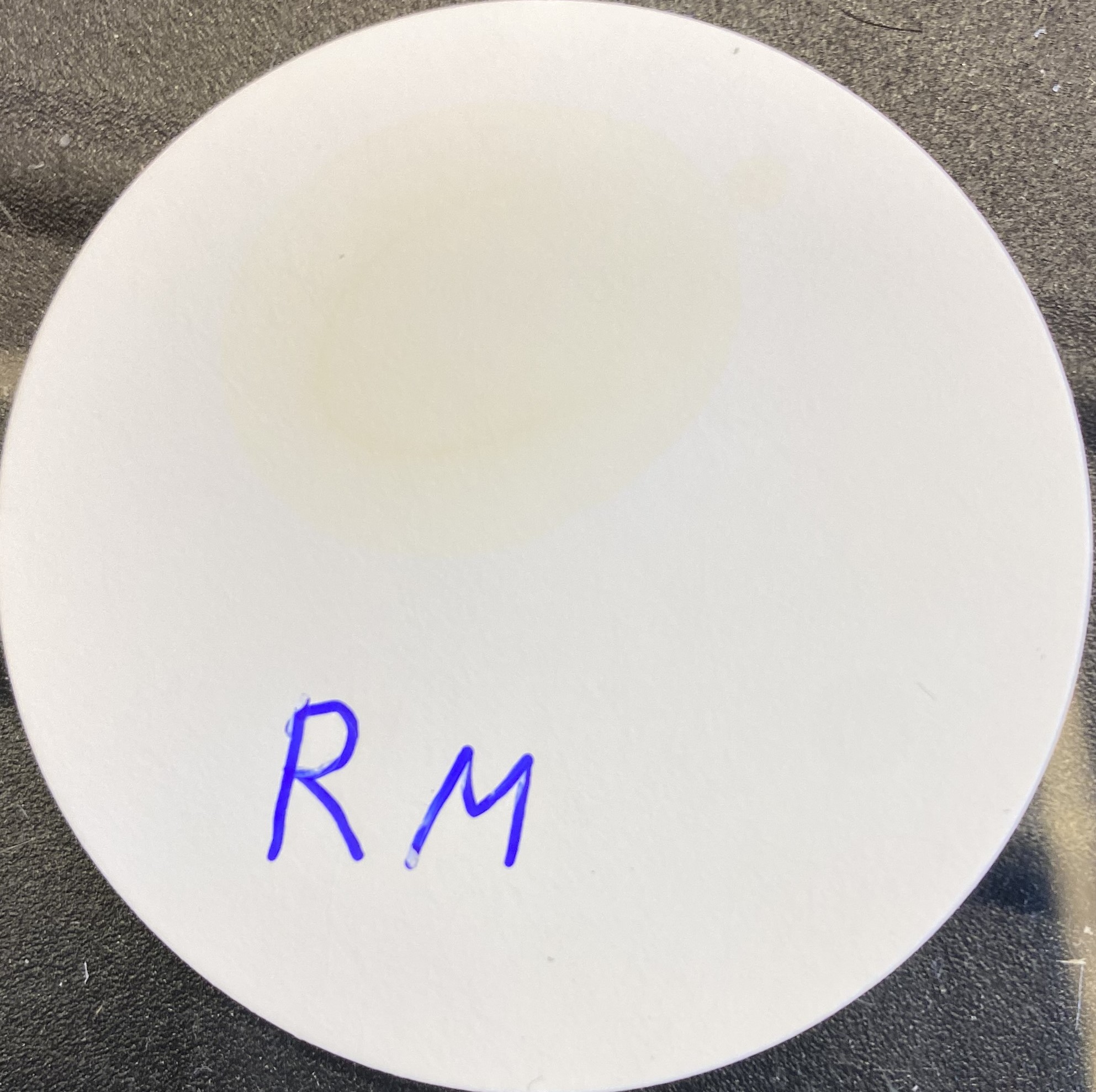

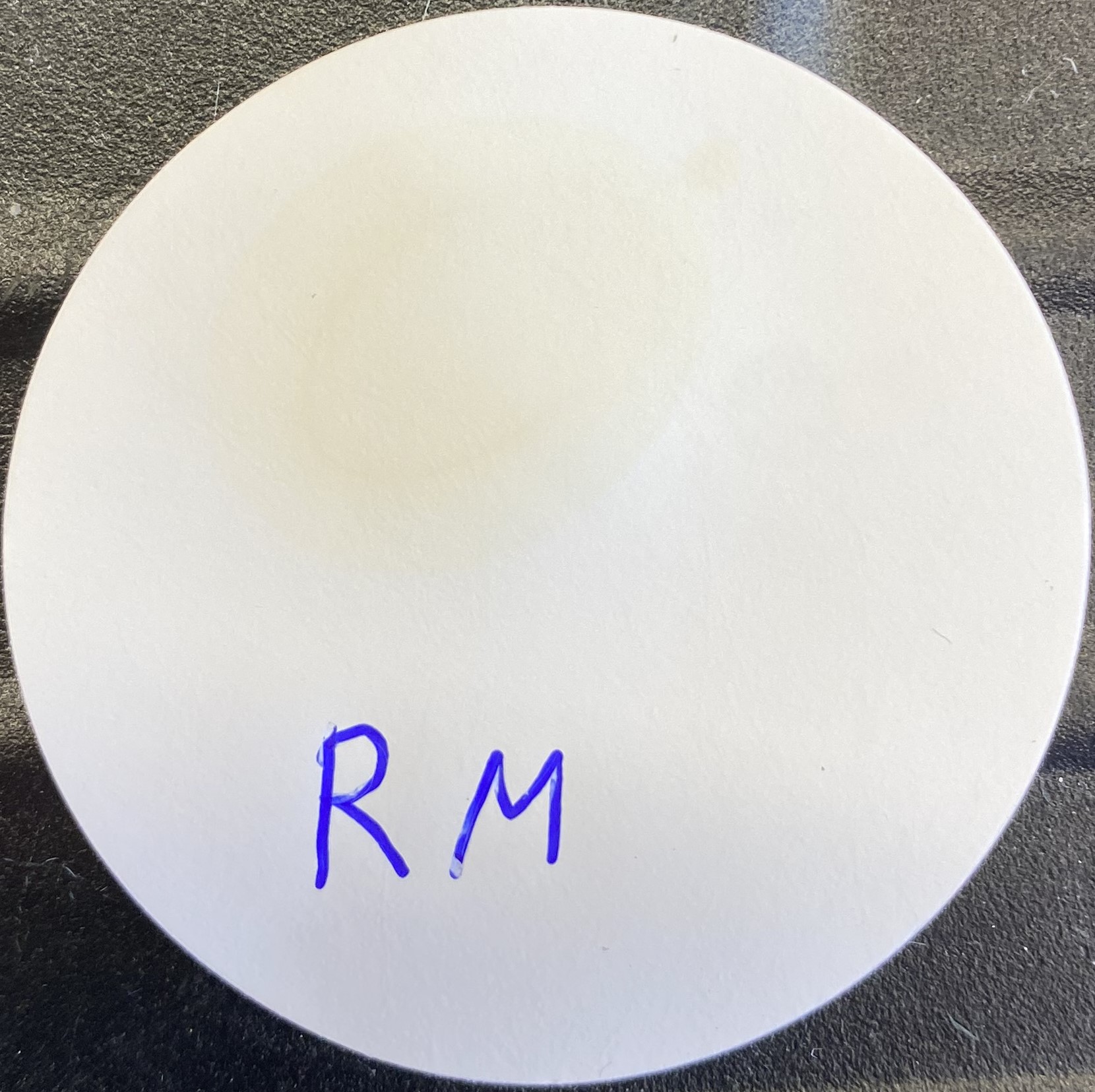

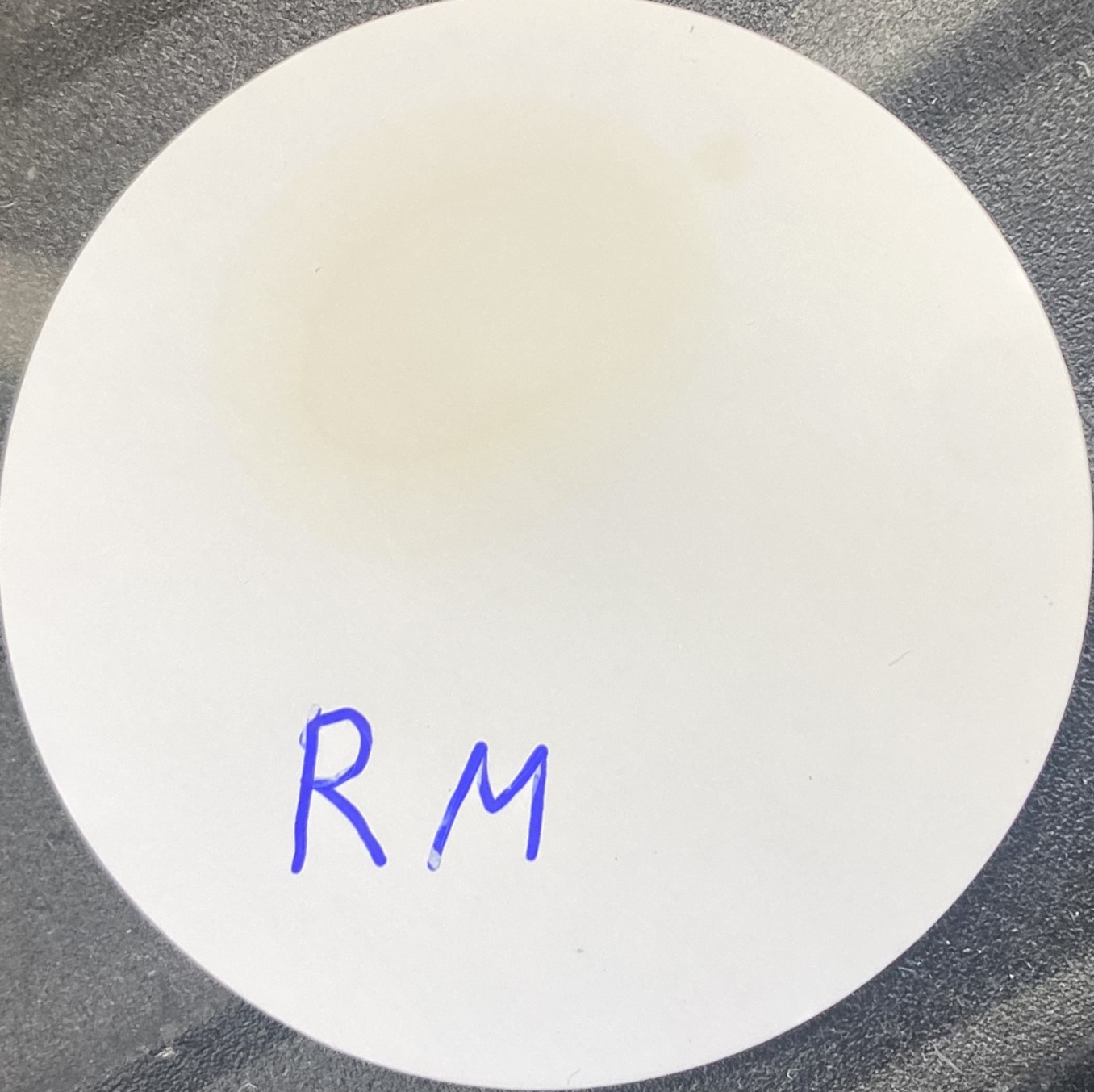

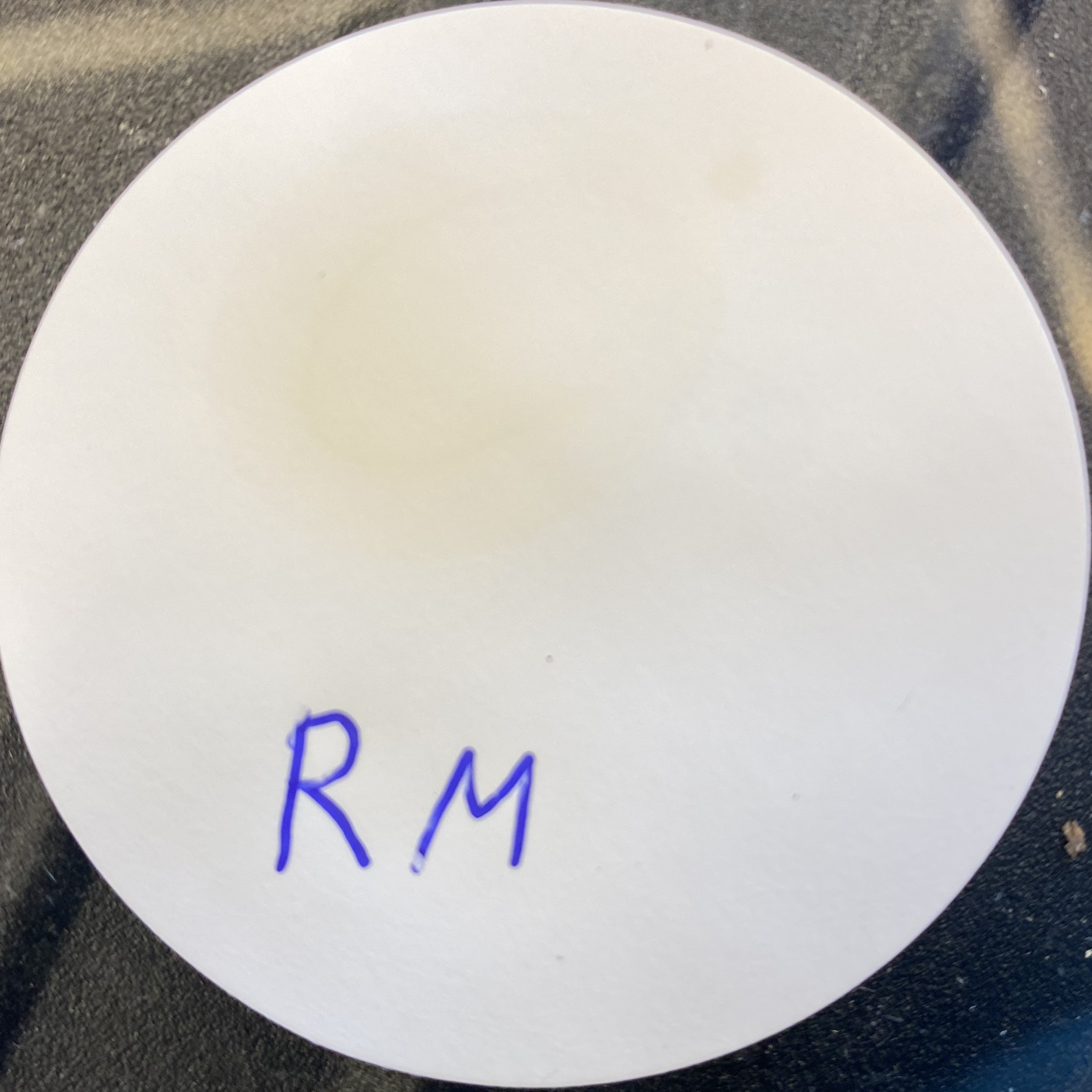

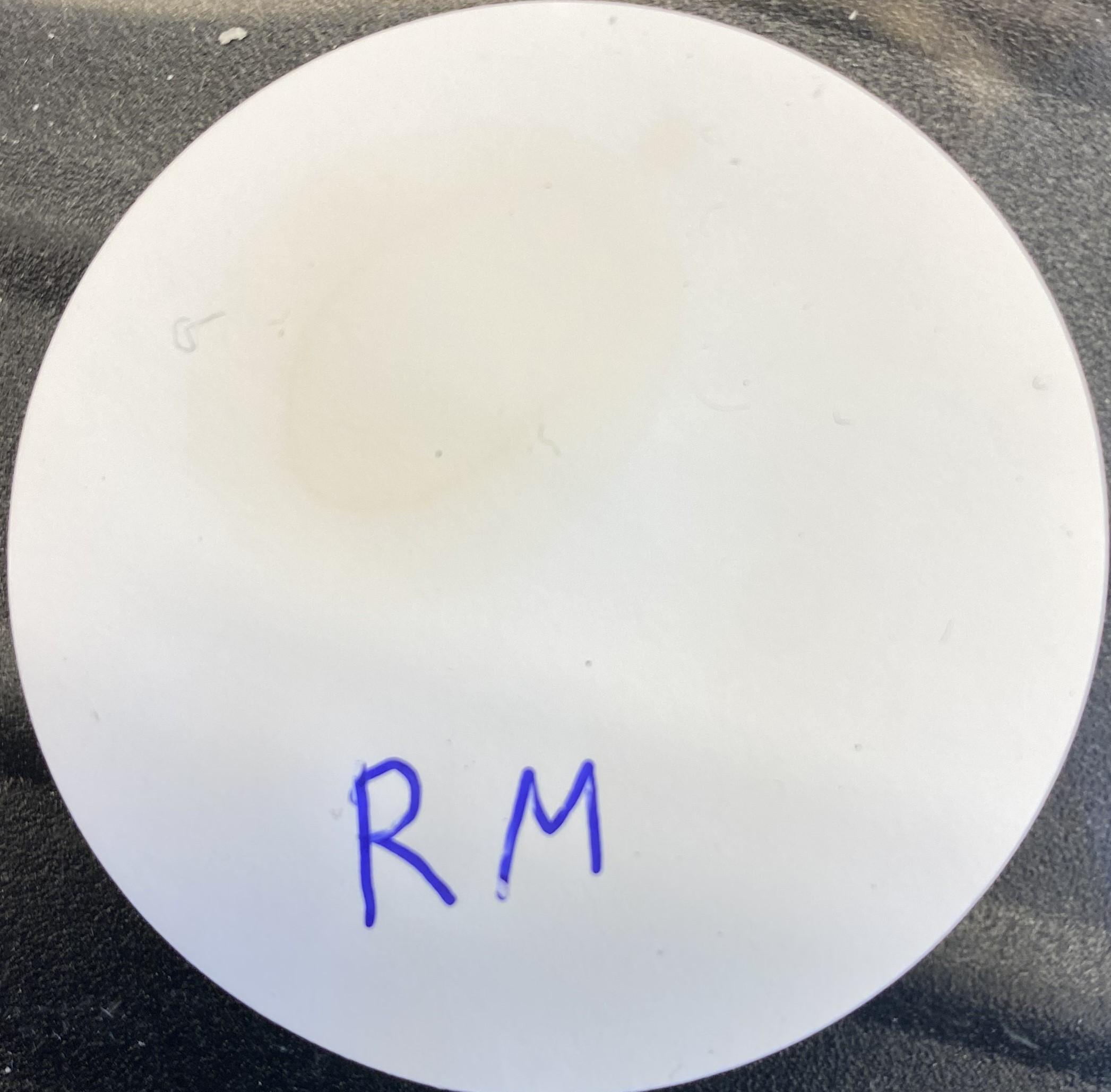


**Fig. S7** ASTM D4740 spot test of RM (a) 1 min, (b) 30 min, (c) 60 min, (d) 20 h, (e) 24h (left to right)

**Spot No. 1**


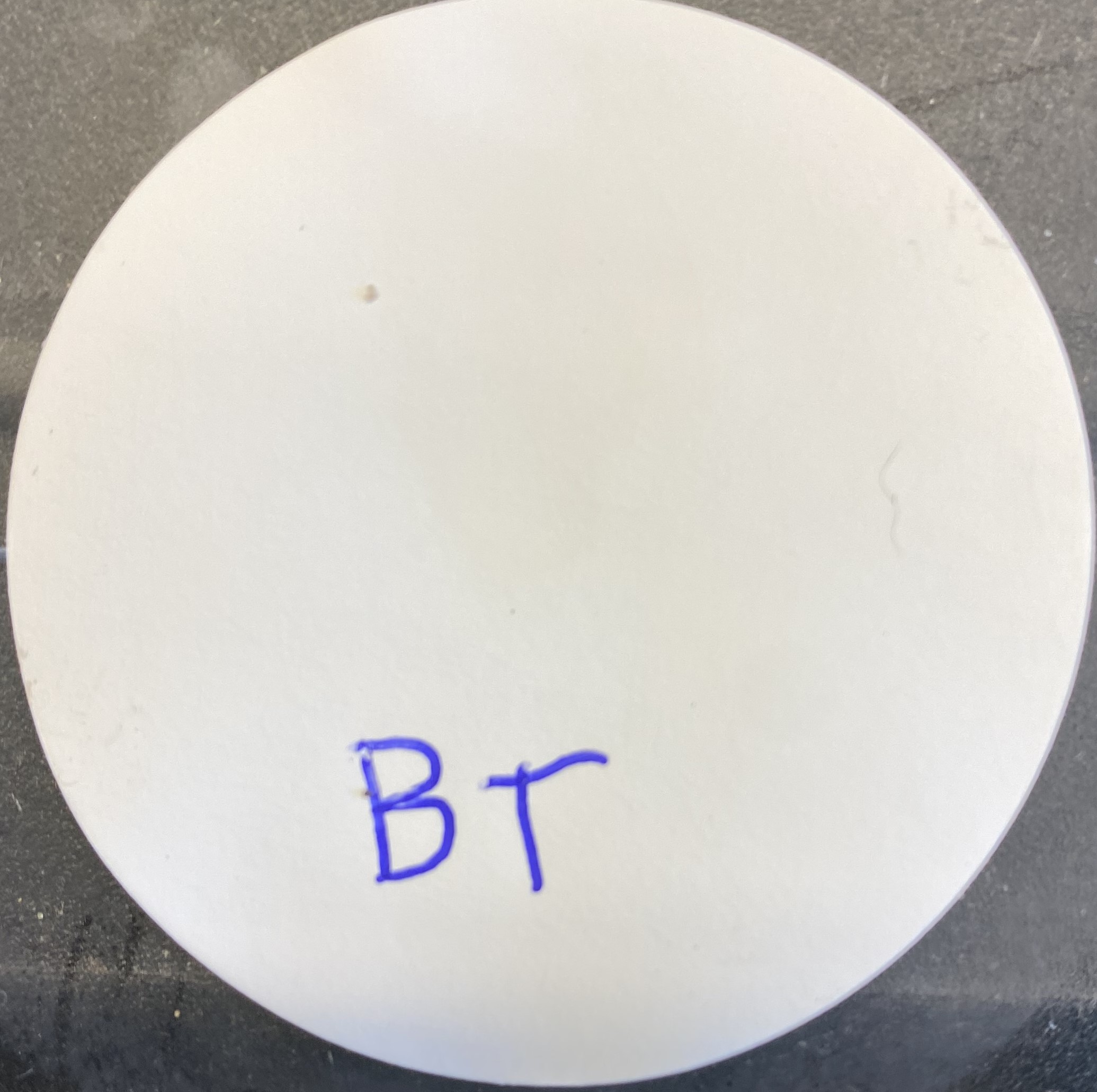

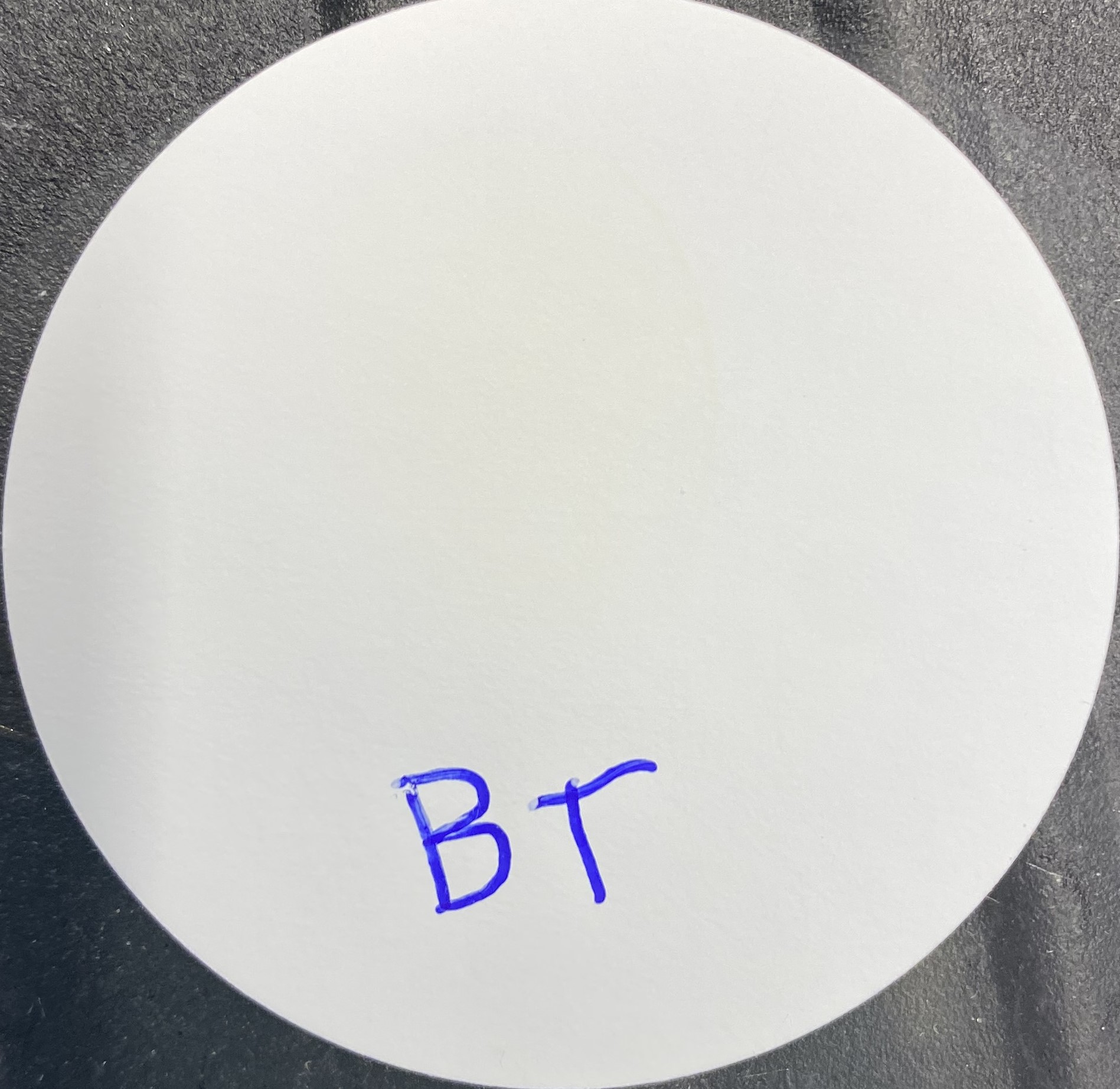

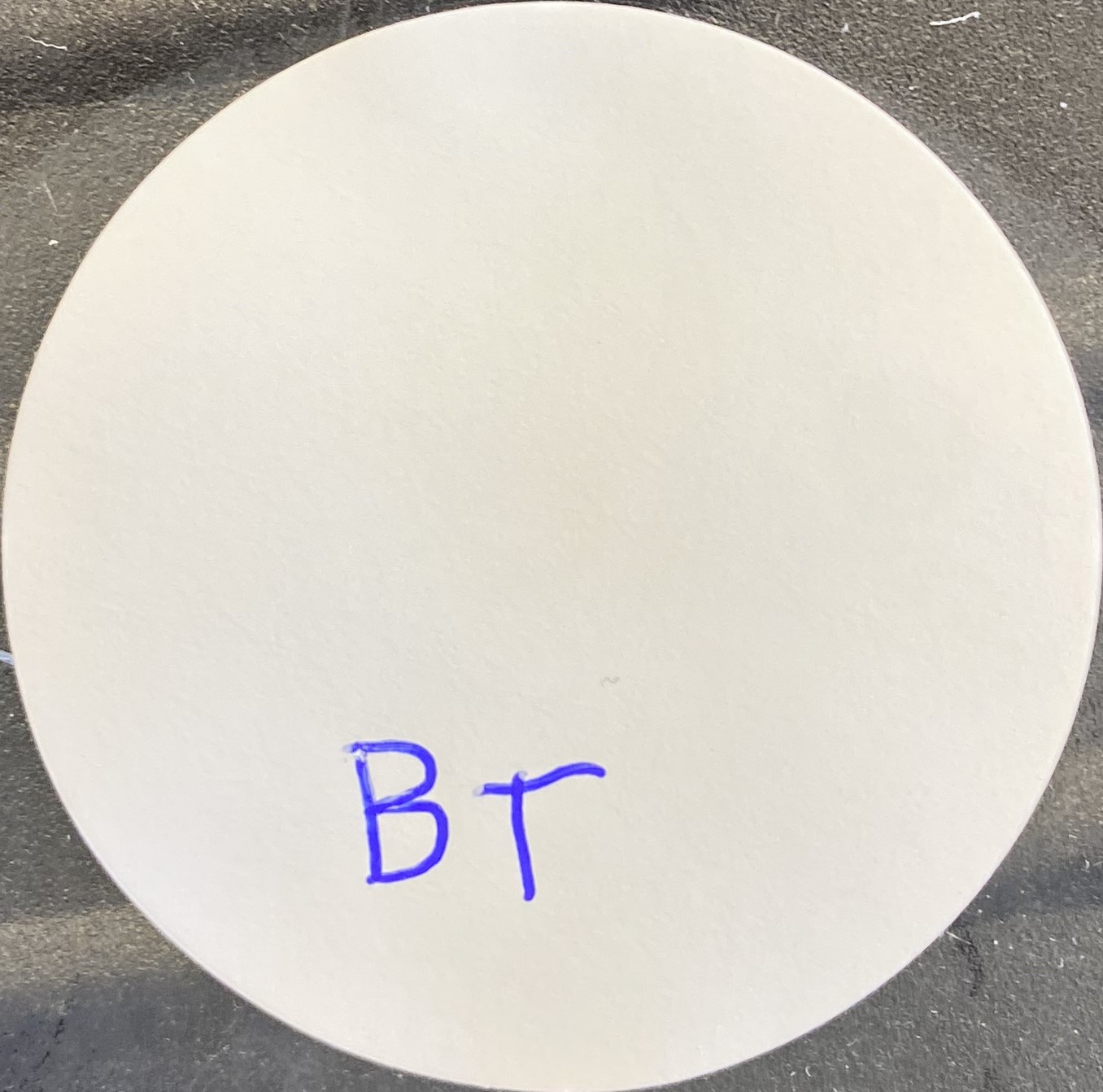

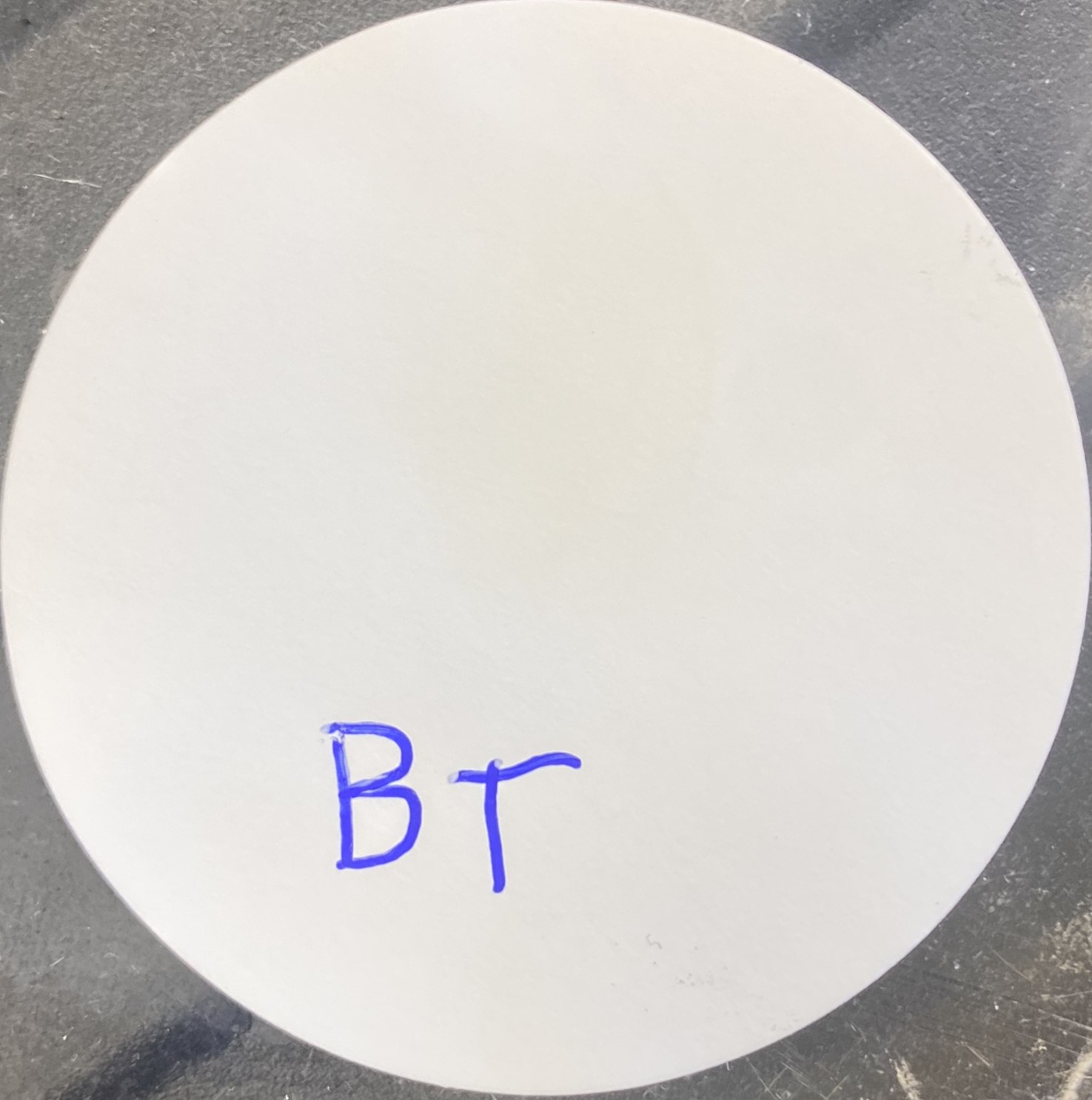

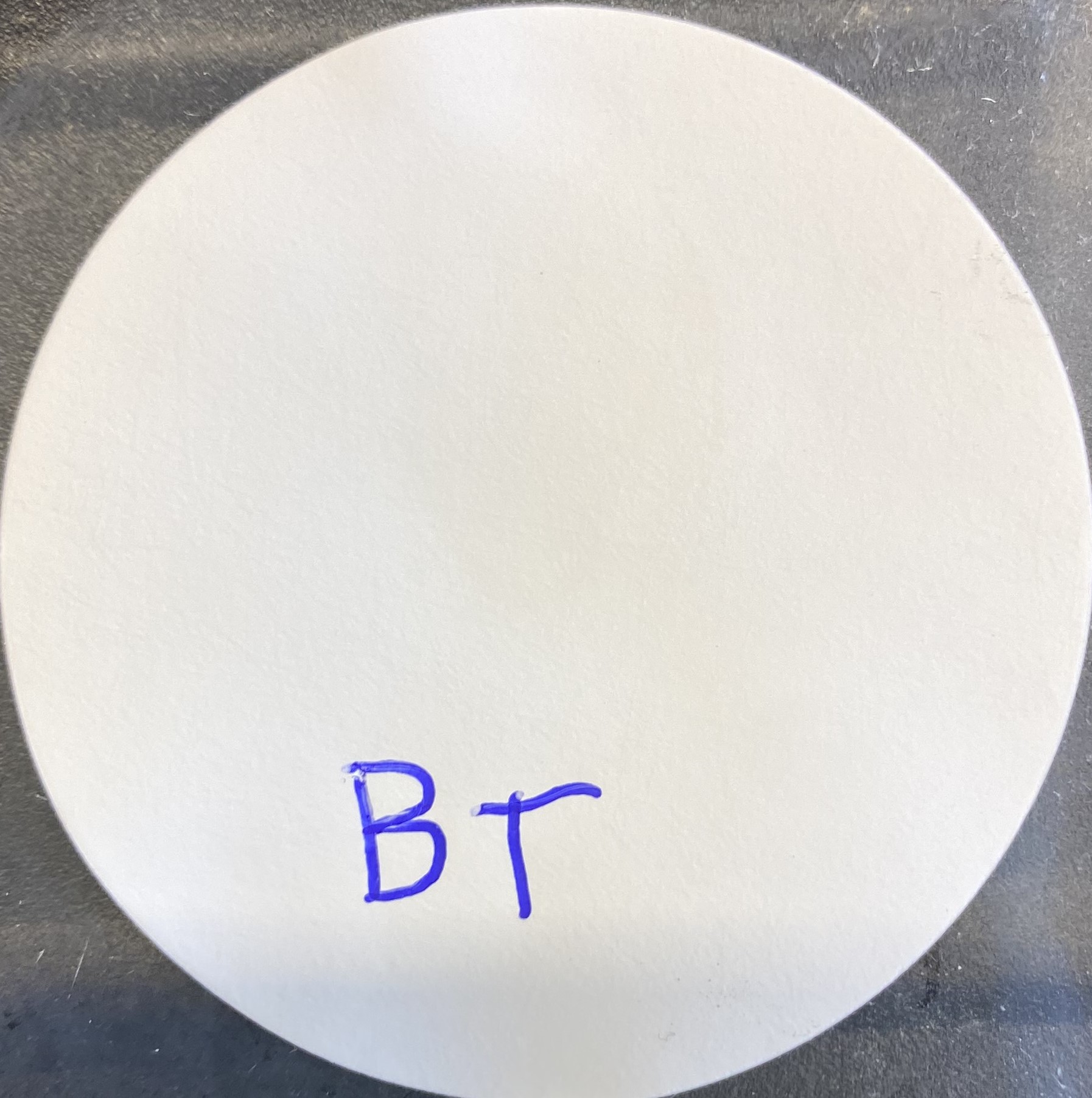


**Fig. S8** ASTM D4740 spot test of BT (a) 1 min, (b) 30 min, (c) 60 min, (d) 20 h, (e) 24h (left to right)

**Spot No. 3**


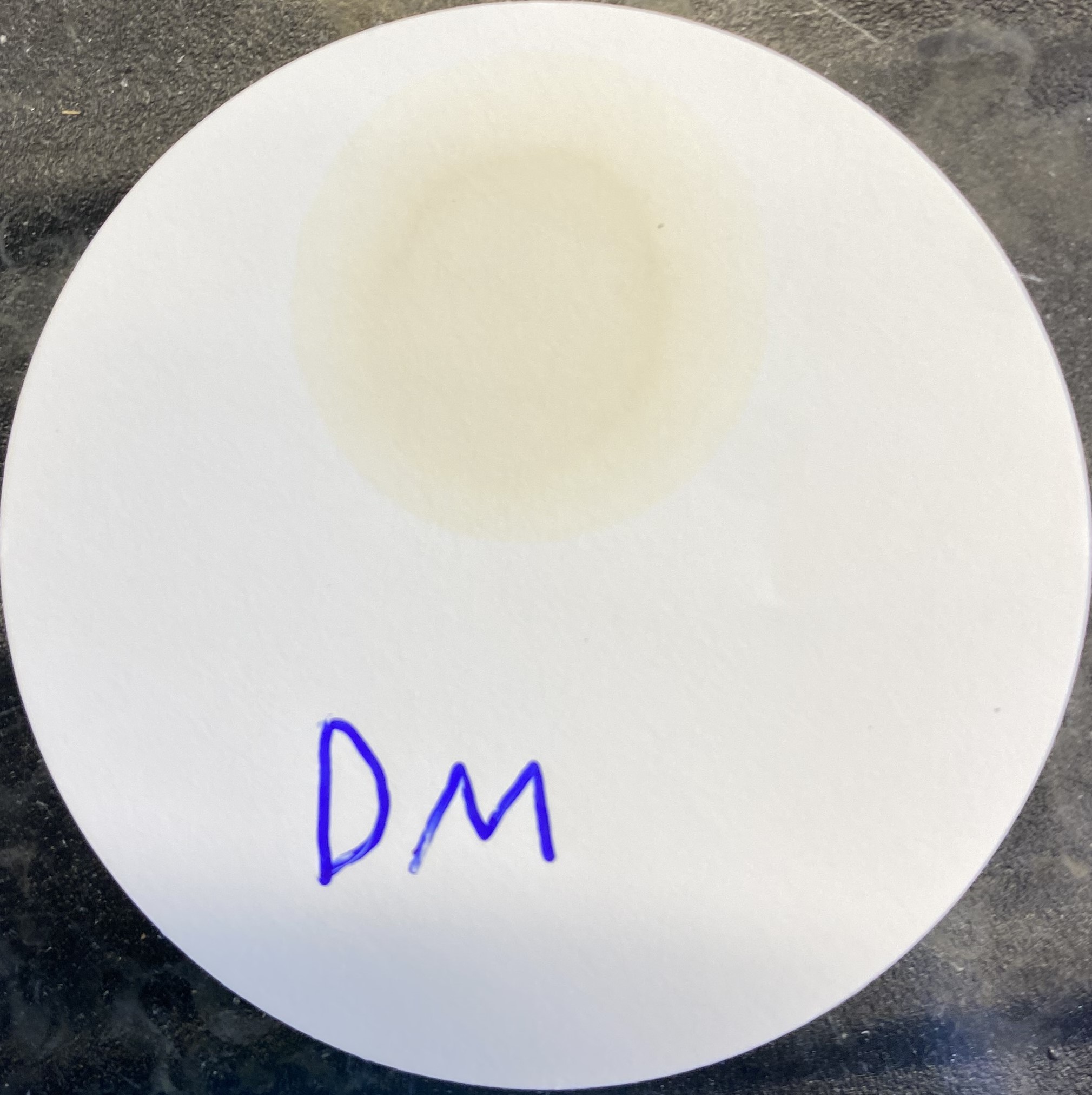

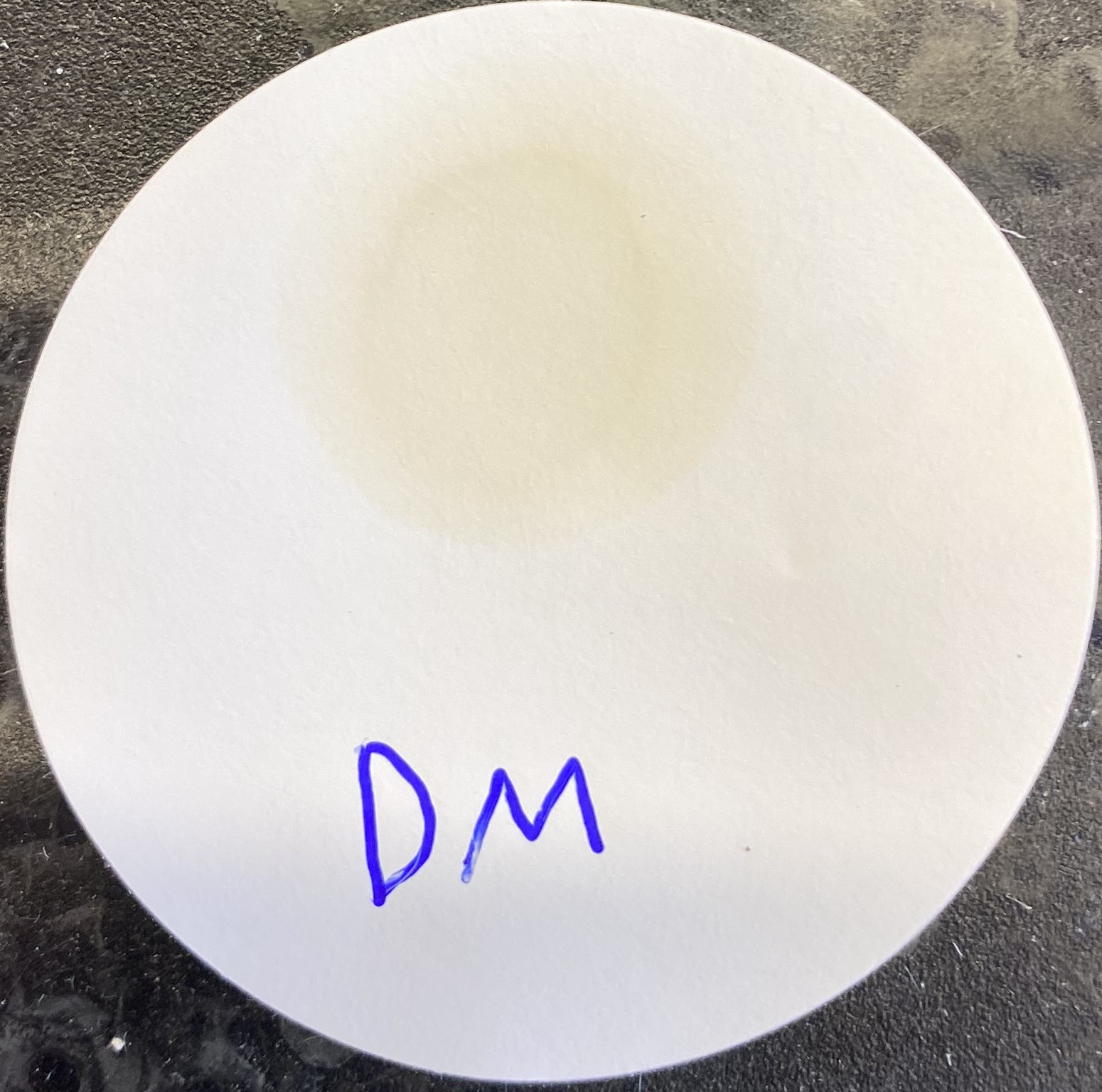

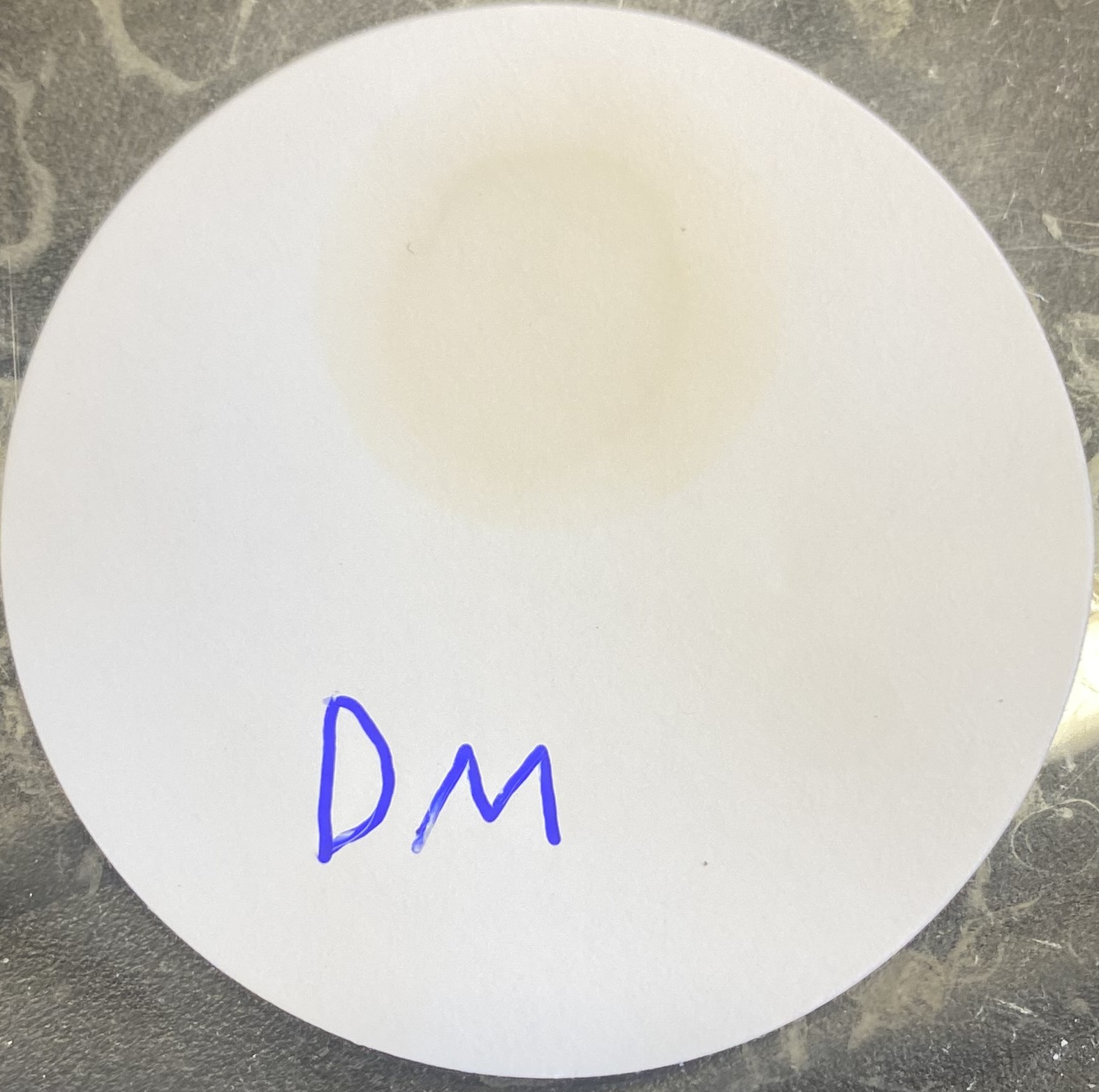

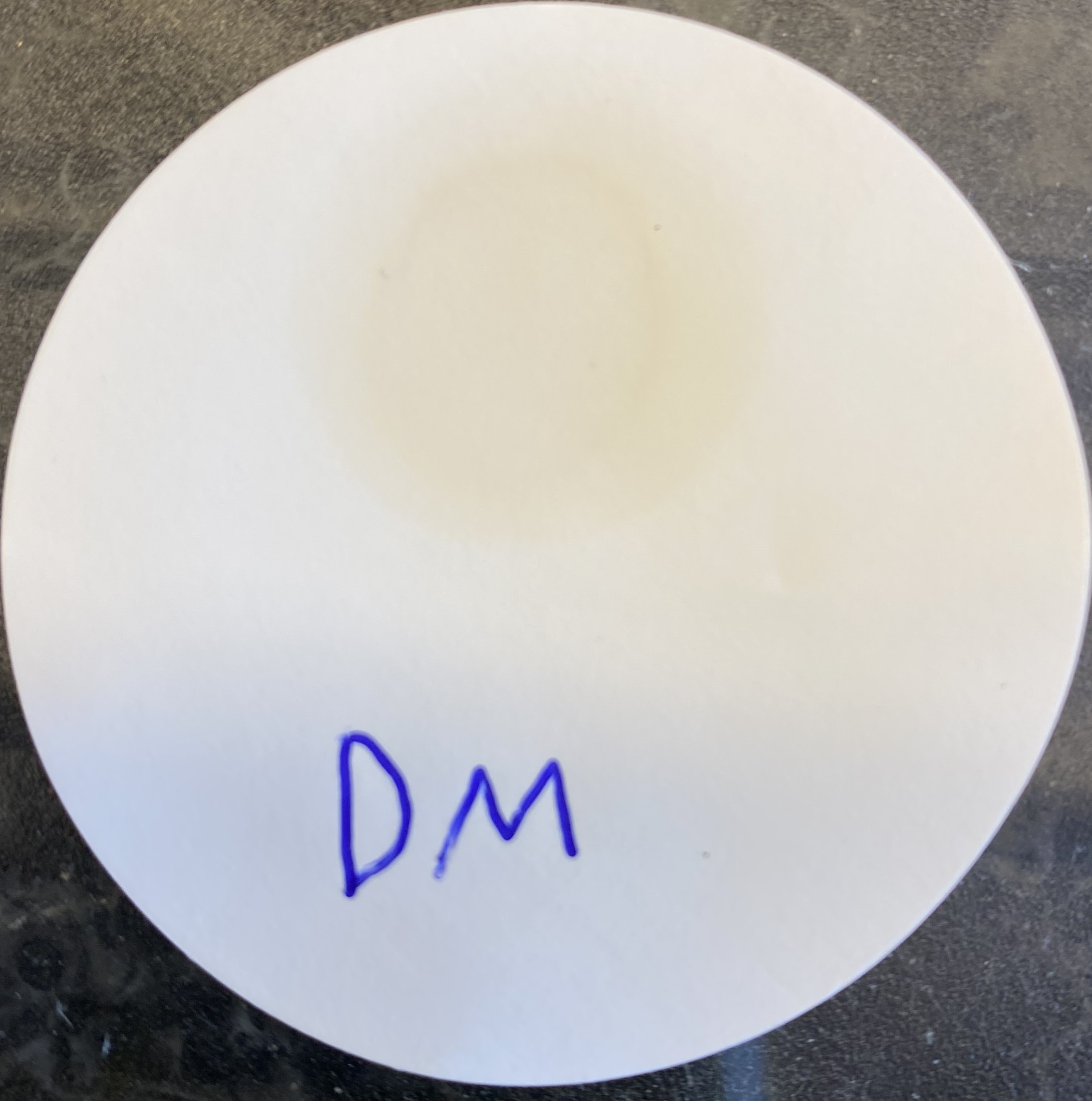

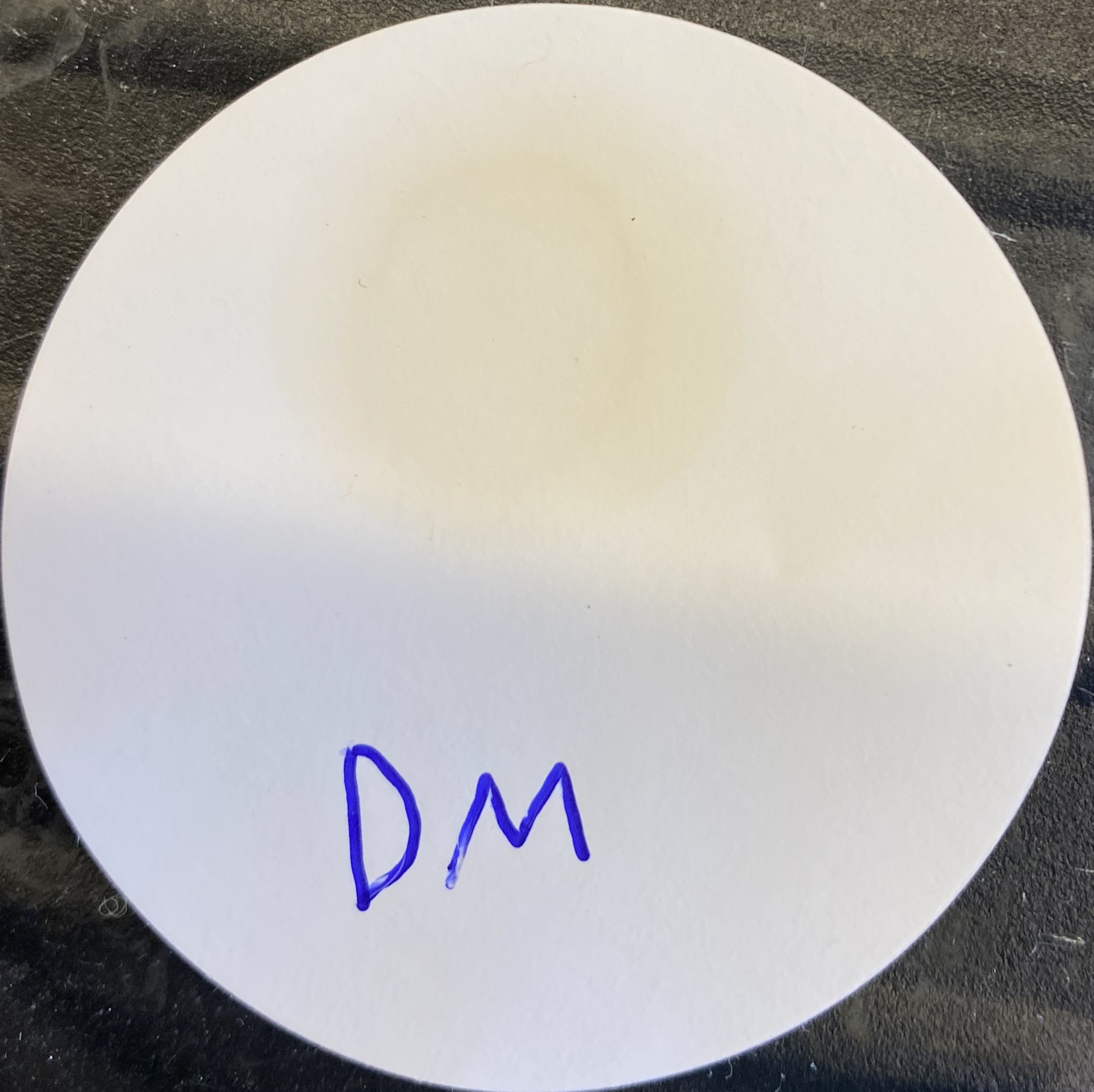


**Fig. S9** ASTM D4740 spot test of DM (a) 1 min, (b) 30 min, (c) 60 min, (d) 20 h, (e) 24h (left to right)

**Spot No. 2**


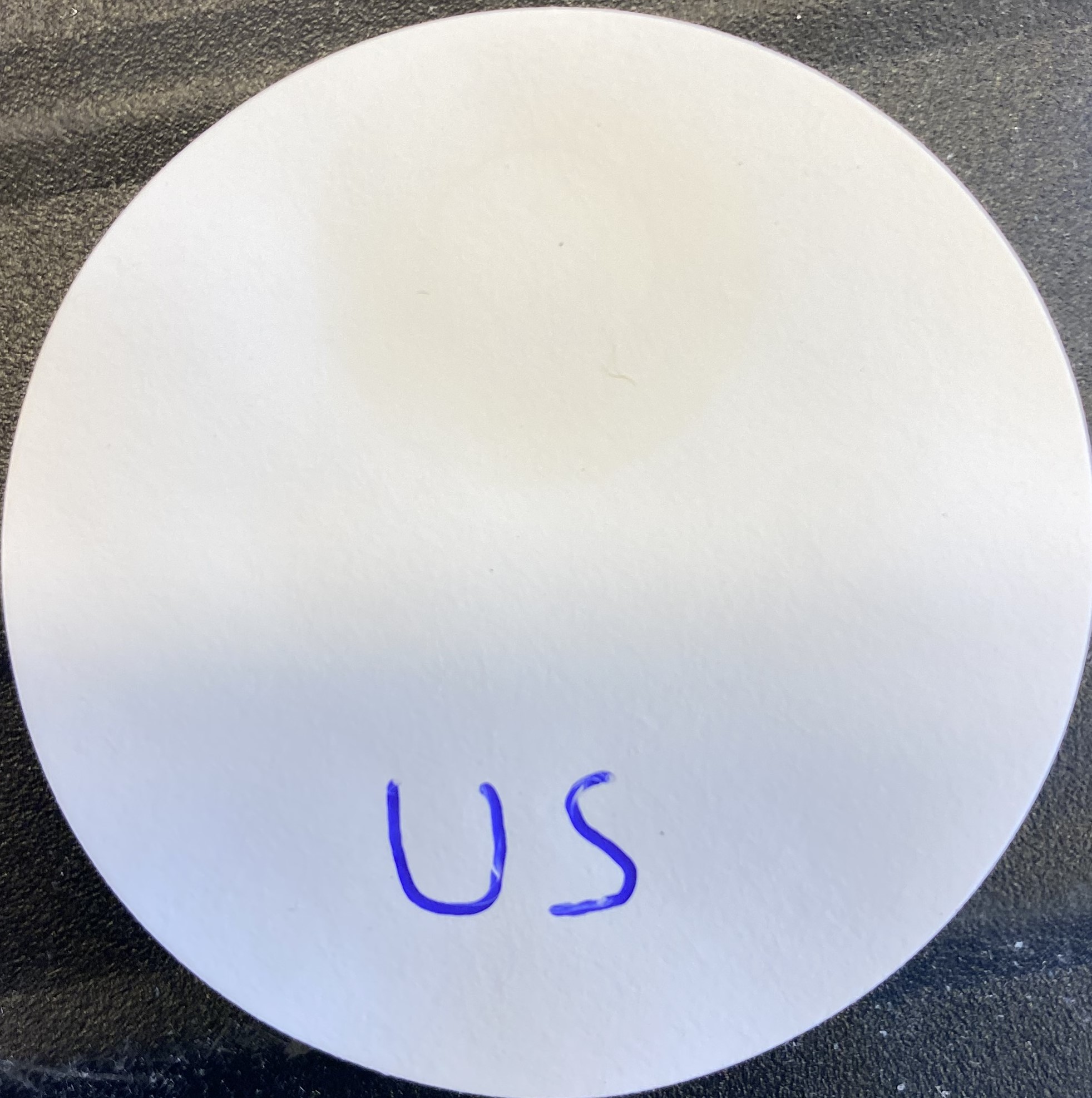

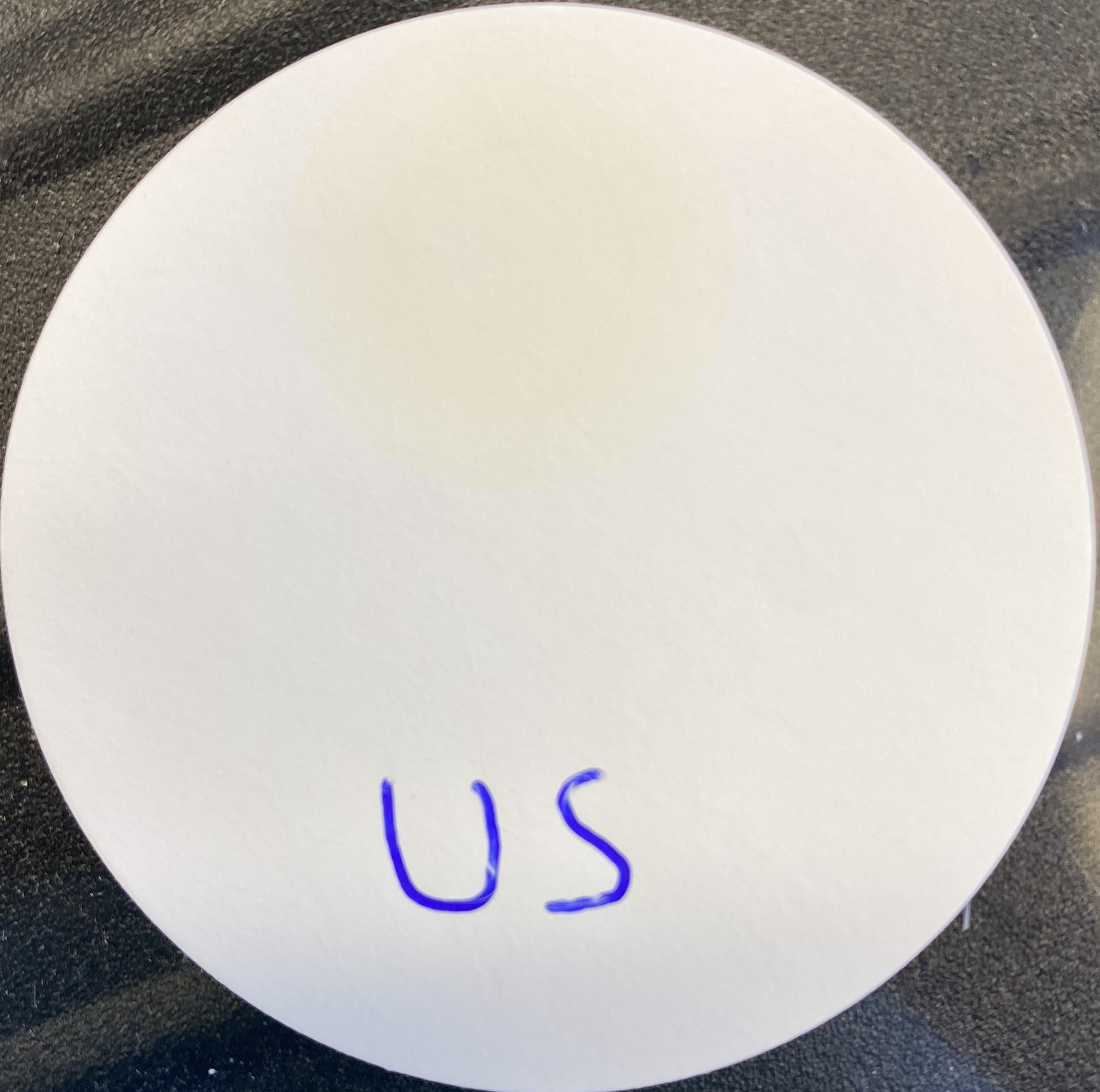

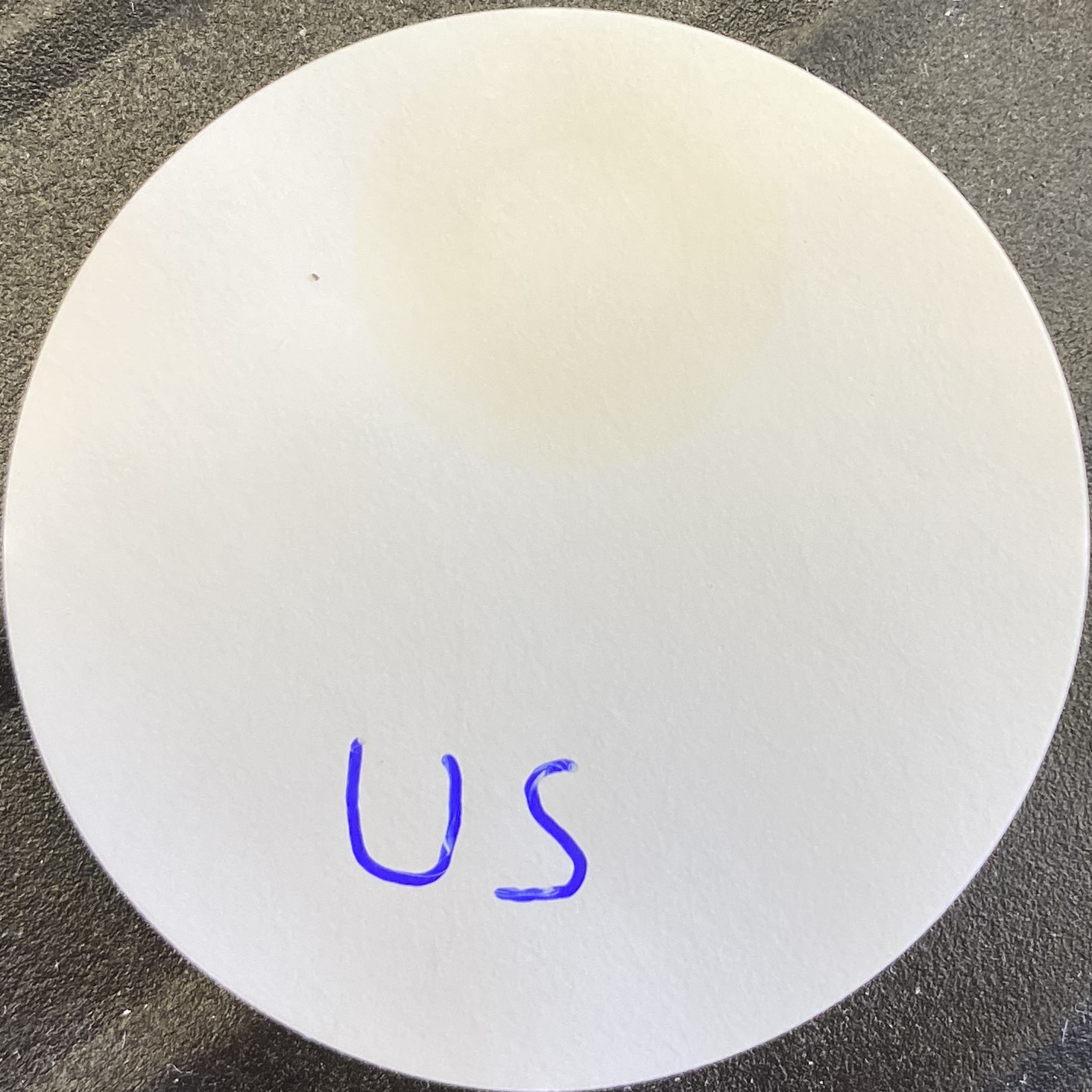

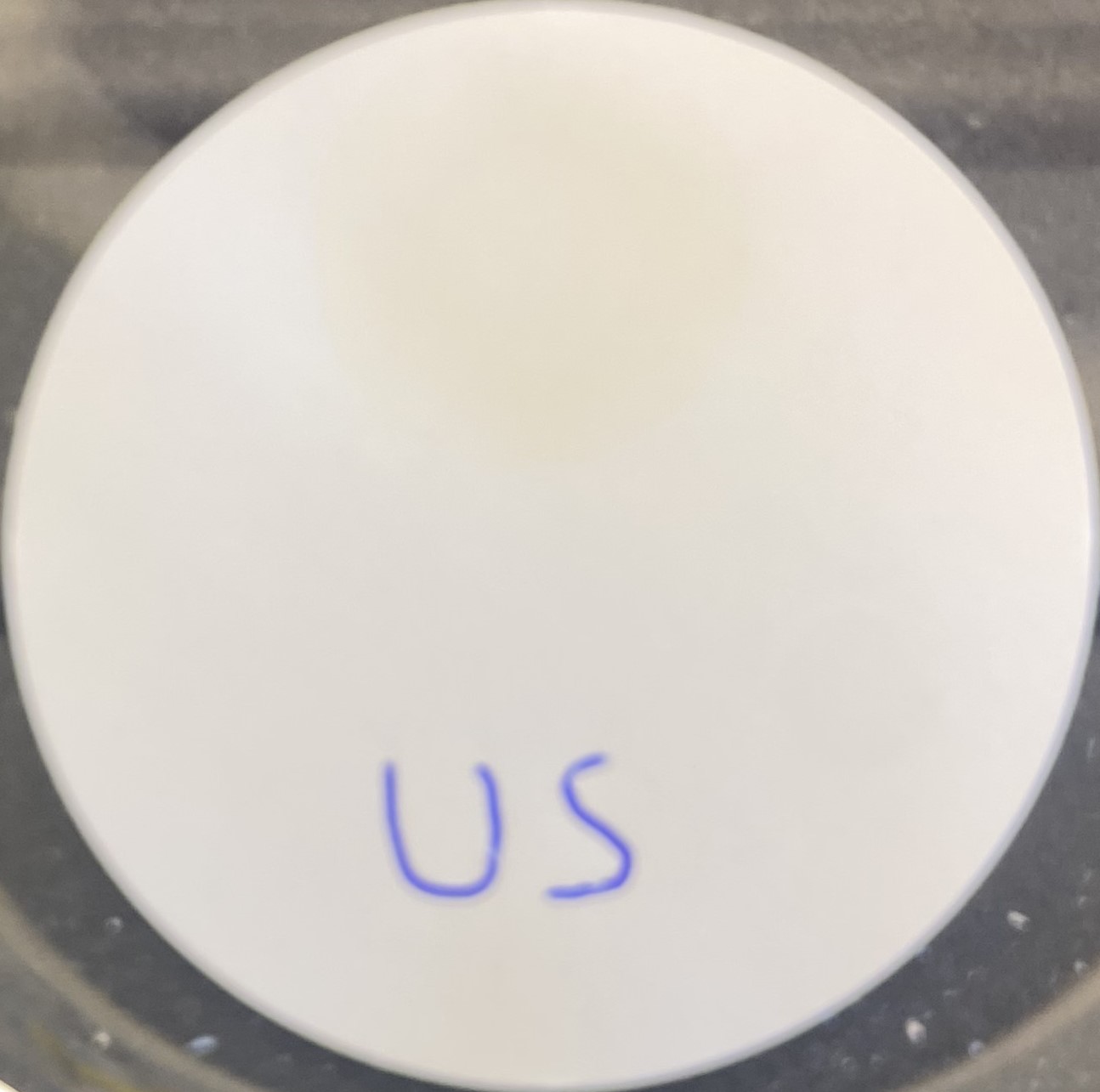

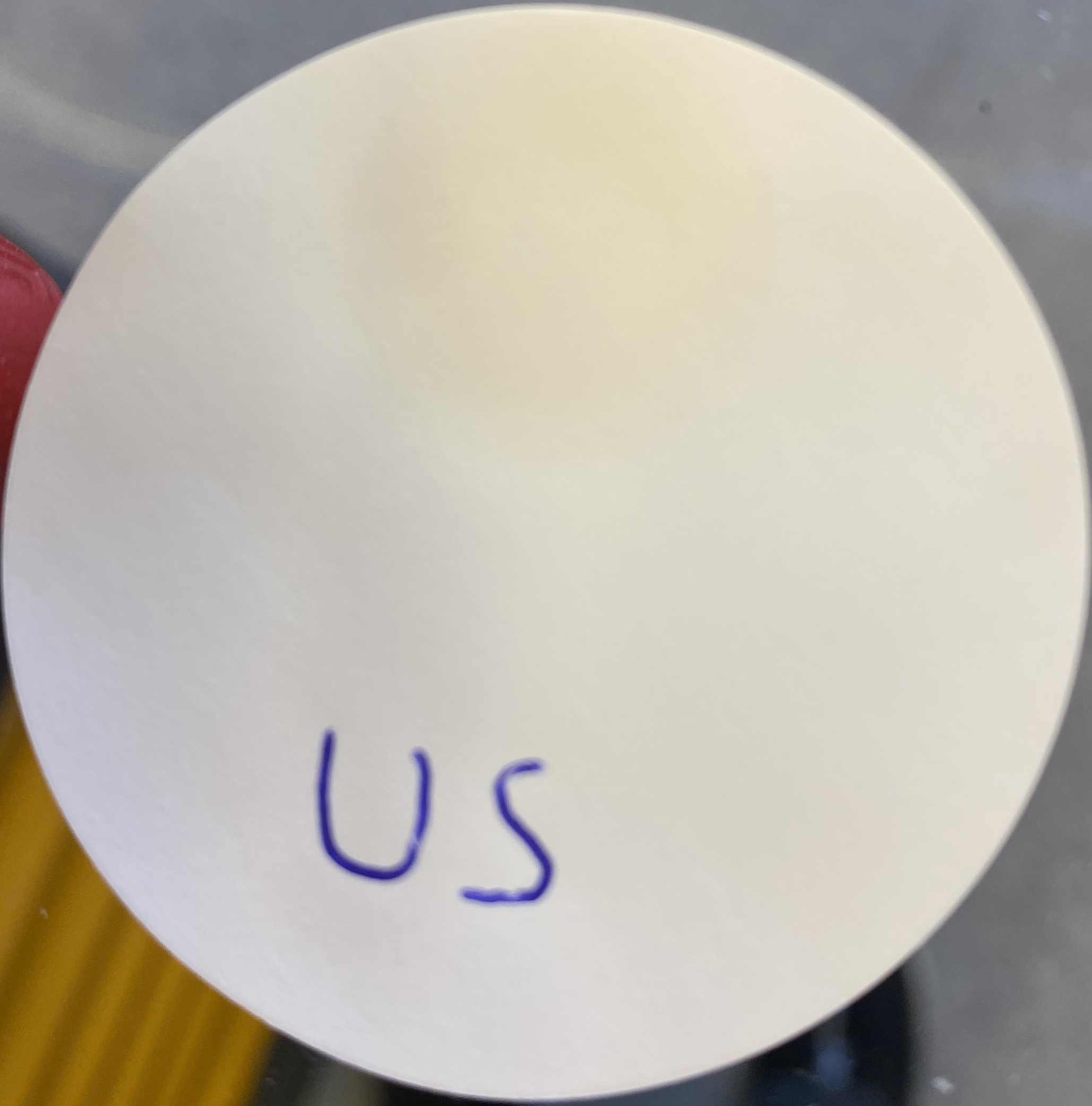


**Fig. S10** ASTM D4740 spot test of US (a) 1 min, (b) 30 min, (c) 60 min, (d) 20 h, (e) 24h (left to right)

**Spot No. 2**


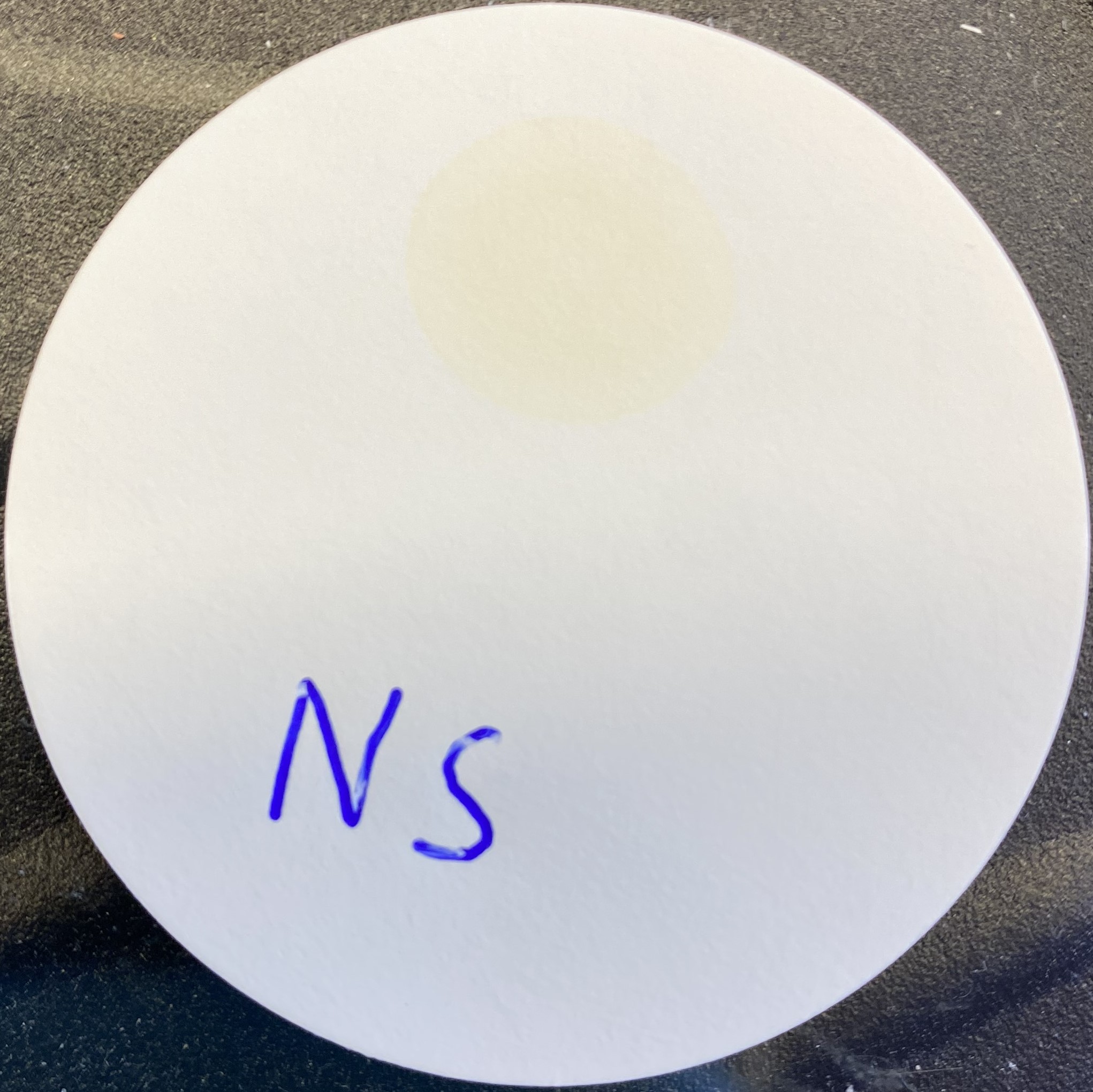


**Fig. S11** ASTM D4740 spot test of NS (a) 1 min, (b) 30 min, (c) 60 min, (d) 20 h, (e) 24h (left to right)

**References**

Yildiz G, Ronsse F, Prins W, Assink D, Gerritsen L, van Duren R & Rosso-Vasic M. (2011). Catalytic fast pyrolysis of biomass. Proceedings of the 19th European Biomass Conference and Exhibition, June 10 - 14, Berlin, Germany, 1145 - 1148. Doi: 10.5071/19thEUBCE2011-OB8.1

Van de Beld L, Muggen G. (2015). Empryo: Implementation of a commercial scale fast pyrolysis plant in the Netherlands, 23^rd^ Biomass Conference and Exhibition, June 1-4, Vienna, Austria.
